# Synthesis, fungal biotransformation, and evaluation of the antimicrobial potential of chalcones with a chlorine atom

**DOI:** 10.1038/s41598-024-65054-9

**Published:** 2024-07-01

**Authors:** Agnieszka Krawczyk-Łebek, Barbara Żarowska, Monika Dymarska, Tomasz Janeczko, Edyta Kostrzewa-Susłow

**Affiliations:** 1 Department of Food Chemistry and Biocatalysis, Faculty of Biotechnology and Food Science, Wrocław University of Environmental and Life Sciences, Wrocław, Poland; 2 Department of Biotechnology and Food Microbiology, Faculty of Biotechnology and Food Science, Wrocław University of Environmental and Life Sciences, Wrocław, Poland

**Keywords:** Biotransformations, Chalcone derivatives, Chalcones with a chlorine atom, Glycosylation, *Beauveria bassiana*, *Isaria fumosorosea*, Antimicrobial activity, Biotechnology, Chemical biology

## Abstract

Chalcones are intermediate products in the biosynthesis of flavonoids, which possess a wide range of biological properties, including antimicrobial and anticancer activities. The introduction of a chlorine atom and the glucosyl moiety into their structure may increase their bioavailability, bioactivity, and pharmacological use. The combined chemical and biotechnological methods can be applied to obtain such compounds. Therefore, 2-chloro-2′-hydroxychalcone and 3-chloro-2′-hydroxychalcone were synthesized and biotransformed in cultures of two strains of filamentous fungi, i.e. *Isaria fumosorosea* KCH J2 and *Beauveria bassiana* KCH J1.5 to obtain their novel glycosylated derivatives. Pharmacokinetics, drug-likeness, and biological activity of them were predicted using cheminformatics tools. 2-Chloro-2′-hydroxychalcone, 3-chloro-2′-hydroxychalcone, their main glycosylation products, and 2′-hydrochychalcone were screened for antimicrobial activity against several microbial strains. The growth of *Escherichia coli* 10,536 was completely inhibited by chalcones with a chlorine atom and 3-chlorodihydrochalcone 2′-*O*-*β*-d-(4″-*O*-methyl)-glucopyranoside. The strain *Pseudomonas aeruginosa* DSM 939 was the most resistant to the action of the tested compounds. However, chalcone aglycones and glycosides with a chlorine atom almost completely inhibited the growth of bacteria *Staphylococcus aureus* DSM 799 and yeast *Candida albicans* DSM 1386. The tested compounds had different effects on lactic acid bacteria depending on the tested species. In general, chlorinated chalcones were more effective in the inhibition of the tested microbial strains than their unchlorinated counterparts and aglycones were a little more effective than their glycosides.

## Introduction

Chalcones occur commonly in vegetables, fruits, and edible plants, and possess a privileged structure in medicinal chemistry. They have a common chemical scaffold consisting of two aromatic rings joined by a three-carbon α, β-unsaturated carbonyl system. Their hydrogenated derivatives, i.e., dihydrochalcones, such as phlorizin, are also widespread in nature^1–3^. Furthermore, chalcones can be successfully synthesized and functionalized using the Claisen–Schmidt condensation method^4–7^. Chalcones exhibit many biological activities such as antimicrobial^8–13^, antiprotozoal^13–15^, anti-inflammatory and antioxidant^1,12,13,15,16^, anticancer^1,6,7,12,15,17–19^, and anti-diabetic^1,20–22^.

Modulation of the biological activity and bioavailability of these compounds can be achieved by substitution with a chlorine atom or a glucose moiety, among others. It has been established empirically in many studies that the introduction of a chlorine atom can modulate the biological activity of the molecule leading to its improvement or abolition^23^. The in vitro assays show that flavonoids possess hypochlorite scavenging activity. This is beneficial because hypochlorous acid is a hly reactive oxidant produced by activated phagocytes and can damage host tissues during long-term inflammation^24–26^. The mono- and dichloroflavonoid derivatives formed in such a process retain and even strengthen their antioxidant potential. Synthetic chlorinated flavonoids such as 8-chloro-3′,4′,5,7-tetrahydroxyflavone and 3-chloro-3′,4′, 5,7-tetrahydroxyflavone, as well as chlorinated by hypochlorous acid natural flavonoids, i.e., luteolin, rutin, and quercetin, were more effective in regulating the viability of neutrophils and their release of reactive oxygen species than their non-chlorinated counterparts ^24,27^. Similarly, the biological activity, e.g. antimicrobial activity of chalcones can be positively affected by chromatophores such as chlorine atoms. Unfortunately, there are few studies on the influence of the chlorine substituent on antimicrobial activity. In the work of Prasad et al., the chalcone derivatives substituted with halogens (chlorine and/or bromine and/or fluorine) showed significant antimicrobial activity against gram-positive bacteria *Bacillus pumilus*, *Bacillus subtilis*, and gram-negative bacteria *Escherichia coli*. Moreover, 4-chloro-4′-bromochalcone showed a h inhibitory effect against fungi *Aspergillus niger* and *Rhizopus oryzae*^10^.

On the other hand, the introduction of the glucose moiety, due to its hydrophilicity, positively affects the aqueous solubility of flavonoids, which is a hly important factor for its bioavailability^28,29^. In nature, most flavonoids, except catechins, are present as *β*-glycosides. In general, glucosides are the only glycosides that can be absorbed from the small intestine and then reach her plasma levels than compounds absorbed mostly in the colon^30,31^. Therefore, glycosylation is a common strategy to improve chemical stability, water solubility, bioavailability, and pharmacological potency of flavonoids. Enzymes called glycosyltransferases (GTs) are mainly responsible for glycosylation in living organisms and have been found in plants, animals, bacteria, and fungi ^32–37^. Regarding the glycosylation of chalcones, studies using UDP-glycosyltransferase (YjiC) from *Bacillus licheniformis* DSM-13 have been reported. Li et al. obtained derivatives of isobavachalcone with glucosyl moiety at C-4 in ring B and C-4′ in ring A^36^. On the other hand, the reaction of YjiC with UDP-glucose and phloretin led to the formation of five different types of phloretin glucosides (with glucose moiety at C-2′, at C-4′, at C-2′, and C-4′, at C-4 and C-6′, and at C-4 and C-4′)^35^. The promising flavonoid glycosylation strategy can also be whole-cell biotransformation with entomopathogenic filamentous fungi as biocatalysts^38–42^. Fungal glucosyltransferases remain poorly known. However, enzymes such as UGT59A1 from *Absidia caerulea* and UGT58A1 from *Rhizopus japonicus*^43^, and glycosyltransferase-methyltransferase (GT-MT) functional module BbGT86-BbMT85 from *Beauveria bassiana* have been characterized^44^. Moreover, Xie et al. used a combination of genome mining and heterologous expression techniques to identify glycosyltransferase-methyltransferase (GT-MT) functional modules from other Hypocreales fungi such as *Isaria fumosorosea*, *Claviceps purpurea*, *Cordyceps militaris*, and *Metarhizium robertsii*. These GT-MT modules possess decent substrate promiscuity and regiospecificity, and can methylglucosylate flavonoid compounds, among others.

Having regard to the above, the main aim of the presented work was to obtain 2-chloro-2′-hydroxychalcone and 3-chloro-2′-hydroxychalcone and then biotransform them in cultures of entomopathogenic filamentous fungi to receive their glycosylated derivatives. We carried out microbial transformation in two strains of fungi, i.e., *I. fumosorosea* KCH J2 and *B. bassiana* KCH J1.5, resulting in the formation of 2-chlorodihydrochalcone 2′-*O*-*β*-d-(4″-*O*-methyl)-glucopyranoside, 3-chlorodihydrochalcone 2′-*O*-*β*-d-(4″-*O*-methyl)-glucopyranoside and some other glycosylated derivatives. All of these biotransformation products have not been previously described in the literature. Moreover, we employed computer-aided simulations to evaluate and compare the physicochemical properties, pharmacokinetics, and potential biological activity of all of the obtained compounds. The flavonoid aglycones (2-chloro-2′-hydroxychalcone and 3-chloro-2′-hydroxychalcone) and their glycosides obtained in the fungal biotransformation process, and also 2′-hydroxychalcone were screened for antimicrobial activity. The use of 2′-hydroxychalcone, which does not have additional substituents except 2′-hydroxyl group, made it possible to observe the influence of the presence of a chlorine atom in the structure of the tested compounds on their antimicrobial activity.

## Results and discussion

### Synthesis of 2-chloro-2′-hydroxychalcone (3) and 3-chloro-2′-hydroxychalcone (5)

**Figure 1:**
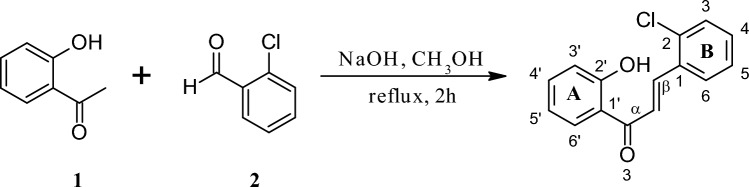
Chemical synthesis of 2-chloro-2′-hydroxychalcone (**3**) by the Claisen-Schmidt condensation reaction.

**Figure 2:**
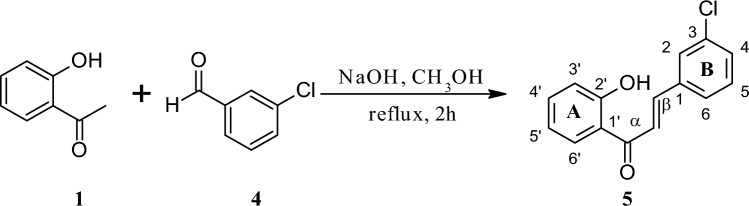
Chemical synthesis of 3-chloro-2′-hydroxychalcone (**5**) by the Claisen-Schmidt condensation reaction.

**Figure 3:**
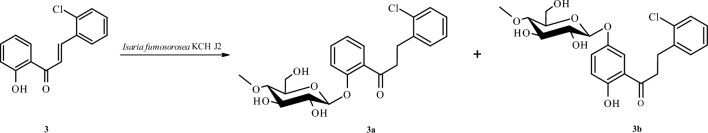
Microbial transformation of 2-chloro-2′-hydroxychalcone (**3**) in *I. fumosorosea* KCH J2 culture.

In the first stage of the study, as a result of the Claisen-Schmidt condensation reactions, two flavonoids possessing in their structures chlorine atom and 2′-hydroxyl group, i.e., 2-chloro-2′-hydroxychalcone (Fig. 1) and 3-chloro-2′-hydroxychalone (Fig. 2) were obtained.

The structures of both synthesis products (**3** and** 5**) were confirmed based on NMR (Nuclear Magnetic Resonance) spectroscopy (Tables 1 and 2 in section “Materials and methods”). In the ^1^H-NMR (Proton Nuclear Magnetic Resonance) spectrum of 2-chloro-2′-hydroxychalcone (**3**), a characteristic signal from the proton of the hydroxyl moiety at *δ* = 12.74 ppm with the corresponding signal from C-2′ in the ^13^C-NMR (Carbon-13 Nuclear Magnetic Resonance) spectrum at *δ* = 164.5 ppm (Supplementary Information: Figs. S3 and S5) was observed. In the case of 3-chloro-2′-hydroxychalcone (**5**), this signal was observed at *δ* = 12.79 ppm in the ^1^H-NMR spectrum with the corresponding signal at *δ* = 164.6 ppm in the ^13^C-NMR spectrum (Supplementary Information: Figs. S21 and S23). In the ^1^H-NMR spectra of compounds **3** and **5** were also observed characteristic two doublets from H-α (*δ* = 8.10 ppm in **3** and H-*δ* = 8.16 ppm in **5**) and β (*δ* = 8.32 ppm in **3** and 7.91 ppm in **5**) (Supplementary Information: Figs. S4 and S22). In the ^13^C-NMR spectra, the signal from the carbonyl group was observed at *δ* = 194.8 ppm in **3** and at *δ* = 194.9 ppm in **5** (Supplementary Information: Fig. S5 and S23). The substitution with a chlorine atom in the structure of 2′-hydroxychalcone in position C-2 in ring B (product **3**) confirmed the arrangement of other proton signals in ring B (from H-3 doublet of doublets at *δ* = 7.56 ppm, from H-4 triplet of doublets at *δ* = 7.50 ppm, from H-5 multiplet at *δ* = 7.45 ppm, and from H-6 doublet of doublets at *δ* = 8.17 ppm) (Supplementary Information: Fig. S4). Moreover, the signal from H-α (*δ* = 8.10 ppm) correlated with the shifted to the lower field signal from C-1 (*δ* = 133.6 ppm) in the HMBC (Heteronuclear Multiple Bond Correlation) experiment (Supplementary Information: Fig. S12). On the other hand, in the ^1^H-NMR spectrum of **5**, a characteristic isolated singlet from H-2 at *δ* = 8.01 ppm confirmed substitution with a chlorine atom in its direct neighbourhood at C-3 (Supplementary Information: Fig. S22). Moreover, the signal from H-2 (*δ* = 8.01 ppm) correlated with the shifted to the lower field signal from C-3 (*δ* = 135.4 ppm) in the HMBC experiment (Supplementary Information: Fig. S30). The molecular masses of both synthesis products with chlorine atoms (**3** and **5**) were also confirmed by LC–MS spectroscopy (Liquid Chromatography‐Mass Spectrometry) (Supplementary Information: Fig. S1 and S19, respectively).

**Table 1:** ^1^H-NMR chemical shifts δ (ppm) and coupling constants *J* (Hz) of 2-chloro-2′-hydroxychalcone (**3**) and products of its biotransformation **3a–3c** in Acetone-d6, 600 MHz (Supplementary Information: Figs. S3–S4, S39–S41, S64–S66, S90–S92).

Proton	Compound			
	**3**	**3a**	**3b**	**3c**
H-α	8.10 (d) *J* = 15.5	3.46 (m)	3.45 (m)	3.44 (m)
H-β	8.32 (d) *J* = 15.5	3.10 (t) *J* = 7.5	3.16 (m)	3.14 (m)
H-3	7.56 (dd) *J* = 8.0, *J* = 1.3	7.37 (dd) *J* = 7.9, *J* = 1.3	7.41 (dd) *J* = 7.6, *J* = 1.7	–
H-4	7.50 (td) *J* = 7.6, *J* = 1.7	7.21 (td) *J* = 7.6, *J* = 1.7	7.24 (m)	6.89 (m)
H-5	7.45 (m)	7.25 (td) *J* = 7.5, *J* = 1.4	7.30 (m)	7.09 (t) *J* = 7.8
H-6	8.17 (dd) *J* = 7.8, *J* = 1.7	7.40 (dd) *J* = 7.6, *J* = 1.6	7.45 (dd) *J* = 7.1, *J* = 2.1	6.89 (m)
H-3′	7.02 (m)	7.31 (dd) *J* = 8.4 *J* = 0.5	6.88 (d) *J* = 9.0	–
H-4′	7.60 (ddd) *J* = 8.2, *J* = 7.3, *J* = 1.6	7.49 (ddd) *J* = 8.5, *J* = 7.3, *J* = 1.8	7.30 (m)	7.40 (dd) *J* = 8.0, *J* = 1.4
H-5′	7.02 (m)	7.11 (td) *J* = 7.6, *J* = 0.9	–	6.89 (m)
H-6′	8.29 (dd) *J* = 8.5, *J* = 1.5	7.60 (dd) *J* = 7.7, *J* = 1.8	7.66 (d) *J* = 2.9	7.66 (dd) *J* = 8.2, *J* = 1.4
H-1”	–	5.06 (d) *J* = 7.8	4.85 (d) *J* = 7.8	4.91 (d) *J* = 7.7
H-2”	–	3.53 (m)	3.45 (m)	3.50 (t) *J* = 8.6,
H-3”	–	3.64 (t) *J* = 9.1	3.58 (m)	3.62 (m)
H-4”	–	3.20 (dd) *J* = 9.6, *J* = 9.1	3.16 (m)	3.22 (m)
H-5”	–	3.50 (ddd) *J* = 9.7, *J* = 5.1, *J* = 2.1	3.45 (m)	3.44 (m)
H-6”	–	3.83 (d) *J* = 11.4 3.68 (d) *J* = 10.4	3.82 (m) 3.82 (m)	3.80 (m) 3.68 (m)
4″-OCH_3_	–	3.55 (s)	3.54 (s)	3.56 (s)
C2′-OH	12.74 (s)	–	11.89 (s)	12.07 (s)
3-OH	–	–	–	8.70
2″-OH	–	4.67 (d) *J* = 3.2	4.66 (d) *J* = 4.0	4.69 (d) *J* = 1.9
3″-OH	–	4.56 (s)	4.42 (d) *J* = 4.1	4.43 (d) *J* = 4.0
6″-OH	–	–	3.67 (ddd) *J* = 13.3, *J* = 6.2, *J* = 2.3	3.80 (m)

**Table 2:** ^13^C-NMR chemical shifts δ (ppm) of 2-chloro-2′-hydroxychalcone (**3**) and products of its biotransformation **3a–3c** in Acetone-d6, 151 MHz (Supplementary Information: Figs. S5–S6, S42–S44, S67–S70, S93–S95).

Carbon	Compound			
	**3**	**3a**	**3b**	**3c**
C-α	124.2	43.6	38.5	39.6
C-β	141.0	28.6	28.3	28.9
C-1	133.6	140.0	139.3	140.8
C-2	136.1	134.4	134.4	121.1
C-3	131.1	130.1	130.2	154.2
C-4	133.0	128.5	128.8	115.4
C-5	128.5	128.0	128.1	128.2
C-6	129.4	131.5	131.6	122.1
C-1′	120.8	130.5	119.8	121.5
C-2′	164.5	157.2	158.5	153.4
C-3′	119.0	117.3	119.5	147.2
C-4′	137.7	134.1	127.6	123.8
C-5′	120.0	123.1	150.8	119.4
C-6′	131.6	130.4	118.2	124.6
C-1″	–	102.3	102.8	102.5
C-2″	–	75.0	74.9	74.9
C-3″	–	78.1	78.0	77.8
C-4″	–	80.1	80.3	80.0
C-5″	–	77.2	77.1	77.1
C-6″	–	62.1	62.2	62.0
4″-OCH_3_	–	60.6	60.6	60.6
C = O	194.8	201.9	206.0	206.1

The application of entomopathogenic filamentous fungi as biocatalysts in the microbial transformation of 2-chloro-2′-hydroxychalcone (**3**) and 3-chloro-2′-hydroxychalcone (**5**) in cultures of fungi strains *I. fumosorosea* KCH J2 and *B. bassiana* KCH J1.5 resulted in the formation of six new glycosylated flavonoids. The products of the biotransformations were isolated from the post-reaction mixture and purified using preparative thin-layer chromatography (TLC). The yields of biotransformations were determined based on the isolated amounts of the products. Their chemical structures were established based on the NMR spectroscopy and confirmed by LC–MS. Their biological activity was assessed using computational methods based on the structure–activity relationship. Moreover, the antimicrobial activity of biotransformation substrates (**3** and **5**) and their main biotransformation products (**3a**, **3b**, **5a**) together with 2′-hydroxychalcone were screened in the experiments with the selected microbial strains using automatic measurements of their growth.

### Biotransformation of 2-chloro-2′-hydroxychalcone (3) in the culture of *I. fumosorosea* KCH J2

2-chloro-2′-hydroxychalcone (**3**) was biotransformed in the culture of *I. fumosorosea* KCH J2 into 2-chlorodihydrochalcone 2′-*O*-*β*-d-(4″-*O*-methyl)-glucopyranoside (**3a**) with a yield of 74.5% (62.9 mg) and 2-chloro-2′-hydroxydihydrochalcone 5′-*O*-*β*-d-(4″-*O*-methyl)-glucopyranoside (**3b**) with a yield of 11.0% (9.6 mg) (Fig. 3).

**Figure 4:**
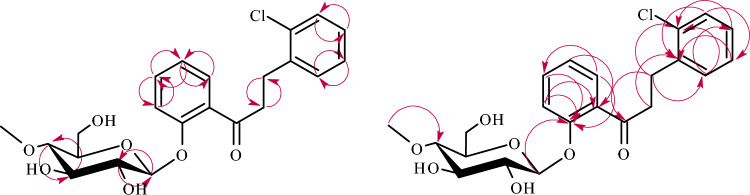
Key COSY (on the left) and HMBC (on the right) correlations for the structure elucidation of product **3a**.

**Figure 5:**
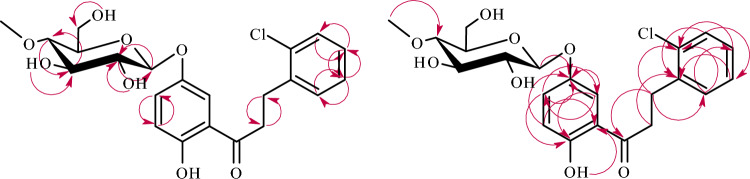
Key COSY (on the left) and HMBC (on the right) correlations for the structure elucidation of product **3b**.

The structures of products **3a**–**3b** were determined based on NMR spectroscopy (Tables 1 and 2 in section “Materials and methods”, Figs. 4 and 5 below showing key COSY (Correlation Spectroscopy) and HMBC correlations in section “Materials and methods”). Their molecular masses were confirmed using LC–MS (Section “Materials and methods” and Supplementary Information: Figs. S37 and S62).

The presence of a glucose moiety in the biotransformation product **3a** was confirmed by five characteristic carbon signals observed in the region from *δ* = 80.1 ppm to *δ* = 62.1 ppm in the ^13^C-NMR spectrum (Supplementary Information: Fig. S44), as well as proton signals of *δ*_H_ ranging from *δ* = 3.83 ppm to *δ* = 3.20 ppm in the ^1^H-NMR spectrum (Supplementary Information: Fig. S41). In the ^1^H-NMR spectrum, a one-proton doublet from the proton at the anomeric carbon atom was present at *δ* = 5.06 ppm with the coupling constant (*J* = 7.8 Hz) evidencing *β*-configuration of the glucose (Supplementary Information: Fig. S41). The glucose molecule was also *O*-methylated at C-4″ because, in the ^1^H-NMR spectrum, a three-proton singlet at *δ* = 3.55 ppm, with the corresponding signal at *δ* = 60.6 ppm in the ^13^C-NMR spectra was observed (Supplementary Information: Figs. S41 and S44). Moreover, this moiety was correlated with the signal of C-4″ (*δ* = 80.1 ppm) in the HMBC experiment, which evidences the substitution position with the –O–CH_3_ group in the glucose unit (Supplementary Information: Fig. S55). In product **3a**, the reduction of a double bond between C-α and C-β occurred and caused the shift of the protons at C-α from *δ* = 8.10 ppm (in **3**) to *δ* = 3.46 ppm (in **3a**) and the protons at C-β from *δ* = 8.32 ppm (in **3**) to *δ* = 3.10 ppm (in **3a**) (Supplementary Information: Figs. S4 and S41). Naturally, shifted protons at C-α and C-β were correlated with the C-1 signal and the carbonyl group in the HMBC experiment (Supplementary Information: Figs. S54 and S56). The signal from one proton of the hydroxyl group at C-2′ that was present in the ^1^H-NMR spectrum of the biotransformation substrate **3** at *δ* = 12.74 ppm was absent in the ^1^H-NMR of the biotransformation product **3a** (Supplementary Information: Fig. S3 and S39), which pointed at substitution at C-2′ with the 4″-*O*-methylglucopyranose. This was also proved in the HMBC experiment, in which the signal from H-1″ at *δ* = 5.06 ppm correlated with the shifted signal from C-2′ at *δ* = 157.2 ppm. The signal assigned as C-2′ also correlated with H-6′ at *δ* = 7.60 ppm, H-4′ at *δ* = 7.49 ppm, and H-3′ at *δ* = 7.31 ppm, which proved undoubtedly that the signal at *δ* = 157.2 ppm came from C-2′ (Supplementary Information: Figs. S54 and S52).

In product **3b** the 4″-*O*-methylglucosyl moiety was also attached to the flavonoid aglycone in the *β*-configuration (Supplementary Information: Figs. S66 and S70). In this biotransformation product also occurred the reduction of a double bond between C-α and C-β (Supplementary Information: Figs. S66 and S70). The signal from one proton of the hydroxyl group at C-2′ in the ^1^H-NMR spectrum was shifted from *δ* = 12.74 ppm (in biotransformation substrate **3)** to *δ* = 11.89 ppm (in biotransformation product **3b**), which indicated that the substitution with the glucosyl moiety occurred in ring A of the flavonoid compound (Supplementary Information: Figs. S3 and S64). In the ^1^H-NMR spectrum of **3b** the shifts of the signals of the other protons from ring A were observed: H-6′ (*δ* = 8.29 ppm in substrate **3** and *δ* = 7.66 ppm in product **3b**), H-4′ (*δ* = 7.60 ppm in substrate **3** and *δ* = 7.30 ppm in product **3b**) and H-3′ (*δ* = 7.02 ppm in substrate **3** and *δ* = 6.88 ppm in product **3b**) (Supplementary Information: Figs. S4 and S65). The absence of the signal from H-5′ in the neighborhood of H-3′ (visible in substrate **3**) was also evident, which indicated substitution with the glucosyl moiety at C-5′ (Supplementary Information: Figs. S4 and S65). In the HMBC experiment, the signal from H-1″ at *δ* = 4.85 ppm correlated with the shifted signal at *δ* = 150.8 ppm, which was assigned as C-5′ (Supplementary Information: Fig. S80). Moreover, the signal at *δ* = 150.8 ppm correlated with H-6′ at *δ* = 7.66 ppm, H-4′ at *δ* = 7.30 ppm, and H-3′ at *δ* = 6.88 ppm, which proved that it came from C-5′ (Supplementary Information: Fig. S79).

### Biotransformation of 2-chloro-2′-hydroxychalcone (3) in the culture of *B. bassiana* KCH J1.5

**Figure 6:**
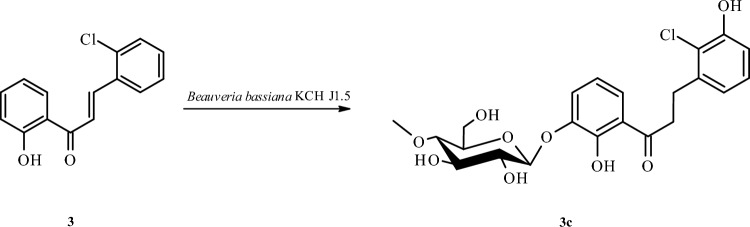
Microbial transformation of 2-chloro-2′-hydroxychalcone (**3**) in *B. bassiana* KCH J1.5 culture.

2-Chloro-2′-hydroxychalcone (**3**) was not so efficiently biotransformed in the culture of *B. bassiana* KCH J1.5 as in the case of *I. fumosorosea* KCH J2. The main biotransformation product, i.e., 2-chloro-2′,3-dihydroxydihydrochalcone 3′-*O*-*β*-d-(4″-*O*-methyl)-glucopyranoside (**3c**) was isolated only with a yield of 12.8% (11.6 mg) (Fig. 6).

**Figure 7:**
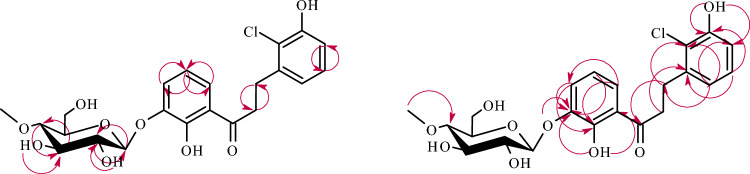
Key COSY (on the left) and HMBC (on the right) correlations for the structure elucidation of product **3c**.

The structure of product **3c** was also determined based on NMR spectroscopy (Tables 1 and 2 in section “Materials and methods”, Fig. 7 below showing key COSY and HMBC correlations). Its molecular mass was confirmed using LC–MS (section “Materials and methods” and Supplementary Information: Fig. S88).

This biotransformation product was a result of a few chemical reactions resulting in the glycosylated and hydroxylated derivative. Firstly, compound **3c** was identified as dihydrochalcone because the reduction of a double bond between C-α and C-β occurred causing chemical shifts in the location of the signal of H-α (from *δ* = 8.10 ppm in **3** to *δ* = 3.44 ppm in **3c**) and the signal of H-β (from *δ* = 8.32 ppm in **3** to *δ* = 3.14 ppm in **3c**) in the ^1^H-NMR spectrum (Supplementary Information: Figs. S4 and S92). Secondly, the signal from one proton of the hydroxyl group at C-2′ was shifted from *δ* = 12.74 ppm (in biotransformation substrate **3)** to *δ* = 12.07 ppm (in biotransformation product **3c**), which indicated the substitution in ring A of the product **3c** (Supplementary Information: Figs. S3 and S90). This substituent was a glucose moiety because in the ^1^H-NMR and ^13^C-NMR spectra were observed characteristic signals, similar to the above mentioned for product **3a** (Supplementary Information: Figs. S92 and S95). It was attached at C-3′ because it’s signal (*δ* = 147.2 ppm), which correlated with H-1″ from the glucosyl moiety (*δ* = 4.91 ppm) in the HMBC experiment, correlated also with 2′-OH (*δ* = 12.07 ppm), H-4′ (*δ* = 7.40 ppm), and H-5′ (*δ* = 6.89 ppm) (Supplementary Information: Figs. S106, S103, and S104). Thirdly, another signal from the proton of the hydroxyl group at *δ* = 8.70 ppm was observed. Along with the observed shifts in the position of protons coming from the B ring of product **3c**, it indicated substitution in ring B. The hydroxyl group was attached at C-3 which resulted in the shift of H-5 (from *δ* = 7.45 ppm in **3** to *δ* = 7.09 ppm in **3c**) H-4 (from *δ* = 7.50 ppm in **3** to *δ* = 6.89 ppm in **3c**) and H-6 (from *δ* = 8.17 ppm in **3** to *δ* = 6.89 ppm in **3c**) (Supplementary Information: Figs. S90 and S91). Moreover, in the HMBC experiment, the proton of the hydroxyl group at *δ* = 8.70 ppm (assigned as 3-OH) correlated with C-4 (*δ* = 115.4 ppm) and C-6 (*δ* = 122.1 ppm) (Supplementary Information: Fig. S104). Furthermore, protons H-5 (*δ* = 7.09 ppm) and H-4 (*δ* = 6.89 ppm) correlated also with C-3 (*δ* = 154.2 ppm) (Supplementary Information: Fig. S104).

### Biotransformation of 3-chloro-2′-hydroxychalcone (5) in the culture of *I. fumosorosea* KCH J2

**Figure 8:**
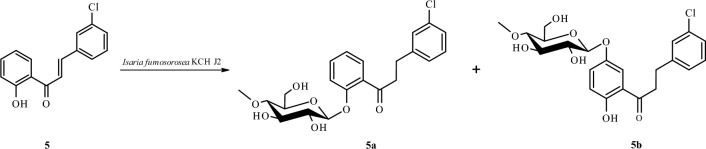
Microbial transformation of 3-chloro-2′-hydroxychalcone (**5**) in *I. fumosorosea* KCH J2 culture.

The second product of chemical synthesis, i.e., 3-chloro-2′-hydroxychalcone (**5**) was biotransformed in the culture of *I. fumosorosea* KCH J2 into 3-chlorodihydrochalcone 2′-*O*-*β*-d-(4″-*O*-methyl)-glucopyranoside (**5a**) with a yield of 41% (34.6 mg) and 3-chloro-2′-hydroxydihydrochalcone 5′-*O*-*β*-d-(4″-*O*-methyl)-glucopyranoside (**5b**) with a yield of 13.8% (12.1 mg) (Fig. 8).

**Figure 9:**
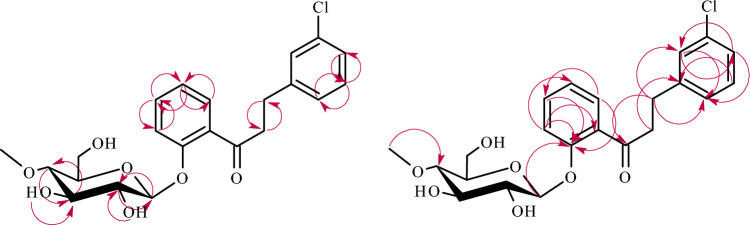
Key COSY (on the left) and HMBC (on the right) correlations for the structure elucidation of product **5a**.

**Figure 10:**
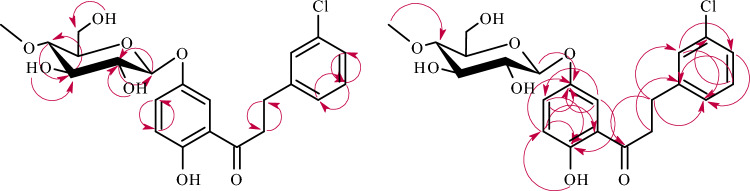
Key COSY (on the left) and HMBC (on the right) correlations for the structure elucidation of product **5b**.

The structures of products **5a**–**5b** were determined based on NMR spectroscopy (Tables 3 and 4 in section “Materials and methods”, Figs. 9 and 10 below showing key COSY and HMBC correlations). Their molecular masses were confirmed using LC–MS (section “Materials and methods” and Supplementary Information: Figs. S113 and S138).

**Table 3:** ^1^H-NMR chemical shifts δ (ppm) and coupling constants *J* (Hz) of 3-chloro-2′-hydroxychalcone (**5**) and products of its biotransformation **5a–5c** in Acetone-d6, 600 MHz (Supplementary Information: Figs. S21–S22, S115-S117, S140-S142, S165-S167).

Proton	Compound			
	5 (1138)	5a (1157)	5b (1197)	5c (32B)
H-α	8.16 (d) *J* = 15.5	3.46 (m)	3.51 (m)	3.47 (m)
H-β	7.91 (d) *J* = 15.5	2.99 (t) *J* = 7.4	3.06 (t) *J* = 7.4	3.02 (t) *J* = 7.6
H-2	8.01 (s)	7.33 (m)	7.39 (s)	7.26 (d) *J* = 1.9
H-4	7.51 (m)	7.19 (m)	7.23 (m)	–
H-5	7.51 (m)	7.28 (t) *J* = 7.6	7.29 (m)	7.30 (d) *J* = 8.1
H-6	7.83 (m)	7.25 (m)	7.29 (m)	6.96 (m)
H-3′	7.01 (m)	7.31 (d) *J* = 8.3	6.87 (d) *J* = 9.0	6.96 (m)
H-4′	7.59 (m)	7.48 (ddd) *J* = 8.9 *J* = 7.4 *J* = 1.8	7.29 (m)	7.53 (m)
H-5′	7.01 (m)	7.10 (m)	–	6.96 (m)
H-6′	8.32 (m)	7.59 (dd) *J* = 7.7, *J* = 1.7	7.67 (d) *J* = 2.9	8.00 (dd) *J* = 8.4, *J* = 1.6
H-1”	–	5.08 (d) *J* = 7.8	4.85 (d) *J* = 7.8	5.05 (d) *J* = 7.6
H-2”	–	3.52 (m)	3.45 (m)	3.51(m)
H-3”	–	3.64 (td) *J* = 9.0, *J* = 3.8	3.58 (m)	3.61 (m)
H-4”	–	3.22 (m)	3.14 (m)	3.20 (dd) *J* = 9.6, *J* = 8.8
H-5”	–	3.52 (m)	3.45 (m)	3.51 (m)
H-6”	–	3.84 (ddd) *J* = 11.7, *J* = 5.1 *J* = 1.9 3.70 (m)	3.85 (m) 3.85 (m)	3.85 (m) 3.68 (m)
4″-OCH_3_	–	3.56 (s)	3.54 (s)	3.55 (s)
C2′-OH	12.79 (s)	-	11.88 (s)	12.25 (s)
2″-OH	–	4.68 (d) *J* = 4.1	4.66 (d) *J* = 3.9	4.60 (d) *J* = 4.3
3″-OH	–	4.53 (d) *J* = 4.1	4.43 (d) *J* = 4.1	4.45 (d) *J* = 4.1
6″-OH	–	3.77 (m)	3.67 (dt) *J* = 11.2, *J* = 5.8	3.81 (dd) *J* = 5.1, *J* = 2.6

**Table 4:** ^13^C-NMR chemical shifts δ (ppm) of 3-chloro-2′-hydroxychalcone (**5**) and products of its biotransformation **5a–5c** in Acetone-d6, 151 MHz (Supplementary Information: Figs. S23–S24, S118–S120, S143–S145, S168–S170).

Carbon	Compound			
	**5** (1138)	**5a** (1157)	**5b** (1197)	**5c** (32B)
C-α	123.1	45.3	39.9	40.2
C-β	144.4	30.5	29.7	30.0
C-1	137.9	145.3	144.6	142.5
C-2	129.1	129.4	129.4	117.4
C-3	135.4	134.4	134.5	121.0
C-4	131.6	126.7	126.9	153.8
C-5	131.5	130.8	130.8	130.6
C-6	128.7	127.9	128.0	123.7
C-1′	120.8	130.4	119.8	120.2
C-2′	164.6	157.2	158.4	163.1
C-3′	119.0	117.2	119.4	118.8
C-4′	137.7	134.1	127.5	137.3
C-5′	119.9	123.1	150.8	119.9
C-6′	131.4	130.4	118.2	131.6
C-1″	–	102.2	102.8	101.3
C-2″	–	75.0	74.9	74.8
C-3″	–	78.1	78.0	78.2
C-4″	–	80.1	80.4	80.1
C-5″	–	77.3	77.2	77.1
C-6″	–	62.1	62.3	62.1
4″-OCH_3_	–	60.6	60.6	60.6
C = O	194.9	201.8	206.4	207.0

The product **5a** was analogous to product **3a** (the only difference between them is the position of substitution with the chlorine atom). In this case also occurred the attachment of 4″-*O*-methylglucosyl moiety in *β*-configuration at C-2′ because in the ^1^H-NMR spectrum, the signal from 2′-OH disappeared and characteristic signals from the glucose moiety were observed (Supplementary Information: Figs. S115, S117, and S120). Moreover, in **5a**, similarly to **3a**, there was a reduction of a double bond between C-α and C-β, causing characteristic chemical shifts in the ^1^H-NMR spectrum (Supplementary Information: Figs. S22, S24, S117, and 120).

The compound **5b** was analogous to product **3b** (the only difference between them is the position of substitution with the chlorine atom). In this biotransformation product, the 4″-*O*-methylglucosyl moiety was attached to the flavonoid aglycone in the *β*-configuration (Supplementary Information: Figs. S142 and S145). The reduction of a double bond between C-α and C-β was confirmed by ^1^H-NMR and ^13^C-NMR spectra (Supplementary Information: Figs. S142 and S145). The signal from one proton of the hydroxyl group at C-2′ in the ^1^H-NMR spectrum was shifted from *δ* = 12.79 ppm (in biotransformation substrate **5)** to *δ* = 11.88 ppm (in biotransformation product **5b**), which indicated that the substitution with the glucosyl moiety occurred in ring A of the flavonoid compound (Supplementary Information: Figs. S21 and S140). Similarly, to **3b**, the absence of the signal from H-5′ in the neighborhood of H-3′ (visible in substrate **5**) indicated substitution with the glucosyl moiety at C-5′ (Supplementary Information: Figs. S22 and S141). Furthermore, in the HMBC experiment, the signals from H-6′ at *δ* = 7.67 ppm, H-4′ at *δ* = 7.29 ppm, H-3′ at *δ* = 6.87 ppm, and H-1″ at *δ* = 4.85 ppm correlated with the shifted signal at *δ* = 150.8 ppm (assigned as C-5′), which proved substitution at C-5′ (Supplementary Information: Figs. S155 and S156).

### Biotransformation of 3-chloro-2′-hydroxychalcone (5) in the culture of *B. bassiana* KCH J1.5

**Figure 11:**
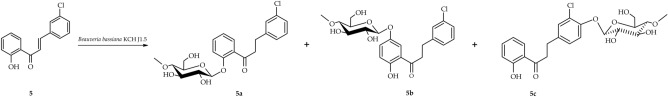
Microbial transformation of 3-chloro-2′-hydroxychalcone (**5**) in *B. bassiana* KCH J1.5 culture.

**Figure 12:**
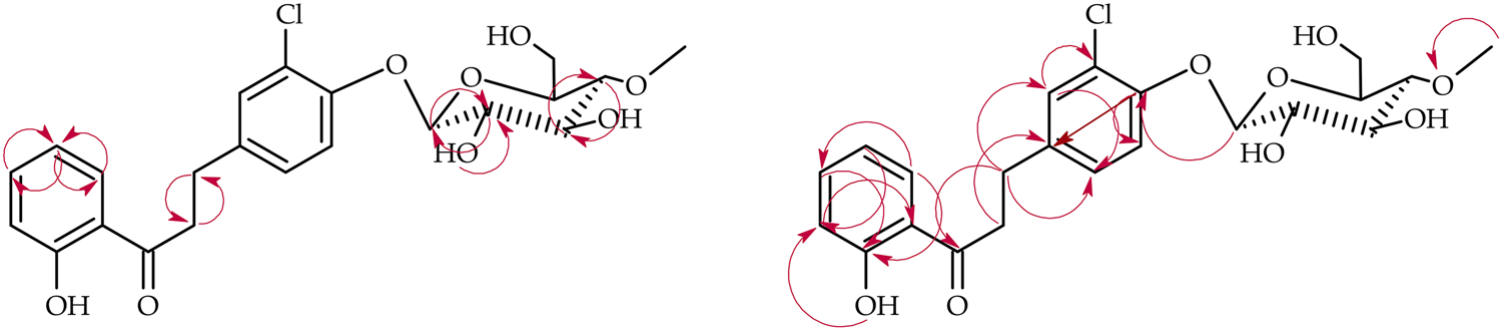
Key COSY (on the left) and HMBC (on the right) correlations for the structure elucidation of product **5c**.

Biotransformation of 3-chloro-2′-hydroxychalcone (**5**) in the culture of *B. bassiana* KCH J1.5 resulted in the formation of three products, i.e., 3-chlorodihydrochalcone 2′-*O*-*β*-d-(4″-*O*-methyl)-glucopyranoside (**5a**) with a yield of 14.7% (12.4 mg), 3-chloro-2′-hydroxydihydrochalcone 5′-*O*-*β*-d-(4″-*O*-methyl)-glucopyranoside (**5b**) with a yield of 10.3% (9.0 mg), and 3-chloro-2′-hydroxydihydrochalcone 4-*O*-*β*-d-(4″-*O*-methyl)-glucopyranoside (**5c**) with a yield of 6.6% (5.8 mg) (Fig. 11). The first two listed, based on the ^1^H-NMR analysis, were identified as the same products (**5a** and **5b**) as obtained in the biotransformation in the culture of *I. fumosorosea* KCH J2. The structure of the third, unknown product **5c** was determined based on NMR spectroscopy (Tables 3 and 4 in section “Materials and methods”, Fig. 12 below showing key COSY and HMBC correlations). Its molecular mass was confirmed using LC–MS (section “Materials and methods” and Supplementary Information: Fig. S163).

The last biotransformation product **5c** was glycosylated because the characteristic signals of 4″-*O*-methylglucosyl moiety were present in the ^1^H-NMR and ^13^C-NMR spectra (Supplementary Information: Figs. S167 and S170). In this product occurred also the reduction of a double bond between C-α and C-β (Supplementary Information: Figs. S167 and S170). The signal from one proton of the hydroxyl group at C-2′, similar to the other signals of protons of ring A, was only slightly shifted, compared to substrate **5**, because of mentioned hydrogenation of the double bond between C-α and C-β (Supplementary Information: Figs. S21, S22, S165, and 166). The substitution with the glucosyl moiety occurred in ring B at C-4. The signal from the H-2 proton in the ^1^H NMR spectrum of product **5c** is a doublet with a coupling constant of *J* = 1.9, which indicates that only one meta hydrogen (H-6) is in its vicinity. Such an arrangement is possible if the glycosidic unit was attached to the C-4. The ^1^H-NMR spectrum also shows a doublet (δ = 7.30) with a coupling constant of *J* = 8.1, which indicates that there is a proton in the ortho position in relation to it (H-5). The COSY spectrum also confirms the position of the substitution by the presence of coupling of the analyzed signal with the signal originating from the H-6 proton (Supplementary Information: Fig. S180).

Both biotransformation substrates, i.e. 2-chloro-2′-hydroxychalcone (**3**) and 3-chloro-2′-hydroxychalcone (**5**) were biotransformed in cultures of fungi strains *I. fumosorosea* KCH J2 and *B. bassiana* KCH J1.5 into glycosylated dihydrochalcones. The main products were obtained by the attachment of 4″-*O*-methylglucosyl moiety to the hydroxyl group at C-2′ in the A ring. In our previous works on methylchalcones^42,45^, products of glycosylation of the 2′-hydroxyl group were not so common, which may indicate the influence of the chlorine substituent in the B ring on the glycosylation site. The results of the present study showed differences in the action of glycosyltransferase‐methyltransferase functional modules of both used strains. *I fumosorosea* KCH J2 enzymatic system was able to effectively attach glucose moiety to both substrates at the same positions: C-2′ and C-5′. On the other hand, biotransformations in the culture of *B. bassiana* KCH J1.5 were not such successful and regioselective because led to the formation of a small amount of 2-chloro-2′,3-dihydroxydihydrochalcone 3′-*O*-*β*-d-(4″-*O*-methyl)-glucopyranoside (**3c**) from 2-chloro-2′-hydroxychalcone and as much as three derivatives with 4″-O-methyglycoside at C-2, C-5′, and C-5 from 3-chloro-2′-hydroxychalcone.

Previous studies on glycosylation of chalcones by filamentous fungi involved the formation of 4′-*O*-*β*-d-glucopyranoside derivatives of 2′,4,4′-trihydroxy-6′-methoxy-3′-(3-methylbut-2-en-1-yl)chalcone called xanthohumol by *Penicillium chrysogenum* 6933^46^, *Absidia coerulea* AM93 and *Rhizopus nigricans* UPF701^47^ or 4’-*O*-*β*-d-(4″-*O*-methyl)-glucopyranoside derivatives of xanthohumol by *B. bassiana* AM278^48^ and *B. bassiana* AM446^47^. On the other hand, UDP-glycosyltransferase (YjiC) from *Bacillus licheniformis* DSM-13 transformed phloretin into five glucosides (with glucose moiety at C-2′, at C-4′, at C-2′, and C-4′, at C-4 and C-6′, and at C-4 and C-4′)^35^. However, effective glycosylation of chlorochalcones was previously described only by our team ^49^. Unfortunately, it is difficult to draw a universal conclusion on the impact of glycosylation on the biological activity of flavonoids. Some studies showed a reduction of. e.g. anti-oxidant, anti-inflammatory, antibacterial activity, and others described an increase in, e.g. tyrosinase inhibition, antivirus, and antiallergic activity. The effects of flavonoid glycosylation may likely differ in in vitro and in vivo studies^29^.

### Pharmacokinetics, drug-likeness, and biological activity prediction

#### 2-Chloro-2′-hydroxychalcone (3), 3-chloro-2′-hydroxychalcone (5) and their derivatives (3a-3c, 5a-5c) SwissADME

Pharmacokinetics, aqueous solubility, and drug-likeness of 2-chloro-2′-hydroxychalcone (**3**) and its biotransformation products (**3a**-**3c**) as well as 3-chloro-2′-hydroxychalcone (**5**) and its biotransformation products (**5a**-**5c**), and for comparison 2′-hydroxychalcone (**6**) have been predicted using the SwissADME website (screens of predictions are shown in Supplementary Information: Figs. S14, S32, S57, S83, S108, S133, S158, S183, and S188) developed and managed by the Molecular Modeling Group of the Swiss Institute of Bioinformatics (SIB)^50^. Brain Or IntestinaL EstimateD permeation method (BOILED-Egg), as a predictive model computing the lipophilicity and polarity of small molecules^51^, indicated h gastrointestinal absorption of all tested molecules except 2-chloro-2’,3-dihydroxydihydrochalcone 3′-*O*-*β*-d-(4″-*O*-methyl)-glucopyranoside (**3c**). The water solubility prediction based on ESOL—Estimated SOLubility method of the glycosylated derivatives (**3a-3c**, **5a-5c**) was 12 to 22 times her than their aglycone forms (**3** and **5**). At the same time, their estimated lipophilicity—consensus Log Po/w decreased, which may negatively impact affinity for biological membranes and the passive permeation in the bloodstream^50^. An increase in the hydrophilicity of the compounds limits their incorporation into the hydrophobic interior of the membrane, but as our previous studies have shown, the methylflavanone 4″-*O*-methylglucosides were able to bind in the hydrophilic region of the membranes (phosphatidylcholine membrane and membrane of erythrocytes). In addition, they bonded to erythrocytes along with changes in their shape but without disruption of the membrane structure and also formed complexes with transferrin without inducing conformational changes in the protein’s structure^52^.

Biotransformation products (**3a**-**3c, 5a-5c)** lost the ability of passive permeation through the blood–brain barrier and gained the ability to be actively transported by the P-glycoprotein in contrast to their aglycones **3** and **5**. The performed simulations showed that 2-chloro-2′-hydroxychalcone (**3**) and 3-chloro-2′-hydroxychalcone (**5**) may be inhibitors of some cytochrome P450 enzymes involved in drug metabolism in human hepatocytes (CYP1A2, CYP2C9, CYP2C19) but they are not inhibitors of other cytochrome P450 isozymes (CYP2D6 and CYP3A4). However, their glycosylated derivatives probably do not inhibit CYP1A2, CYP2C9, and CYP2C19, but may inhibit CYP2D6 (**3a** and **5a**) and CYP3A4 (**3a**, **3c**, **5a**, **5b**, and **5c**). Molecules **3**, **3a**, **3b**,** 5**, **5a, 5b**, and **5c** passed drug-likeness estimators (Lipinski, Ghose, Veber, Egan, and Muegge), used by the Swiss-ADME platform, with zero violations. However, compound **3c** violated the Veber estimator (TPSA (Topological Polar Surface Area) > 140) and the Egan estimator (TPSA > 131.6). The Abbott bioavailability score (ABS), which is formulated as the probability that a compound will have > 10% bioavailability in rats or measurable Caco-2 permeability, for all tested compounds was 0.55. Regarding medicinal chemistry simulations, all of the compounds showed zero alerts for PAINS (Pan-Assay Interference Compounds). The prediction results have been collected in Table 5).

**Table 5:** Pharmacokinetics, drug-likeness, and biological activity prediction data from the SwissADME online tool of compounds **3**, **3a-3c**, **5**, **5a-5c**, and **6.**

Activity/Parameter	**3**	**3a**	**3b**	**3c**	**5**	**5a**	**5b**	**5c**	**6**
Lipophilicity consensus Log P_o/w_	3.72	2.12	1.77	1.41	3.71	2.18	1.96	1.79	3.13
Water solubility [mg/ml]	0.0068	0.125	0.149	0.111	0.0068	0.125	0.0786	0.0786	0.0221
Gastrointestinal absorption	h	h	h	Low	h	h	h	h	h
BBB permeant	Yes	No	No	No	Yes	No	No	No	Yes
P-gp substrate	No	Yes	Yes	Yes	No	Yes	Yes	Yes	No
CYP1A2 inhibitor	Yes	No	No	No	Yes	No	No	No	No
CYP2C9 inhibitor	Yes	No	No	No	Yes	No	No	No	Yes
CYP2C19 inhibitor	Yes	No	No	No	Yes	No	No	No	Yes
CYP2D6 inhibitor	No	Yes	No	No	No	Yes	No	No	No
CYP3A4 inhibitor	No	Yes	No	Yes	No	Yes	Yes	Yes	No
Log Kp (skin permeation) [cm/s]	-4.68	-7.58	-7.60	-7.89	-4.68	-7.58	-7.54	-7.54	-4.91
Drug-likeness (Lipinski, Ghose, Veber, Egan, and Muegge)	Yes	Yes	Yes	No Veber (TPSA > 140) Egan (TPSA > 132)	Yes	Yes	Yes	Yes	Yes
Abbott bioavailability score (ABS)	0.55	0.55	0.55	0.55	0.55	0.55	0.55	0.55	0.55
PAINS	0 alert	0 alert	0 alert	0 alert	0 alert	0 alert	0 alert	0 alert	0 alert

#### 2-Chloro-2′-hydroxychalcone (3) and its derivatives (3a, 3b, 3c) Way2Drug Pass Online

The potential biological activities, pharmacological effects, mechanisms of action, and interaction with metabolic enzymes of 2-chloro-2′-hydroxychalcone (**3**) and its biotransformation products (**3a**-**3c**), as well as 3-chloro-2′-hydroxychalcone (**5**) and its biotransformation products (**5a**-**5c**), and for comparison of 2′-hydroxychalcone (**6**) were predicted using Way2Drug Pass Online tool (screens of predictions are shown in Supplementary Information: Figs. S15, S33, S58, S84, S109, S134, S159, S184, and 189)^53,54^. 2′-Hydroxychalcone (**6**), 2-chloro-2′-hydroxychalcone (**3**), and 3-chloro-2′-hydroxychalcone (**5**) exhibited great biological potential as, among others, mucomembranous protectors, membrane integrity agonists, and JAK2 expression inhibitors. The potential biological activities of all of the obtained glycosylated derivatives (**3a**-**3c**, **5a**-**5c**) were quite similar with slight differences in the probability of their occurrence. Among them can be hlighted antimicrobial activity as CDP-glycerol glycerophosphotransferase inhibitor, membrane integrity agonist, anaphylatoxin receptor antagonist, and monophenol monooxygenase inhibitor. Tables 6 and 7 show the most probable biological activities and targets of chalcone aglycons and glycosides. Naturally, to confirm predicted biological activities, it is crucial to perform in vitro and in vivo studies. The simulations allowed us to determine potential targets for further research.

**Table 6:** Predictions of biological activities and targets of compounds **3**, **3a**-**3c**, and** 6** using Way2Drug Pass Online tool.

Activity	**3**		**3a**		**3b**		**3c**		**6**	
	Pa^a^	Pi^b^	Pa	Pi	Pa	Pi	Pa	Pi	Pa	Pi
Mucomembranous protector	0.874	0.006	–	–	–	–	–	–	0.940	0.004
Membrane integrity agonist	0.882	0.016	0.863	0.021	0.883	0.015	0.870	0.019	0.913	0.008
Membrane permeability inhibitor	0.643	0.065	0.760	0.018	0.761	0.018	0.719	0.031	0.696	0.040
JAK2^c^ expression inhibitor	0.843	0.005	0.286	0.147	0.240	0.185	0.279	0.152	0.874	0.004
Antiprotozoal (Leishmania)	0.751	0.007	0.791	0.005	0.798	0.005	0.785	0.005	0.714	0.008
antihypoxic	0.726	0.006	0.380	0.091	0.387	0.087	0.377	0.093	0.828	0.004
CDP-glycerol Glycerophosphotransferase^d^ inhibitor	0.423	0.173	0.936	0.005	0.933	0.005	0.923	0.006	0.583	0.089
Anaphylatoxin^e^ receptor antagonist	0.374	0.124	0.862	0.006	0.837	0.008	0.876	0.005	0.308	0.184
Monophenol monooxygenase^f^ inhibitor	0.462	0.008	0.527	0.006	0.915	0.002	0.593	0.005	0.837	0.003
Cholesterol antagonist	0.404	0.0063	0.763	0.005	0.818	0.004	0.708	0.008	0.416	0.060
Anticarcinogenic	0.298	0.059	0.747	0.007	0.837	0.004	0.740	0.007	0.415	0.028
Antiinfective	0.397	0.047	0.736	0.007	0.837	0.005	0.705	0.007	0.399	0.046
Hepatoprotectant	0.432	0.027	0.716	0.007	0.805	0.004	0.776	0.005	0.511	0.020
G-protein-coupled receptor kinase^g^ inhibitor	0.501	0.049	0.689	0.024	0.827	0.010	0.575	0.038	0.673	0.025
Respiratory analeptic	0.348	0.088	0.612	0.022	0.778	0.010	0.595	0.023	0.448	0.051
Vasoprotector	0.465	0.050	0.626	0.017	0.751	0.007	0.547	0.029	0.649	0.015

^a^Pa—probable activity.

^b^Pi—probable inactivity. Values range from 0 to 1, where 1 represents a 100% probability of Pa or Pi, and 0 represents a 0% probability of Pa or Pi.

^c^JAK2—Janus kinase 2—widely expressed tyrosine kinase responsible for signal transduction, plays a significant role in hematopoiesis, a therapeutic target as its mutations are related to malignant transformations^55^.

^d^CDP-glycerol glycerophosphotransferase—an enzyme responsible for the polymerization of teichoic acid chains, which plays a key role in shaping the bacterial cell, integrating its envelope, creating a bacterial biofilm, and, consequently, the pathogenesis of gram-positive bacteria^56^.

^e^Anaphylatoxin—complement peptide, which plays a role in response to bacterial infections and inflammatory processes, sepsis, ischemia–reperfusion injuries, complex immunological diseases, and asthma^57^.

^f^Monophenol monooxygenase—the rate-limiting enzyme for controlling the production of melanin, its increased activity is associated with the development of malignant melanoma^58^.

^g^G-protein-coupled receptor kinases—family of enzymes that regulate the function of G-protein-coupled receptors that are cellular sensors involved in many physiological processes; increased activity of these kinases contributes to the loss of contractile reserve in the stressed and failing heart, therefore their inhibition is one of the new therapeutic approaches in the treatment of heart failure ^59^.

**Table 7:** Predictions of biological activities and targets of compounds **5**, **5a-5c**, and** 6** using Way2Drug Pass Online tool.

Activity	5		5a		5b		5c		6	
	Pa	Pi	Pa	Pi	Pa	Pi	Pa	Pi	Pa	Pi
Mucomembranous protector	0.940	0.004	–	–	–	–	–	–	0.940	0.004
Membrane integrity agonist	0.910	0.009	0.871	0.019	0.894	0.013	0.812	0.034	0.913	0.008
Membrane permeability inhibitor	0.677	0.049	0.758	0.019	0.769	0.016	0.719	0.031	0.696	0.040
JAK2^c^ expression inhibitor	0.859	0.004	0.259	0.168	0.272	0.157	0.279	0.152	0.874	0.004
Antiprotozoal (Leishmania)	0.690	0.009	0.779	0.005	0.816	0.004	0.758	0.006	0.714	0.008
Antihypoxic	0.783	0.004	0.421	0.067	0.387	0.087	0.342	0.120	0.828	0.004
CDP-glycerol Glycerophosphotransferase^d^ inhibitor	0.398	0.194	0.934	0.005	0.923	0.006	0.928	0.006	0.583	0.089
Anaphylatoxin^e^ receptor antagonist	0.410	0.102	0.863	0.006	0.851	0.007	0.850	0.007	0.308	0.184
Monophenol monooxygenase^f^ inhibitor	0.506	0.006	0.526	0.006	0.669	0.004	0.593	0.005	0.837	0.003
Cholesterol antagonist	0.475	0.044	0.819	0.004	0.866	0.004	0.759	0.005	0.416	0.060
Anticarcinogenic	0.324	0.050	0.753	0.007	0.770	0.006	0.740	0.007	0.415	0.028
Antiinfective	0.421	0.039	0.757	0.005	0.870	0.004	0.898	0.004	0.399	0.046
Hepatoprotectant	0.377	0.036	0.709	0.007	0.767	0.005	0.778	0.004	0.511	0.020
G-protein-coupled receptor kinase^g^ inhibitor	0.492	0.050	0.681	0.024	0.782	0.015	0.672	0.025	0.673	0.025
Respiratory analeptic	0.346	0.089	0.617	0.021	0.671	0.017	0.684	0.016	0.448	0.051
Vasoprotector	0.536	0.031	0.626	0.017	0.658	0.014	0.547	0.029	0.649	0.015

^a^Pa—probable activity.

^b^Pi—probable inactivity. Values range from 0 to 1, where 1 represents a 100% probability of Pa or Pi, and 0 represents a 0% probability of Pa or Pi.

^c^JAK2—Janus kinase 2—widely expressed tyrosine kinase responsible for signal transduction, plays a significant role in hematopoiesis, a therapeutic target as its mutations are related to malignant transformations^55^.

^d^CDP-glycerol glycerophosphotransferase—an enzyme responsible for the polymerization of teichoic acid chains, which plays a key role in shaping the bacterial cell, integrating its envelope, creating a bacterial biofilm, and, consequently, the pathogenesis of gram-positive bacteria ^56^.

^e^Anaphylatoxin—complement peptide, which plays a role in response to bacterial infections and inflammatory processes, sepsis, ischemia–reperfusion injuries, complex immunological diseases, and asthma^57^.

^f^Monophenol monooxygenase—the rate-limiting enzyme for controlling the production of melanin, its increased activity is associated with the development of malignant melanoma^58^.

^g^G-protein-coupled receptor kinases—family of enzymes that regulate the function of G-protein-coupled receptors that are cellular sensors involved in many physiological processes; increased activity of these kinases contributes to the loss of contractile reserve in the stressed and failing heart, therefore their inhibition is one of the new therapeutic approaches in the treatment of heart failure^59^.

Predictions of antimicrobial activity using Way2Drug Services showed medium and little confidence in the proposed target microorganisms. Antibacterial activity prediction using the Way2Drug AntiBac-Pred modelling (screens of predictions are shown in Supplementary Information: Figs. S16, S34, S59, S85, S110, S135, S160, S185, and S190) showed that compounds **3**, **5**, and **6** may inhibit the growth of e.g. *Yersinia pestis* (with confidence 0.3396 for **3**, 0.1619 for **5**, 0.2609 for **6**), *Prevotella intermedia* (with confidence 0.2870 for **3**, 0.3101 for **5**, 0.3671 for **6**), *Streptococcus mutans* (with confidence 0.2597 for **3**, 0.2716 for **5**, 0.3127 for **6**), *Mycobacterium marinum* (with confidence 0.2533 for **3**, 0.3242 for **5**, 0.2768 for **6**), and *Bacillus subtilis subsp. subtilis* str. 168 (with confidence 0.2368 for **3**, 0.2908 for **5**, 0.2531 for **6**). The confidence in these results is rather quite low. On the other hand, compounds **3a**, **3b**, **3c**, **5a**, **5b**, and **5c** may inhibit the growth of bacteria e.g. *Clostridium ramosum* (with confidence 0.6229 for **3a**, 0.6329 for **3b**, 0.6044 for **3c**, 0.6092 for **5a**, 0.5778 for **5b**, 0.6044 for **5c**), *Actinomyces meyeri* (with confidence 0.5801 for **3a**, 0.5855 for **3b**, 0.5603 for **3c**, 0.5661 for **5a**, 0.5306 for **5b**, 0.5603 for **5c**), resistant *Acinetobacter pittii* (with confidence 0.5796 for **3a**, 0.5825 for **3b**, 0.5675 for **3c**, 0.5709 for **5a**, 0.5505 for **5b**, 0.5675 for **5c**), *Mycobacterium mageritense* (with confidence 0.5673 for **3a**, 0.5613 for **3b**, 0.5502 for **3c**, 0.5587 for **5a**, 0.5336 for **5b**, 0.5502 for **5c**), and *Clostridium cadaveris* (with confidence 0.5589 for **3a**, 0.5580 for **3b**, 0.5479 for **3c**, 0.5507 for **5a**, 0.5320 for **5b**, 0.5479 for **5c**).

Antifungal activity prediction using the Way2Drug AntiFun-Pred modelling (screens of predictions are shown in Supplementary Information: Figs. S17, S35, S60, S86, S111, S136, S161, S186, and S191) showed that compounds **3**, **5**, and **6** may inhibit the growth of e.g. *Epidermophyton floccosum* (with confidence 0.2137 for **3**, 0.1685 for **5**, 0.3411 for **6**) and *Trichophyton mentagrophytes* (with confidence 0.1700 for **3**, 0.3044 for **5**, 0.1446 for **6**). On the other hand, compounds **3a**, **3b**, **3c**, **5a**, **5b**, and **5c** may inhibit the growth of fungi e.g. *Rhizopus oryzae* (with confidence 0.5271 for **3a**, 0.4593 for **3b**, 0.4643 for **3c**, 0.5034 for **5a**, 0.4521 for **5b**, 0.4452 for **5c**), *Absidia corymbifera* (with confidence 0.4081 for **3a**, 0.3899 for **3b**, 0.3810 for **3c**, 0.4057 for **5a**, 0.3597 for **5b**, 0.3646 for **5c**), and *Trichophyton mentagrophytes* (with confidence 0.3148 for **3a**, 0.3880 for **3b**, 0.3098 for **3c**, 0.3539 for **5a**, 0.4182 for **5b**, 0.3622 for **5c**).

Antiviral activity prediction using the Way2Drug AntiVir-Pred modelling (screens of predictions are shown in Supplementary Information: Figs. S18, S36, S61, S87, S112, S137, S162, S187, and S192) showed that compounds **3**, **5**, and **6** may act as an inhibitor of e.g. genome polyprotein of Dengue virus type 2 (with confidence 0.4177 for **3**, 0.3681 for **5**, 0.6300 for **6**) and integrase of Human immunodeficiency virus type 2 (with confidence 0.3712 for **3**, 0.4216 for **5**, 0.6291 for **6**). On the other hand, compounds **3a**, **3b**, **3c**, **5a**, **5b**, and **5c** may act as inhibitors of replicase polyprotein 1ab of severe acute respiratory coronavirus 2 (with confidence 0.5485 for **3a**, 0.7174 for **3b**, 0.5416 for **3c**, 0.5102 for **5a**, 0.5781 for **5b**, 0.5703 for **5c**).

#### Antimicrobial activity of 2-chloro-2′-hydroxychalcone (3), 3-chloro-2′-hydroxychalcone (5), 2′-hydroxychalcone (6) and their selected derivatives (3a and 5a)

Predictions have not shown with h confidence target microorganisms of antimicrobial activity of the obtained compounds but pointed out that they may be effective in inhibition of CDP-glycerol glycerophosphotransferase, which is an important enzyme in the pathogenesis of Gram-positive bacteria. Therefore, we decided to make screening tests of the antimicrobial activity of 2-chloro-2′-hydroxychalcone (**3**), 3-chloro-2′-hydroxychalcone (**5**), their main products of fungal biotransformation products, i.e., 2-chlorodihydrochalcone 2′-*O*-β-d-(4″-*O*-methyl)-glucopyranoside (**3a**) and 3-chlorodihydrochalcone 2′-*O*-β-d-(4″-*O*-methyl)-glucopyranoside (**3a**), and for comparision 2′-hydroxychalcone (**6**) using the Bioscreen C (Automated Microbiology Growth Curve Analysis System) against three strains of bacteria *Escherichia coli* 10,536 (Gram-), *Pseudomonas aeruginosa* DSM 939(Gram-), *Staphylococcus aureus* DSM 799 (Gram +), one strain of yeast *Candida albicans* DSM 1386, and three strains of lactic acid bacteria *Lactococcus acidophilus* KBiMZ 01 (Gram +)*, Lactococcus rhamnosus* GG (Gram +)*, Streptococcus thermophilus* KBM—1 (Gram +). The obtained data on the duration of the lag-phase in microbial cultures, both control (only with the tested strain) and with the addition of the tested compounds, as well as the biomass increase, expressed as an increase in optical density (*ΔOD*), are shown in Tables 8 and 9.

**Table 8:** Antimicrobial activity of 2′-hydroxychalcone (**6**), 2-*c*hloro-2′-hydroxychalcone (**3**), 3-chloro-2′-hydroxychalcon (**5)**, their main biotransformation products (**3a** and **5a**), against microbial strains *E. coli* 10,536, *S. aureus* DSM 799, *P. aeruginosa* DSM 939, *C. albicans* DSM 1386.

Compounds and standard drugs	*E. coli* 10,536 (Gram-)		*P. aeruginosa* DSM 939 (Gram-)		*S. aureus* DSM 799 (Gram +)		*C. albicans* DSM 1386 (yeast)	
	lag-phase [h]	ΔOD	lag-phase [h]	ΔOD	lag-phase [h]	ΔOD	lag-phase [h]	ΔOD
Control	1.5	0.51	6.0	0.63	2.0	0.78	10	0.96
Oxytetracycline	–	0	–	0	–	0	–	–
Cycloheximide	–	–	–	–	–	–	–	0
**6**	5	0.25	9	0.36	10	0.32	–	0.11
**3**	–	0	7	0.22	–	0	–	0.06
**3a**	5.0	0.26	13	0.24	30	0.05	–	0.02
**5**	10	0.06	18	0.11	12	0.06	–	0
**5a**	21	0.06	7.0	0.35	–	0	–	0

**Table 9:** Antimicrobial activity of2′-hydroxychalcone (**6**), 2-*c*hloro-2′-hydroxychalcone (**3**), 3-chloro-2′-hydroxychalcone (***5***)*,* their main biotransformation products (**3a** and **5a**) against lactic acid bacteria strains *L. acidophilus* KBiMZ 01, *L. rhamnosus* GG*, S. thermophilus* KBM—1.

Compounds	*L. acidophilus* KBiMZ 01 (Gram +)		*L. rhamnosus* GG (Gram +)		*S. thermophilus* KBM—1 (Gram +)	
	lag-phase [h]	ΔOD	lag-phase [h]	ΔOD	lag-phase [h]	ΔOD
Control	8.0	1.73	2.0	1.83	6.0	1.61
**6**	–	0.01	–	0.05	–	0.06
**3**	25	0.30	–	0	–	0
**3a**	12	0.36	–	0.06	–	0
**5**	–	0	–	0.01	–	0
**5a**	–	0.03	5	0.22	–	0

**Figure 13:**
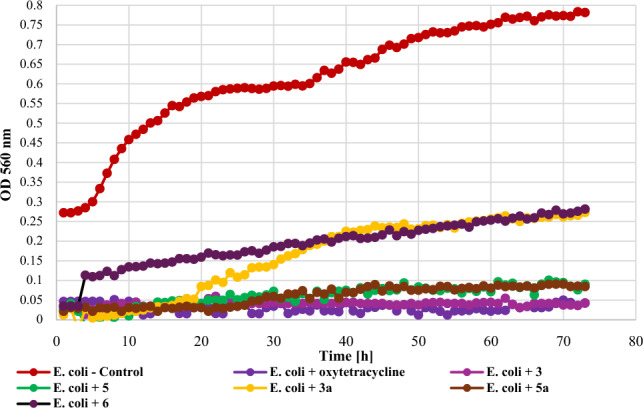
The effect of the action of compounds **3**, **5**, their main biotransformation products (**3a**, **5a**), and **6** on the growth of *E. coli* 10,536.

Biological assays performed on the obtained flavonoids allowed us to evaluate the effect the of introduction of a chlorine atom and 4′-*O*-methylglucosyl moiety into flavonoid structure on antimicrobial activity. The strongest inhibitory effect against *E. coli* 10,536 (Gram -) was observed for flavonoid aglycones **3** (**Δ**OD = 0) and **5** (**Δ**OD = 0.06) as well as for glycoside **5a** (**Δ**OD = 0.06). Other compounds also significantly inhibited bacterial growth, compound **6** with **Δ**OD = 0.25 and **3a** with **Δ**OD = 0.26. The growth of the *E. coli* strain against the tested compounds is shown in Fig. 13.

**Figure 14:**
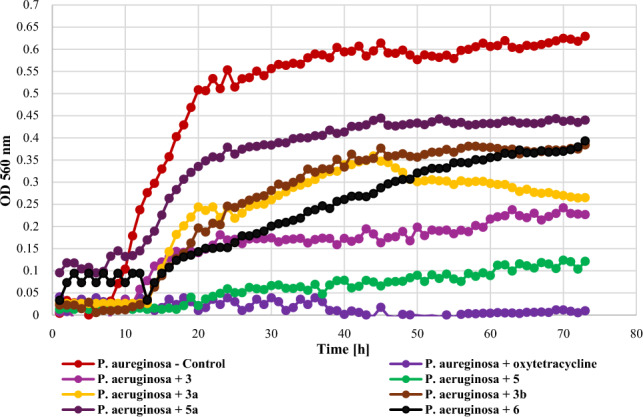
The effect of the action of compounds **3**, **5**, their main biotransformation products (**3a**, **5a**), and **6** on the growth of *P. aeruginosa* DSM 939.

The tested strain of another Gram—bacteria, i.e., *P. aeruginosa* DSM 939 was more resistant to the action of the tested compounds and its growth was most strongly inhibited only by compound **5** (**Δ**OD = 0.11). All other compounds showed significant inhibition of the growth of these bacteria, however, the increase in optical density was not as low as in the case of *E. coli* and ranged from 0.22 to 0.36 (**3** – **Δ**OD = 0.22, **3a** – **Δ**OD = 0.24, **5a** – **Δ**OD = 0.35, and **6** – **Δ**OD = 0.36), while the value of this parameter for the control was 0.63. The growth of the *P. aeruginosa* strain against the tested compounds is shown in Fig. 14.

**Figure 15:**
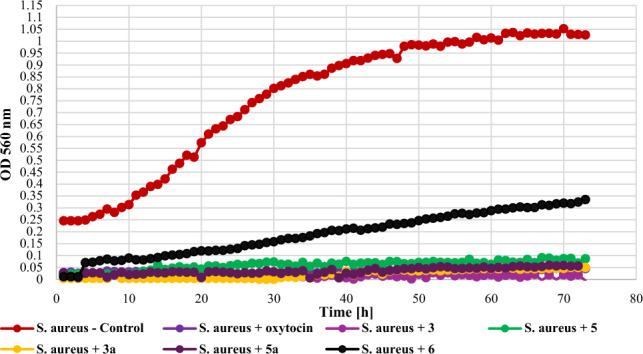
The effect of the action of compounds **3**, **5**, their main biotransformation products (**3a**, **5a**), and **6** on the growth of *S. aureus* DSM 799.

In the case of *S. aureus* DSM 799 (Gram +) both chalcones with a chlorine atom and glycosylated their derivatives exhibited strong inhibitory effects and completely stopped the growth of the tested bacteria strain (**3** – **Δ**OD = 0, **5** – **Δ**OD = 0.06, **3a** – **Δ**OD = 0.05, **5a** – **Δ**OD = 0). 2′-Hydroxychalcone (**6**) was not that effective, which caused an extension of the duration of the adaptation phase and allowed for poor growth with **Δ**OD = 0.32. The growth of the *S. aureus* strain against the tested compounds is shown in Fig. 15.

**Figure 16:**
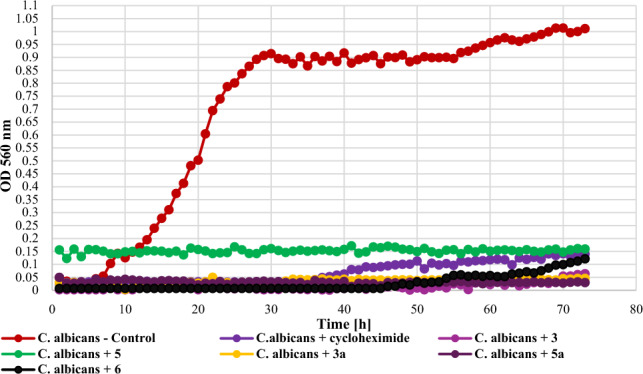
The effect of the action of compounds **3**, **5**, their main biotransformation products (**3a**, **5a**), and **6** on the growth of *C. albicans* DSM 1386.

The yeast *C. albicans* DSM 1386 turned out to be very sensitive to the action of the tested compounds and completely inhibited yeast growth (**3** – ΔOD = 0.06, **5** – ΔOD = 0, **3a** – ΔOD = 0.02, **5a** – ΔOD = 0), a little less effective was 2′-hydroxychalcone (**6**) with ΔOD = 0.11. The growth of the *C. albicans* strain against the tested compounds is shown in Fig. 16.

**Figure 17:**
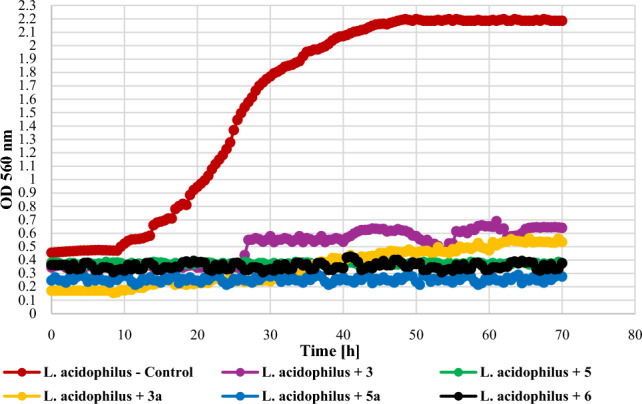
The effect of the action of compounds **3**, **5**, their main biotransformation products (**3a**, **5a**), and **6** on the growth of *L. acidophilus* KBiMZ 01.

**Figure 18:**
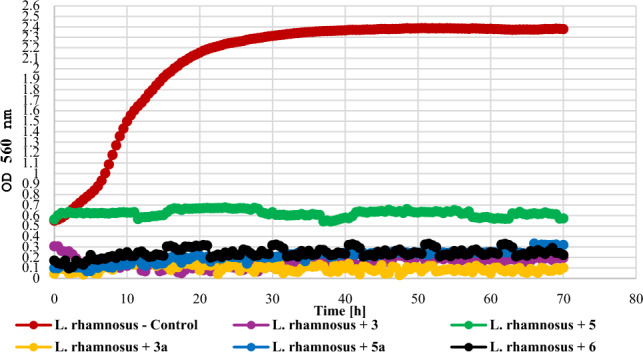
The effect of the action of compounds **3**, **5**, their main biotransformation products (**3a**, **5a**), and **6** on the growth of *L. rhamnosus* GG.

**Figure 19:**
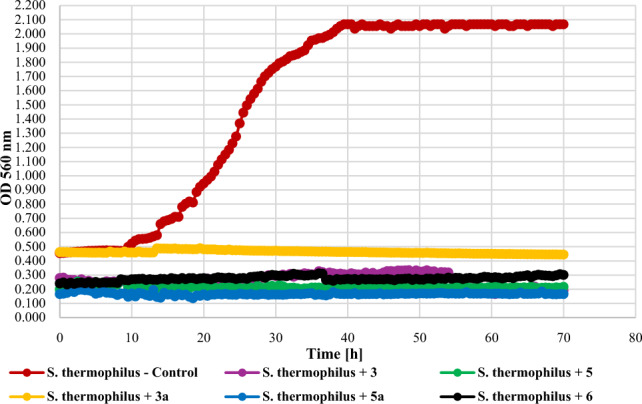
The effect of the action of compounds **3**, **5**, their main biotransformation products (**3a**, **5a**), and **6** on the growth of *S. thermophilus* KBM—1.

The tested compounds at the concentration of 0.1% had different effects on lactic acid bacteria (Gram +) depending on the tested species. The growth of *L. acidophilus* KBiMZ 01 bacteria was completely inhibited only by the compounds **5**, **5a**, and **6**. Compounds **3** and **3a** limited the growth of the tested bacterial strain causing the OD to increase to 0,30 and 0,36, respectively, while the value of this parameter for the control was 1.73. The strains *L. rhamnosous* GG and *S. thermophilus* KBM—1 were very sensitive to the action of the tested compounds. In the case of *L. rhamnosus* PAM, a slight increase in OD, at the level of 0.22, was observed only in the case of compound **5a**. The effect of the action of the tested compounds on lactic acid bacteria is shown in Figs. 17, 18, and 19.

The screening of antimicrobial activity of 2-chloro-2′-hydroxychalcone (**3**), 3-chloro-2′-hydroxychalcone (**5**), their 2′-*O*-*β*-d-(4″-*O*-methyl)-glucopyranosides (**3a** and **5a**) and 2′-hydroxychalcone (**6**) revealed that an introduction of a chlorine atom into the flavonoid structure increased their inhibitory effect againt *E. coli* 10,536, *S. aureus* DSM 799, *P. aeruginosa* DSM 939, and *C. albicans* DSM 1386. A similar effect was observed in the work of Prasad et al., in which chalcones with pharmacophores such as chloro, bromo, and fluoro groups exhibited h antimicrobial and antifungal potential^10^. Likewise, the introduction of a chlorine or bromine atom positively affected the action of pyrazine-based chalcones, which showed anti-staphylococcal and anti-enterococcal activity^60^. In the evaluation of chalcones' inhibitory activity against *Mycobacterium tuberculosis* H37Rv, the substitution of a halogen atom on ring B of 2'-hydroxychalcone increased its anti-tuberculosis activity. Compounds with a halogen at C-3 demonstrated stronger anti-tuberculosis activity than those with a halogen substituent at C-2 or C-4. 3-Chloro-2′-hydroxychalcone exhibited a h 90% inhbition againt *M. tuberculosis* H37RV at a concentration 12.5 mg/mL^61^. These results are consistent with our findings. In the evaluation of the antibacterial activity of flavonoid glycosides (flavonol 3-*O*-glycosides), strong inhibition against Gram-positive bacteria was observed. In addition, Gram-positive bacteria were more sensitive than the Gram-negative bacteria^29^. The results of our studies weren’t so consistent because Gram-negative bacteria *E. coli* 10,536 was also hly sensitive to the action of both flavonoid aglycones and glycosides. Certainly, there is a need for further, more detailed assessment of the antimicrobial activity of the obtained compounds, including learning their mechanisms of action, the effect of the chlorine and glucosyl substituent, and conducting in vivo studies as well.

## Conclusions

Functionalization of chalcones can be achieved by introducing a chlorine atom and glucosyl moiety into their structure, which leads to the compounds with increased water solubility and altered biological activity. In the presented paper, we described the great capacity of entomopathogenic fungi strains *I. fumosorosea* KCH J2 and *B. bassiana* KCH J1.5 to produce dihydrochalcone 4-*O*-methylglucosides from synthetic chalcones, i.e. 2-chloro-2′-hydroxychalcone and 3-chloro-2′-hydroxychalcone. In both biotransformations, the main glycosylated products were obtained with good yields (**3a**—74.5% and **3b**—41% in the case of using culture of *I. fumosorosea* KCH J2) by the attachment of 4″-O-methylglucosyl moiety to the hydroxyl group at C-2′ in ring A and the reduction of the double bond. All six biotransformation products have not been described in the literature until now. The methods described in this paper allow the effective and relatively cheap preparation of large amounts of glycoside derivatives with a chlorine atom for further studies of their biological activity and bioavailability.

We performed computational studies of newly prepared compounds based on the structure–activity relations to preliminary assess their biological activities and accelerate screening procedures for further research. However, in vitro and in vivo biological studies are still necessary for a full understanding of the biological activities, pharmacokinetics, and molecular mechanisms of action of potential new drugs. Our screening of the antimicrobial activity of the obtained compounds showed that the introduction of a chlorine atom in the structure of 2′-hydroxychalcone increases antimicrobial activity against tested strains, but we didn’t observe increased activity of flavonoid glycosides compared to aglycones against Gram-positive bacteria, as suggested by the results of Pass Online simulations. Further research into biological activity, such as membrane integrity agonist, antimicrobial, antiprotozoal, anticancer, anti-inflammatory activity, and metabolic stability, is needed both to assess the usefulness of newly obtained compounds in medicine and to better train algorithms used in chemistry-informatics tools for predicting the biological potential of chemical compounds.

## Materials and methods

### General procedure for the synthesis of biotransformation substrates 3 and 5

Both biotransformation substrates, i.e., 2-chloro-2′-hydroxychalcone (**3**) and 3-chloro-2′-hydroxychalcone (**5**) were obtained using chemical synthesis in the Claisen-Schmidt condensation reaction. The reaction between 2′-hydroxyacetophenone (0.04 mol, 5.4 g) (Sigma-Aldrich, Sant Luis, MO, USA) and 2-chlorobenzaldehyde (0.04 mol, 5.6 g, synthesis of 3) (Sigma-Aldrich, Sant Luis, MO, USA) or 3-chlorobenzaldehyde (0.08 mol, 5.6 g, synthesis of 5) (Sigma-Aldrich, Sant Luis, MO, USA) dissolved in methanol was proceeded under alkaline conditions for 2 h at the boiling point under reflux. After acidifying the reaction mixture, the product was crystallized from 96% ethanol. The chemical reaction schemes are shown above in the section “Results and discussion” (Figs. 1, 2).

The physical data, including color and form, molecular ion mass, molecular formula, melting point (°C), retention time t_R_ (min), retardation factor Rf, and NMR spectral data of the flavonoids **3** and **5** are presented below, in Tables 1, 2, 3, and 4, and the Supplementary Information.

**2-Chloro-2**′**-hydroxychalcone (3).** Yellow crystals (70.9%, 7.3 g), ESISMS m/z 259.0 ([M + H]^+^, C_15_H_11_ClO_2_), mp = 99–100 °C, t_R_ = 18.58, Rf = 0.92; ^1^H-NMR, see Table 1, ^13^C-NMR, see Table 2; Supplementary Information: Figs. S3–S13.

**3-Chloro-2**′**-hydroxychalcone (5).** Yellow crystals (82.3%, 8.5 g), ESIMS m/z 259.0 ([M + H]^+^, C_15_H_11_ClO_2_), mp = 102–103 °C, t_R_ = 18.58, Rf = 0.92, ^1^H-NMR, see Table 3, ^13^C-NMR, see Table 4, Supplementary Information: Figs. S21–S31.

### Microorganisms

Biotransformation of chlorochalcones **3** and **5** was performed using two entomopathogenic filamentous fungi strains belonging to the family *Cordycipitaceae*, i.e., *I. fumosorosea* KCH J2 and *B. bassiana* KCH J1.5. Both strains came from the collection of the Faculty of Biotechnology and Food Microbiology of the Wrocław University of Environmental and Life Sciences in Poland. A detailed description of the collection and reproduction of these fungi as well as their genetic identification were described in our previous works^63,64^. Microbes used in antimicrobial activity screening tests, i.e., *E. coli* 10,536 (Gram-negative), *P. aeruginosa* DSM 939 (Gram-negative), *S. aureus* DSM 799 (Gram-positive), three strains of Gram-positive lactic bacteria *S. thermophilus* KBM—1, *L. acidophilus* KBiMZ 01, and *L. rhamnosus* GG, and also yeast strain *C. albicans* DSM 1386 also belong to the collection of the Faculty of Biotechnology and Food Microbiology of Wrocław University of Environmental and Life Sciences.

### Analysis

Thin-layer chromatography (TLC) and h-performance liquid chromatography (HPLC) were used to assess the course of biotransformation, in particular substrate conversion.

TLC analyses were carried out using TLC Silica gel 60/Kieselguhr F254 (0.2 mm thick) plates (Merck, Darmstadt, Germany) with a mixture of chloroform (POCH, Gliwice, Poland) and methanol (Chempur, Piekary Śląskie, Poland) (9:1 *v/v*) as eluent. The compounds were observed without additional visualization under the ultraviolet lamp at two wavelengths 254 nm and 365 nm^42^*.*

HPLC analyses were performed using a Dionex Ultimate 3000 instrument (Thermo Fisher Scientific, Waltham, MA, USA) with a DAD-3000 diode array detector and analytical octadecyl silica (ODS) 2 column (4.6 mm × 250 mm, Waters, Milford, MA, USA) and dedicated pre-column. The mobile phase was a mixture of 0.1% formic acid (Honeywall, Charlotte, North Carolina, US), in water (Supelco, Darmstadt, Germany) *v*/*v* (A) and 0.1% formic acid in acetonitrile (Supelco, Darmstadt, Germany) *v*/*v* (B). The gradient program was as follows: initial conditions—32.5% B in A, 4 min—40% B in A, 8 min—40% B in A, 10 min—45% B in A, 15 min—95% B in A, 18 min—95% B in A, 19 min—32.5% B in A, 23 min—32.5% B in A. The flow rate was 1 mL/min, the injection volume was 10 µL of samples in a concentration of 1 mg/ml (dissolved in acetonitrile), and the detection wavelengths were 254 (flavonoid glycosides) and 280 nm (flavonoid aglycones)^42^. All compounds are > 95% pure by HPLC analysis.

The separation of the biotransformation products obtained on the semipreparative scale was attained with the use of 500 µm and 1000 µm preparative TLC silica gel plates (Analtech, Gehrden, Germany) with a mixture of chloroform and methanol (9:1 *v*/*v*) as eluent. The compounds were extracted from the selected gel fractions using 20 mL ethyl acetate (Stanlab, Lublin, Poland) 3 times and afterward solvent was evaporated using a rotary evaporator^42^*.*

NMR analyses (^1^H-NMR, ^13^C-NMR, COSY, Heteronuclear Single Quantum Correlation (HSQC), HMBC) were performed using a DRX Avance™ 600 MHz NMR spectrometer (Bruker, Billerica, MA, USA). All samples were dissolved in deuterated acetone to perform analyses.

The confirmation of molecular formulas of all products (**3**, **3a**, **3b**, **3c**, **5**, **5a**, **5b**, and **5c**) was achieved by the mass spectrometry analyses on LC–MS 8045 SHIMADZU Triple Quadrupole Liquid Chromatograph Mass Spectrometer with electrospray ionization (ESI) source (Shimadzu, Kyoto, Japan), as described in our previous works ^42^. For the analyses, the Kinetex column was used (2.6 µm C18 100 Å, 100 mm × 3 mm, Phenomenex, Torrance, CA, USA), which operated at 30 °C. The mobile phase was a mixture of 0.1% formic acid in water *v*/*v* (A) and 0.1% formic acid in acetonitrile *v*/*v* (B). The flow rate was 0.4 mL min^−1^ and the injection volume was 10 µL of samples in a concentration of 1 mg/ml (dissolved in methanol). The gradient program was as follows: initial conditions—80% B in A, 6.5 min—100% B, 7 min—80% B in A. The principal operating parameters for the LC–MS were set as follows: nebulizing gas flow: 3 L min^−1^, heating gas flow: 10 L min^−1^, interface temperature: 300 °C, drying gas flow: 10 L min^−1^, data acquisition range, *m*/*z* 100–500 Da; ionization mode, negative (flavonoid glycosides) and positive (flavonoid aglycones). Data were collected with LabSolutions version 5.97 (Shimadzu, Kyoto, Japan) software ^42^.

### Screening procedure

The purpose of carrying out biotransformations in the screening procedure was to evaluate the time needed for the complete conversion of substrates **3** and **5** for further experiments in the semipreparative scale. The modified Sabouraud medium was used as a growth medium for filamentous fungi (10 g aminobac (BTL, Warsaw, Poland), 30 g glucose (BTL, Warsaw, Poland), and 1 L distilled water). In the first step, the cultures of fungi strains were transferred from potato slants to 300 mL Erlenmeyer flasks with a 100 mL modified Sabouraud liquid medium. The cultures were bred on a rotary shaker (DHN, Warsaw, Poland) with a speed 140 rpm at 25 °C for 72 h. In the second step, 1 mL of the pre-grown cultures were transferred to another 300 mL Erlenmeyer flasks with 100 mL modified Sabouraud liquid medium and incubated in the same conditions. Then, 10 mg of substrate **3** or **5** (dissolved in 0.5 mL of dimethyl sulfoxide (Chempur, Piekary Śląskie, Poland)) was added to each flask with the strain *I. fumosorosea* KCH J2 or *B. bassiana* KCH J1.5. The molar concentration of each biotransformation substrate (**3** and **5)** was 0.39 mM. The samples were collected after 3, 6, and 8 days of substrate incubation. Ethyl acetate (30 ml) was used to extract substrates and products from entire biotransformation cultures. The extracts were dried for 5 min with anhydrous magnesium sulfate (Chempur, Piekary Śląskie, Poland) and then concentrated with the use of a rotary evaporator (Heidolph, Schwabach, Germany) with heating up to 55 °C. The biotransformation experiments were finished after confirmation of complete substrate conversion or lack of further reaction progress (assessed by TLC and HPLC), i.e., 8 days from the start. In parallel, the stability of the substrates under biotransformation conditions and the cultivation of the microorganisms without substrate were performed as controls^42^.

### The semipreparative biotransformation

Semi-preparative scale biotransformation was carried out in 2 L flasks with 500 ml of modified Sabouraud medium. The scale of the performed biotransformations was sufficient to obtain enough amounts of products for NMR analyses and determination of their chemical structures.

Firstly, 1 mL of the preincubation culture of the strain *I. fumosorosea* KCH J2 or *B. bassiana* KCH J1.5 was transferred to the flask, which was subsequently incubated for 72 h under the same conditions as in the case of the screening procedure. Secondly, 50 mg of substrate **3** or **5 (**dissolved in 2.0 mL of dimethyl sulfoxide) was added to each flask with filamentous fungi. The molar concentration of each biotransformation substrate (**3** and **5)** was the same as in the case of the screening procedure, i.e., 0.39 mM. The biotransformation flasks were incubated on the rotary shaker for 8 days, based on estimates from screening tests. However, the experiments were ended after the confirmation of complete substrate conversion. The post-reaction mixtures (entire fungal cultures) were extracted three times with the use of 300 mL of ethyl acetate each time. Subsequently, the joined extracts were dried for 5 min with anhydrous magnesium sulfate, filtered, and afterward, deprived of solvent by its evaporation. The separation and purification of the biotransformation products were achieved using preparative TLC plates as described above. The separate fractions of products were marked under an ultraviolet lamp, separated, and extracted three times with ethyl acetate (20 ml). Afterward, further analyses of their chemical structures by spectroscopic methods were performed. Biotransformation yields were determined based on the masses of the isolated products ^42^.

### Fungal biotransformation products

The physical data, including color and form, molecular ion mass, molecular formula, melting point (°C), retention time t_R_ (min), retardation factor Rf, and NMR spectral data of the fungal biotransformation products **3a**–**3c** and **5a**–**5c** are presented below, above in Tables 1, 2, 3, 4, and the Supplementary Information.

2-Chlorodihydrochalcone 2′-*O*-*β*-d-(4″-*O*-methyl)-glucopyranoside (**3a**): light-yellow crystals, ESIMS m/z 435.1 ([M − H]^−^, C_22_H_25_ClO_7_), mp = 87–89 °C, t_R_ = 10.92, Rf = 0.55; ^1^H-NMR, see Table 1, ^13^C-NMR, see Table 2, Supplementary Information: Figs. S39–S56.

2-Chloro-2′-hydroxydihydrochalcone 5′-*O*-*β*-d-(4″-*O*-methyl)-glucopyranoside (**3b**): white crystals, ESIMS m/z 451.1 ([M − H]^−^, C_22_H_25_ClO_8_), mp = 114–116 °C, t_R_ = 12.24, Rf = 0.48; ^1^H-NMR, see Table 1, ^13^C-NMR, see Table 2, Supplementary Information: Figs. S64–S82.

2-Chloro-2′,3-dihydroxydihydrochalcone 3′-*O*-*β*-d-(4″-*O*-methyl)-glucopyranoside (**3c**): white crystals, ESIMS m/z 467.1 ([M + H]^+^, C_22_H_25_ClO_9_), mp = 188–190 °C, t_R_ = 5.71, Rf = 0.46; ^1^H-NMR, see Table 1, ^13^C-NMR, see Table 2, Supplementary Information: Figs. S90–S107.

3-Chlorodihydrochalcone 2′-*O*-*β*-d-(4″-*O*-methyl)-glucopyranoside (**5a**): light-yellow crystals, ESIMS m/z 437.1 ([M + H]^+^, C_22_H_25_ClO_7_), mp = 116–118 °C, t_R_ = 11.76, Rf = 0.35; ^1^H-NMR, see Table 3, ^13^C-NMR, see Table 4, Supplementary Information: Figs. S115–S132.

3-Chloro-2′-hydroxydihydrochalcone 5′-*O*-*β*-d-(4″-*O*-methyl)-glucopyranoside (**5b**): light-yellow crystals, ESIMS m/z 451.1 ([M − H]^−^, C_22_H_25_ClO_8_), mp = 120–122 °C, t_R_ = 13.11, Rf = 0.36; ^1^H-NMR, see Table 3, ^13^C-NMR, see Table 4, Supplementary Information: Figs. S140–S157.

3-Chloro-2′-hydroxydihydrochalcone 4 -*O*-*β*-d-(4″-*O*-methyl)-glucopyranoside (**5c**): white crystals, ESIMS m/z 451.1 ([M + H]^+^, C_22_H_25_ClO_8_), mp = 63–65 °C, t_R_ = 10.87, Rf = 0.29; ^1^H-NMR, see Table 3, ^13^C-NMR, see Table 4, Supplementary Information: Figs. S165–S182.

### Pharmacokinetics, drug nature, biological activity predictions

The predictions of pharmacokinetics, physicochemical properties, drug nature, and potential biological activities of flavonoids **3**, **3a**-**3c**, **5**, **5a**-**5c, 6** based on their structural formulae were computed using the SwissADME (available online: http://www.swissadme.ch (accessed on October 10^th^) and Way2Drug Pass Online with accompanying services (available online: http://www.way2drug.com/PASSOnline, https://www.way2drug.com/antibac, https://www.way2drug.com/micF, https://www.way2drug.com/antivir (accessed on October 10th). The structures of the molecules were built by ACD Chemsketch 2021.2.0 and saved in a .mol format and, in this form imported into both online tools. The biological activity types in Pass Online are shown as the probability to be revealed (Pa) and not to be revealed (Pi) and are independent values in the range from 0 to 1.

The results of predictions are shown in the Supplementary Information: Figs. S14– S18 (**3**), S32- S36 (**5**), S57- S61 (**3a**), S83- S87 (3b), S108- S112 (**3c**), S133- S137 (**5a**), S158- S162 (**5b**), S183- S187(**5c**), S188- S192 (**6**).

### Antimicrobial activity assays

The study of the antimicrobial activity of compounds **3**, **3a**, **3b**, **5**, **5a,** and **6** (purchased from Sigma-Aldrich, Sant Louis, MO, USA) was performed using the Bioscreen C (Automated Microbiology Growth Curve Analysis System, Helsinki, Finland) against the following strains of bacteria: *E. coli* 10,536 (Gram-negative), *P. aeruginosa* DSM 939 (Gram-negative), *S. aureus* DSM 799 (Gram-positive), three strains of Gram-positive lactic bacteria *S. thermophilus* KBM—1, *L. acidophilus* KBiMZ 01*,* and *L. rhamnosus* GG, and also yeast strain *C. albicans* DSM 1386. All the microorganisms belong to the collection of the Faculty of Biotechnology and Food Microbiology of Wrocław University of Environmental and Life Sciences. The microbiological cultures were cultured before assays for 48 h: bacteria *E. coli* 10,536, *P. aeruginosa* DSM 939, *S. aureus* DSM 799 in a liquid broth LB (5 g of yeast extract, 10 g of tryptone, and 10 g of NaCl, dissolved in 1 L of distilled water (purchased from Merck, Darmstadt, Germany), yeasts *C. albicans* DSM 1386 in liquid YPG medium (10 g of yeast extract, 10 g of bacteriological peptone, and 10 g of glucose, dissolved in 1 L of distilled water, bacteria *S. thermophilus* KBM—1 in M17 medium (0.5 g of ascorbic acid, 5 g of lactose, 0.25 g of magnesium sulfate, 5 g of meat extract, 2.5 g of meat peptone, 19 g of sodium glycerophosphate, 5 g of soya peptone, 2.5 of tryptone, and 2.5 g of yeast extract, dissolved in 1 L of distilled water, bacteria *L. acidophilus* KBiMZ 01 and *L. rhamnosus* GG in liquid MRS medium (4 g of yeast extract, 2 g of triammonium citrate, 5 g of sodium acetate trihydrate, 10 g of sodium acetate trihydrate, 10 g of peptone, 8 g of meat extract, 0.05 g of manganous sulfate tetrahydrate, 0.2 g of magnesium sulfate heptahydrate, 20 g of glucose, 2 g of dipotassium hydrogen phosphate, dissolved in 1 L of distilled water. Tests were performed on 100-well microtiter Bioscreen C plates, with the working volume in each well of 300 μL, comprising 280 μL of culture medium, and 10 μL of microorganism suspension with the final density of $1 x {10}^{6}$ cells/mL, and tested flavonoids 10 μL (dissolved in dimethyl sulfoxide, final concentration 0.1% (m/v)). The temperature was controlled at 30˚C and the optical density was measured automatically 72 h at regular intervals of 60 min at 560 nm in the case of *E. coli* 10,536, *P. aeruginosa* DSM 939, *S. aureus* DSM 799, *C. albicans* DSM 1386 and at 37˚C and the optical density was measured automatically 70 h at regular intervals of 30 min at 560 nm in the case of lactic acid bacteria. Each culture was prepared in three replications and continuously shaken during the experiment. Oxytetracycline—a broad-spectrum oxytetracycline antibiotic (10 mg/ml; Sigma-Aldrich, Saint Louis, MO, USA) and cycloheximide—a naturally occurring fungicide (0,1% (m/v); Sigma-Aldrich, Saint Louis, MO, USA) were used as positive controls. The obtained data was analyzed using Microsoft Excel software. To prepare the growth curves for each strain, the mean values of the absorbance of the medium as a function of time were used. The resulting antimicrobial activity was expressed as the increase in optical density (ΔOD) and was compared to that of the control cultures in the medium with only dimethyl sulfoxide added.

### Supplementary Information

Supplementary Information.

## Data Availability

All relevant data related to this manuscript is provided within the manuscript or supplementary information files.

## References

[1] Zhuang C, Zhang W, Sheng C, Zhang W, Xing C, Miao Z (2017). Chalcone: A privileged structure in medicinal chemistry. Chem. Rev..

[2] Zhang J, Fu XL, Yang N, Wang QA (2013). Synthesis and cytotoxicity of chalcones and 5-deoxyflavonoids. Sci. World J..

[3] Ehrenkranz JRL, Lewis NG, Kahn CR, Roth J (2005). Phlorizin: A review. Diabetes Metab. Res. Rev..

[4] Silva AMS, Tavares HR, Barros AINRA, Cavaleiro JAS (1997). NMR and structural and conformational features of 2′-hydroxychalcones and flavones. Spectrosc. Lett..

[5] Yadav N, Dixit SK, Bhattacharya A, Mishra LC, Sharma M, Awasthi SK, Bhasin VK (2012). Potent antimalarial activity of newly synthesized substituted chalcone analogs in vitro. Chem. Biol. Drug. Des..

[6] Pawlak A, Henklewska M, Suárez BH, Łużny M, Kozłowska E, Obmińska-Mrukowicz B, Janeczko T (2020). Chalcone methoxy derivatives exhibit antiproliferative and proapoptotic activity on canine lymphoma and leukemia cells. Molecules.

[7] Syam S, Abdelwahab SI, Al-Mamary MA, Mohan S (2012). Synthesis of chalcones with anticancer activities. Molecules.

[8] Božić DD, Milenković M, Ivković B, Ćirković I (2014). Antibacterial activity of three newly-synthesized chalcones & synergism with antibiotics against clinical isolates of methicillin-resistant *Staphylococcus Aureus*. Indian J. Med. Res..

[9] Wang Q, Ding ZH, Liu JK, Zheng YT (2004). Xanthohumol, a novel anti-HIV-1 agent purified from hops humulus lupulus. Antiviral Res..

[10] Prasad RY, Rao AL, Rambabu R (2007). Synthesis and antimicrobial activity of some chalcone derivatives. E J. Chem..

[11] Bila NM, Costa-Orlandi CB, Vaso CO, Bonatti JLC, de Assis LR, Regasini LO, Fontana CR, Fusco-Almeida AM, Mendes-Giannini MJS (2021). 2-Hydroxychalcone as a potent compound and photosensitizer against dermatophyte biofilms. Front. Cell. Infect. Microbiol..

[12] Tekale S, Mashele S, Pooe O, Thore S, Kendrekar P, Pawar R, Claborn D, Bhattacharya S, Roy S (2020). Biological role of chalcones in medicinal chemistry. Vector-borne diseases: recent developments in epidemiology and control.

[13] Nowakowska Z (2007). A review of anti-infective and anti-inflammatory chalcones. Eur. J. Med. Chem..

[14] Tajuddeen N, Isah MB, Suleiman MA, van Heerden FR, Ibrahim MA (2018). The chemotherapeutic potential of chalcones against leishmaniases: A review. Int. J. Antimicrob. Agents..

[15] Salehi B, Quispe C, Chamkhi I, El Omari N, Balahbib A, Sharifi-Rad J, Bouyahya A, Akram M, Iqbal M, Docea AO, Caruntu C, Leyva-Gómez G, Dey A, Martorell M, Calina D, López V, Les F (2021). Pharmacological properties of chalcones: A review of preclinical including molecular mechanisms and clinical evidence. Front. pharmacol..

[16] Kostopoulou I, Tzani A, Polyzos NI, Karadendrou MA, Kritsi E, Pontiki E, Liargkova T, Hadjipavlou-Litina D, Zoumpoulakis P, Detsi A (2021). Exploring the 2′-hydroxy-chalcone framework for the development of dual antioxidant and soybean lipoxygenase inhibitory agents. Molecules.

[17] Guvenalp Z, Ozbek H, Karadayi M, Gulluce M, Kuruuzum-Uz A, Salih B, Demirezer O (2015). Two antigenotoxic chalcone glycosides from mentha longifolia subsp. Longifolia. Pharm. Biol..

[18] Dias TA, Duarte CL, Lima CF, Proença MF, Pereira-Wilson C (2013). Superior anticancer activity of halogenated chalcones and flavonols over the natural flavonol quercetin. Eur. J. Med. Chem..

[19] Zhang J, Fu XL, Yang N, Wang QA (2013). Synthesis and cytotoxicity of chalcones and 5-deoxyflavonoids. Sci. World J..

[20] Ribnicky DM, Kuhn P, Poulev A, Logendra S, Zuberi A, Cefalu WT, Raskin I (2009). Improved absorption and bioactivity of active compounds from an anti-diabetic extract of artemisia dracunculus L. Int. J. Pharm..

[21] Bharatham K, Bharatham N, Park KH, Lee KW (2008). Binding Mode analyses and pharmacophore model development for sulfonamide chalcone derivatives, a new class of α-Glucosidase inhibitors. J. Mol. Graph. Model..

[22] Ha MT, Seong SH, Nguyen TD, Cho WK, Ah KJ, Ma JY, Woo MH, Choi JS, Min BS (2018). Chalcone derivatives from the root bark of *Morus alba* L. act as inhibitors of PTP1B and α-glucosidase. Phytochemistry.

[23] Naumann K (1999). Influence of chlorine substituents on biological activity of chemicals. J. Prakt. Chem..

[24] Krych-Madej J, Stawowska K, Gebicka L (2016). Oxidation of flavonoids by hypochlorous acid: Reaction kinetics and antioxidant activity studies. Free Radic. Res..

[25] Selloum L, Djelili H, Sebihi L, Arnhold J (2004). Scavenger effect of flavonols on HOCl-induced luminol chemiluminescence. Luminescence.

[26] Firuzi O, Mladênka P, Petrucci R, Marrosu G, Saso L (2004). Hypochlorite scavenging activity of flavonoids. J. Pharm. Pharmacol..

[27] Freitas M, Ribeiro D, Tomé SM, Silva AMS, Fernandes E (2014). Synthesis of chlorinated flavonoids with anti-inflammatory and pro-apoptotic activities in human neutrophils. Eur. J. Med. Chem..

[28] Thilakarathna SH, Rupasinghe VHP (2013). Flavonoid bioavailability and attempts for bioavailability enhancement. Nutrients.

[29] Xiao J (2017). Dietary flavonoid aglycones and their glycosides: Which show better biological significance?. Crit. Rev. Food. Sci. Nutr..

[30] Hollman P (2004). Absorption, bioavailability, and metabolism of flavonoids. Pharm. Biol..

[31] Dias MC, Pinto DCGA, Silva AMS (2021). Plant flavonoids: Chemical characteristics and biological activity. Molecules.

[32] Meech R, Hu DG, Mckinnon RA, Mubarokah N, Haines AZ, Nair PC, Rowland A, Mackenzie PI (2019). The UDP-Glycosyltransferase (UGT) superfamily: New members, new functions, and novel paradigms. Physiol Rev.

[33] He B, Bai X, Tan Y, Xie W, Feng Y, Yang GY (2022). Glycosyltransferases: Mining, engineering and applications in biosynthesis of glycosylated plant natural products. Synth. Syst. Biotechnol..

[34] Shimoda K, Otsuka T, Morimoto Y, Hamada H, Hamada H (2007). Glycosylation and malonylation of quercetin, epicatechin, and catechin by cultured plant cells. Chem. Lett..

[35] Pandey RP, Li TF, Kim EH, Yamaguchi T, Park Y, Kim JS, Sohng JK (2013). Enzymatic synthesis of novel phloretin glucosides. Appl. Environ. Microbiol..

[36] Li HM, Lee JK, Nie LJ, Huo Q, Ma T, Sohng JK, Hong YS, Wu CZ (2015). Enzymatic synthesis of novel isobavachalcone glucosides via a UDP-glycosyltransferase. Arch. Pharm. Res..

[37] Xie L, Zhang L, Bai J, Yue Q, Zhang M, Li J, Wang C, Xu Y (2019). Methylglucosylation of phenolic compounds by fungal glycosyltransferase-methyltransferase functional modules. J. Agric. Food Chem..

[38] Dou F, Wang Z, Li G, Dun B (2019). Microbial transformation of flavonoids by *Isaria Fumosorosea* ACCC 37814. Molecules.

[39] Sordon S, Popłoński J, Tronina T, Huszcza E (2019). Regioselective *O*-glycosylation of flavonoids by fungi *Beauveria Bassiana*, *Absidia Coerulea* and *Absidia Glauca*. Bioorg. Chem..

[40] Łużny M, Tronina T, Kozłowska E, Dymarska M, Popłoński J, Łyczko J, Kostrzewa-Susłow E, Janeczko T (2020). Biotransformation of methoxyflavones by selected entomopathogenic filamentous fungi. Int. J. Mol. Sci..

[41] Tronina T, Łużny M, Dymarska M, Urbaniak M, Kozłowska E, Piegza M, Stępień Ł, Janeczko T (2023). Glycosylation of quercetin by selected entomopathogenic filamentous fungi and prediction of its products’ bioactivity. Int. J. Mol. Sci..

[42] Krawczyk-Łebek A, Dymarska M, Janeczko T, Kostrzewa-Susłow E (2021). New glycosylated dihydrochalcones obtained by biotransformation of 2^′^-hydroxy-2-methylchalcone in cultures of entomopathogenic filamentous fungi. Int. J. Mol. Sci..

[43] Xie K, Dou X, Chen R, Chen D, Fang C, Xiao Z, Dai J (2017). Two novel fungal phenolic UDP glycosyltransferases from *Absidia coerulea* and *Rhizopus japonicus*. Appl. Environ. Microbiol..

[44] Xie L, Zhang L, Wang C, Wang X, Xu Y, Yu H, Wu P, Li S, Han L, Gunatilaka AAL, Wei X, Lin M, Molnár I, Xu Y (2018). Methylglucosylation of aromatic amino and phenolic moieties of drug-like biosynthons by combinatorial biosynthesis. Proc. Natl. Acad. Sci. USA.

[45] Krawczyk-Łebek A, Dymarska M, Janeczko T, Kostrzewa-susłow E (2022). Glycosylation of methylflavonoids in the cultures of entomopathogenic filamentous fungi as a tool for obtaining new biologically active com pounds. Int. J. Mol. Sci..

[46] Kim HJ, Lee IS (2006). Microbial metabolism of the prenylated chalcone xanthohumol. J. Nat. Prod..

[47] Tronina T, Bartmańska A, Milczarek M, Wietrzyk J, Popłoński J, Rój E, Huszcza E (2013). Antioxidant and antiproliferative activity of glycosides obtained by biotransformation of xanthohumol. Bioorg. Med. Chem. Lett..

[48] Huszcza E, Bartmańska A, Tronina T (2008). Glycosylation of Xanthohumol by Fungi. Z. Naturforsch. C J. Biosci..

[49] Perz M, Krawczyk-Łebek A, Dymarska M, Janeczko T, Kostrzewa-Susłow E (2023). Biotransformation of flavonoids with -NO2, -CH3 groups and -Br, -Cl atoms by entomopathogenic filamentous Fungi. Int. J. Mol. Sci..

[50] Daina A, Michielin O, Zoete V (2017). SwissADME: a free web tool to evaluate pharmacokinetics, drug-likeness and medicinal chemistry friendliness of small molecules. Sci. Rep..

[51] Daina A, Zoete V (2016). A BOILED-Egg to predict gastrointestinal absorption and brain penetration of small molecules. ChemMedChem..

[52] Cyboran-Mikołajczyk S, Bonarska-Kujawa D, Męczarska K, Krawczyk-Łebek A, Kostrzewa-Susłow E (2022). Novel O-methylglucoside derivatives of flavanone in interaction with model membrane and transferrin. Membranes.

[53] Lagunin A, Stepanchikova A, Filimonov D, Poroikov V (2000). PASS: Prediction of activity spectra for biologically active substances. Bioinformatics.

[54] Lagunin A, Filimonov D, Poroikov V (2010). Multi-targeted natural products evaluation based on biological activity prediction with PASS. Curr. Pharm. Des..

[55] Perner F, Perner C, Ernst T, Heidel FH (2019). Roles of JAK2 in aging, inflammation, hematopoiesis and malignant transformation. Cells.

[56] Brown S, Meredith T, Swoboda J, Walker S (2010). *Staphylococcus Aureus* and *Bacillus Subtilis* W23 make polyribitol wall teichoic acids using different enzymatic pathways. Chem. Biol..

[57] Haas P-J, Van Strijp J (2007). Anaphylatoxins their role in bacterial infection and inflammation. Immunol. Res..

[58] Slominski A, Zbytek B, Slominski R (2009). Inhibitors of melanogenesis increase toxicity of cyclophosphamide and lymphocytes against melanoma cells. Int. J. Cancer.

[59] Pfleger J, Gresham K, Koch WJ (2019). G protein-coupled receptor kinases as therapeutic targets in the heart. Nat. Rev. Cardiol..

[60] Konečná K, Diepoltová A, Holmanová P, Jandourek O, Vejsová M, Voxová B, Bárta P, Maixnerová J, Trejtnar F, Kučerová-Chlupáčová M (2022). Comprehensive insight into anti-staphylococcal and anti-enterococcal action of brominated and chlorinated pyrazine-based chalcones. Front. Microbiol..

[61] Lin Y-M, Zhou Y, Flavin MT, Zhou L-M, Nie W, Chen F-C (2002). Chalcones and flavonoids as anti-tuberculosis agents. Bioorg. Med. Chem..

[62] Dymarska M, Grzeszczuk J, Urbaniak M, Janeczko T, Pląskowska E, Stępień Ł, Kostrzewa-Susłow E (2017). Glycosylation of 6-methylflavone by the strain *Isaria Fumosorosea* KCH J2. PLoS One.

[63] Kozłowska E, Urbaniak M, Hoc N, Grzeszczuk J, Dymarska M, Stępień Ł, Pląskowska E, Kostrzewa-Susłow E, Janeczko T (2018). Cascade biotransformation of dehydroepiandrosterone (DHEA) by *Beauveria* species. Sci. Rep..

[64] Krawczyk-Łebek A, Dymarska M, Janeczko T, Kostrzewa-Susłow E (2021). Fungal biotransformation of 2′-methylflavanone and 2′-methylflavone as a method to obtain glycosylated derivatives. Int. J. Mol. Sci..

