# Supplementary material for: Synthesis, fungal biotransformation, and evaluation of the antimicrobial potential of chalcones with a chlorine atom
Source: Sci Rep. 2024 Jul 1;14:15050. doi: 10.1038/s41598-024-65054-9 (PMC11217454; doi:10.1038/s41598-024-65054-9)
Supplement: Supplementary file 1 — Supplementary Information. [file 41598_2024_65054_MOESM1_ESM.docx]

Synthesis, fungal biotransformation, and evaluation of the antimicrobial potential of chalcones with a chlorine atom

**Agnieszka Krawczyk-Łebek^1^*, Barbara Żarowska^2^, Monika Dymarska^1^, Tomasz Janeczko^1^, Edyta Kostrzewa-Susłow^1^**

^1^Department of Food Chemistry and Biocatalysis, Faculty of Biotechnology and Food Science, Wrocław University of Environmental and Life Sciences, Wrocław, Poland

^2^Department of Biotechnology and Food Microbiology, Faculty of Biotechnology and Food Science, Wrocław University of Environmental and Life Sciences, Wrocław, Poland

**Table of contents**

**Figure S1.** MS analysis of 2-chloro-2’-hydroxychalcone (**3**)

**Figure S2.** HPLC analysis of 2-chloro-2’-hydroxychalcone (**3**)

**Figure S3.** ^1^H NMR spectrum (*δ,* acetone-d6, 600 MHz) of 2-chloro-2’-hydroxychalcone (**3**)

**Figure S4** ^1^H NMR spectrum expansion (*δ,* acetone-d6, 600 MHz) of 2-chloro-2’-hydroxychalcone (**3**)

**Figure S5.** ^13^C NMR spectrum (*δ,* acetone-d6, 151 MHz) of 2-chloro-2’-hydroxychalcone (**3**)

**Figure S6.** ^13^C NMR spectrum expansion (*δ,* acetone-d6, 151 MHz) of 2-chloro-2’-hydroxychalcone (**3**)

**Figure S7.** COSY contour map – ^1^H x ^1^H of 2-chloro-2’-hydroxychalcone (**3**)

**Figure S8.** COSY contour map – ^1^H x ^1^H expansion of 2-chloro-2’-hydroxychalcone (**3**)

**Figure S9.** HMQC contour map – ^1^H x ^13^C of 2-chloro-2’-hydroxychalcone (**3**)

**Figure S10.** HMQC contour map – ^1^H x ^13^C expansion of 2-chloro-2’-hydroxychalcone (**3**)

**Figure S11.** HMBC contour map – ^1^H x ^13^C of 2-chloro-2’-hydroxychalcone (**3**)

**Figure S12.** HMBC contour map – ^1^H x ^13^C expansion of 2-chloro-2’-hydroxychalcone (**3**)

**Figure S13.** HMBC contour map – ^1^H x ^13^C expansion of 2-chloro-2’-hydroxychalcone (**3**)

**Figure S14.** 2-Chloro-2’-hydroxychalcone (**3**) physicochemical and ADME parameters prediction using the SwissADME modelling

**Figure S15.** 2-Chloro-2’-hydroxychalcone (**3**) physicochemical biological activity prediction using the Way2Drug Pass online modelling

**Figure S16.** 2-Chloro-2’-hydroxychalcone (**3**) antibacterial activity prediction using the Way2Drug AntiBac-Pred modelling

**Figure S17.** 2-Chloro-2’-hydroxychalcone (**3**) antifungal activity prediction using the Way2Drug AntiFun-Pred modelling

**Figure S18.** 2-Chloro-2’-hydroxychalcone (**3**) antiviral activity prediction using the Way2Drug AntiVir-Pred modelling

**Figure S19.** MS analysis of 3-chloro-2’-hydroxychalcone (**5**)

**Figure S20.** HPLC analysis of 3-chloro-2’-hydroxychalcone (**5**)

**Figure S21** ^1^H NMR spectrum (*δ,* acetone-d6, 600 MHz) of 3-chloro-2’-hydroxychalcone (**5**)

**Figure S22** ^1^H NMR spectrum expansion (*δ,* acetone-d6, 600 MHz) of 3-chloro-2’-hydroxychalcone (**5**)

**Figure S23.** ^13^C NMR spectrum (*δ,* acetone-d6, 151 MHz) of 3-chloro-2’-hydroxychalcone (**5**)

**Figure S24.** ^13^C NMR spectrum expansion (*δ,* acetone-d6, 151 MHz) of 3-chloro-2’-hydroxychalcone (**5**)

**Figure S25.** COSY contour map – ^1^H x ^1^H of 3-chloro-2’-hydroxychalcone (**5**)

**Figure S26.** COSY contour map – ^1^H x ^1^H expansion of 3-chloro-2’-hydroxychalcone (**5**)

**Figure S27.** HMQC contour map – ^1^H x ^13^C of 3-chloro-2’-hydroxychalcone (**5**)

**Figure S28.** HMQC contour map – ^1^H x ^13^C expansion of 3-chloro-2’-hydroxychalcone (**5**)

**Figure S29.** HMBC contour map – ^1^H x ^13^C of 3-chloro-2’-hydroxychalcone (**5**)

**Figure S30.** HMBC contour map – ^1^H x ^13^C expansion of 3-chloro-2’-hydroxychalcone (**5**)

**Figure S31.** HMBC contour map – ^1^H x ^13^C expansion of 3-chloro-2’-hydroxychalcone (**5**)

**Figure S32.** 3-Chloro-2’-hydroxychalcone (**3**) physicochemical and ADME parameters prediction using the SwissADME modelling

**Figure S33.** 3-Chloro-2’-hydroxychalcone (**5**) physicochemical biological activity prediction using the Way2Drug Pass online modelling

**Figure S34.** 3-Chloro-2’-hydroxychalcone (**5**) antibacterial activity prediction using the Way2Drug AntiBac-Pred modelling

**Figure S35.** 3-Chloro-2’-hydroxychalcone (**5**) antifungal activity prediction using the Way2Drug AntiFun-Pred modelling

**Figure S36.** 3-Chloro-2’-hydroxychalcone (**5**) antiviral activity prediction using the Way2Drug AntiVir-Pred modelling

**Figure S37.** MS analysis of 2-chlorodihydrochalcone 2’-*O*-*β*-D-(4’’-*O*-methyl)-glucopyranoside (**3a**)

**Figure S38.** HPLC analysis of 2-chlorodihydrochalcone 2’-*O*-*β*-D-(4’’-*O*-methyl)-glucopyranoside (**3a**)

**Figure S39.** ^1^H NMR spectrum (*δ,* acetone-d6, 600 MHz) of 2-chlorodihydrochalcone 2’-*O*-*β*-D-(4’’-*O*-methyl)-glucopyranoside (**3a**)

**Figure S40** ^1^H NMR spectrum expansion (*δ,* acetone-d6, 600 MHz) of 2-chlorodihydrochalcone 2’-*O*-*β*-D-(4’’-*O*-methyl)-glucopyranoside (**3a**)

**Figure S41** ^1^H NMR spectrum expansion (*δ,* acetone-d6, 600 MHz) of 2-chlorodihydrochalcone 2’-*O*-*β*-D-(4’’-*O*-methyl)-glucopyranoside (**3a**)

**Figure S42.** ^13^C NMR spectrum (*δ,* acetone-d6, 151 MHz) of 2-chlorodihydrochalcone 2’-*O*-*β*-D-(4’’-*O*-methyl)-glucopyranoside (**3a**)

**Figure S43.** ^13^C NMR spectrum expansion (*δ,* acetone-d6, 151 MHz) of 2-chlorodihydrochalcone 2’-*O*-*β*-D-(4’’-*O*-methyl)-glucopyranoside (**3a**)

**Figure S44.** ^13^C NMR spectrum expansion (*δ,* acetone-d6, 151 MHz) of 2-chlorodihydrochalcone 2’-*O*-*β*-D-(4’’-*O*-methyl)-glucopyranoside (**3a**)

**Figure S45.** COSY contour map – ^1^H x ^1^H of 2-chlorodihydrochalcone 2’-*O*-*β*-D-(4’’-*O*-methyl)-glucopyranoside (**3a**)

**Figure S46.** COSY contour map – ^1^H x ^1^H expansion of 2-chlorodihydrochalcone 2’-*O*-*β*-D-(4’’-*O*-methyl)-glucopyranoside (**3a**)

**Figure S47.** COSY contour map – ^1^H x ^1^H expansion of 2-chlorodihydrochalcone 2’-*O*-*β*-D-(4’’-*O*-methyl)-glucopyranoside (**3a**)

**Figure S48.** HMQC contour map – ^1^H x ^13^C of 2-chlorodihydrochalcone 2’-*O*-*β*-D-(4’’-*O*-methyl)-glucopyranoside (**3a**)

**Figure S49.** HMQC contour map – ^1^H x ^13^C expansion of 2-chlorodihydrochalcone 2’-*O*-*β*-D-(4’’-*O*-methyl)-glucopyranoside (**3a**)

**Figure S50.** HMQC contour map – ^1^H x ^13^C expansion of 2-chlorodihydrochalcone 2’-*O*-*β*-D-(4’’-*O*-methyl)-glucopyranoside (**3a**)

**Figure S51.** HMBC contour map – ^1^H x ^13^C of 2-chlorodihydrochalcone 2’-*O*-*β*-D-(4’’-*O*-methyl)-glucopyranoside (**3a**)

**Figure S52.** HMBC contour map – ^1^H x ^13^C expansion of 2-chlorodihydrochalcone 2’-*O*-*β*-D-(4’’-*O*-methyl)-glucopyranoside (**3a**)

**Figure S53.** HMBC contour map – ^1^H x ^13^C expansion of 2-chlorodihydrochalcone 2’-*O*-*β*-D-(4’’-*O*-methyl)-glucopyranoside (**3a**)

**Figure S54.** HMBC contour map – ^1^H x ^13^C expansion of 2-chlorodihydrochalcone 2’-*O*-*β*-D-(4’’-*O*-methyl)-glucopyranoside (**3a**)

**Figure S55.** HMBC contour map – ^1^H x ^13^C expansion of 2-chlorodihydrochalcone 2’-*O*-*β*-D-(4’’-*O*-methyl)-glucopyranoside (**3a**)

**Figure S56.** HMBC contour map – ^1^H x ^13^C expansion of 2-chlorodihydrochalcone 2’-*O*-*β*-D-(4’’-*O*-methyl)-glucopyranoside (**3a**)

**Figure S57.** 2-Chlorodihydrochalcone 2’-*O*-*β*-D-(4’’-*O*-methyl)-glucopyranoside (**3a**) physicochemical and ADME parameters prediction using the SwissADME modelling

**Figure S58.** 2-Chlorodihydrochalcone 2’-*O*-*β*-D-(4’’-*O*-methyl)-glucopyranoside (**3a**) physicochemical biological activity prediction using the Way2Drug Pass online modelling

**Figure S59.** 2-Chlorodihydrochalcone 2’-*O*-*β*-D-(4’’-*O*-methyl)-glucopyranoside (**3a**) antibacterial activity prediction using the Way2Drug AntiBac-Pred modelling

**Figure S60.** 2-Chlorodihydrochalcone 2’-*O*-*β*-D-(4’’-*O*-methyl)-glucopyranoside (**3a**) antifungal activity prediction using the Way2Drug AntiFun-Pred modelling

**Figure S61.** 2-Chlorodihydrochalcone 2’-*O*-*β*-D-(4’’-*O*-methyl)-glucopyranoside (**3a**) antiviral activity prediction using the Way2Drug AntiVir-Pred modelling

**Figure S62.** MS analysis of 2-chloro-2’-hydroxydihydrochalcone 5’-*O*-*β*-D-(4’’-*O*-methyl)-glucopyranoside (**3b**)

**Figure S63.** HPLC analysis of 2-chloro-2’-hydroxydihydrochalcone 5’-*O*-*β*-D-(4’’-*O*-methyl)-glucopyranoside (**3b**)

**Figure S64.** ^1^H NMR spectrum (*δ,* acetone-d6, 600 MHz) of 2-chloro-2’-hydroxydihydrochalcone 5’-*O*-*β*-D-(4’’-*O*-methyl)-glucopyranoside (**3b**)

**Figure S65** ^1^H NMR spectrum expansion (*δ,* acetone-d6, 600 MHz) of 2-chloro-2’-hydroxydihydrochalcone 5’-*O*-*β*-D-(4’’-*O*-methyl)-glucopyranoside (**3b**)

**Figure S66** ^1^H NMR spectrum expansion (*δ,* acetone-d6, 600 MHz) of 2-chloro-2’-hydroxydihydrochalcone 5’-*O*-*β*-D-(4’’-*O*-methyl)-glucopyranoside (**3b**)

**Figure S67.** ^13^C NMR spectrum (*δ,* acetone-d6, 151 MHz) of 2-chloro-2’-hydroxydihydrochalcone 5’-*O*-*β*-D-(4’’-*O*-methyl)-glucopyranoside (**3b**)

**Figure S68.** ^13^C NMR spectrum expansion (*δ,* acetone-d6, 151 MHz) of 2-chloro-2’-hydroxydihydrochalcone 5’-*O*-*β*-D-(4’’-*O*-methyl)-glucopyranoside (**3b**)

**Figure S69.** ^13^C NMR spectrum expansion (*δ,* acetone-d6, 151 MHz) of 2-chloro-2’-hydroxydihydrochalcone 5’-*O*-*β*-D-(4’’-*O*-methyl)-glucopyranoside (**3b**)

**Figure S70.** ^13^C NMR spectrum expansion (*δ,* acetone-d6, 151 MHz) of 2-chloro-2’-hydroxydihydrochalcone 5’-*O*-*β*-D-(4’’-*O*-methyl)-glucopyranoside (**3b**)

**Figure S71.** COSY contour map – ^1^H x ^1^H of 2-chloro-2’-hydroxydihydrochalcone 5’-*O*-*β*-D-(4’’-*O*-methyl)-glucopyranoside (**3b**)

**Figure S72.** COSY contour map – ^1^H x ^1^H expansion of 2-chloro-2’-hydroxydihydrochalcone 5’-*O*-*β*-D-(4’’-*O*-methyl)-glucopyranoside (**3b**)

**Figure S73.** COSY contour map – ^1^H x ^1^H expansion of 2-chloro-2’-hydroxydihydrochalcone 5’-*O*-*β*-D-(4’’-*O*-methyl)-glucopyranoside (**3b**)

**Figure S74.** HMQC contour map – ^1^H x ^13^C of 2-chloro-2’-hydroxydihydrochalcone 5’-*O*-*β*-D-(4’’-*O*-methyl)-glucopyranoside (**3b**)

**Figure S75.** HMQC contour map – ^1^H x ^13^C expansion of 2-chloro-2’-hydroxydihydrochalcone 5’-*O*-*β*-D-(4’’-*O*-methyl)-glucopyranoside (**3b**)

**Figure S76.** HMQC contour map – ^1^H x ^13^C expansion of 2-chloro-2’-hydroxydihydrochalcone 5’-*O*-*β*-D-(4’’-*O*-methyl)-glucopyranoside (**3b**)

**Figure S77.** HMBC contour map – ^1^H x ^13^C of 2-chloro-2’-hydroxydihydrochalcone 5’-*O*-*β*-D-(4’’-*O*-methyl)-glucopyranoside (**3b**)

**Figure S78.** HMBC contour map – ^1^H x ^13^C expansion of 2-chloro-2’-hydroxydihydrochalcone 5’-*O*-*β*-D-(4’’-*O*-methyl)-glucopyranoside (**3b**)

**Figure S79.** HMBC contour map – ^1^H x ^13^C expansion of 2-chloro-2’-hydroxydihydrochalcone 5’-*O*-*β*-D-(4’’-*O*-methyl)-glucopyranoside (**3b**)

**Figure S80.** HMBC contour map – ^1^H x ^13^C expansion of 2-chloro-2’-hydroxydihydrochalcone 5’-*O*-*β*-D-(4’’-*O*-methyl)-glucopyranoside (**3b**)

**Figure S81.** HMBC contour map – ^1^H x ^13^C expansion of 2-chloro-2’-hydroxydihydrochalcone 5’-*O*-*β*-D-(4’’-*O*-methyl)-glucopyranoside (**3b**)

**Figure S82.** HMBC contour map – ^1^H x ^13^C expansion of 2-chloro-2’-hydroxydihydrochalcone 5’-*O*-*β*-D-(4’’-*O*-methyl)-glucopyranoside (**3b**)

**Figure S83.** 2-Chloro-2’-hydroxydihydrochalcone 5’-*O*-*β*-D-(4’’-*O*-methyl)-glucopyranoside (**3b**) physicochemical and ADME parameters prediction using the SwissADME modelling

**Figure S84.** 2-Chloro-2’-hydroxydihydrochalcone 5’-*O*-*β*-D-(4’’-*O*-methyl)-glucopyranoside (**3b**) physicochemical biological activity prediction using the Way2Drug Pass online modelling

**Figure S85.** 2-Chloro-2’-hydroxydihydrochalcone 5’-*O*-*β*-D-(4’’-*O*-methyl)-glucopyranoside (**3b**) antibacterial activity prediction using the Way2Drug AntiBac-Pred modelling

**Figure S86.** 2-Chloro-2’-hydroxydihydrochalcone 5’-*O*-*β*-D-(4’’-*O*-methyl)-glucopyranoside (**3b**) antifungal activity prediction using the Way2Drug AntiFun-Pred modelling

**Figure S87.** 2-Chloro-2’-hydroxydihydrochalcone 5’-*O*-*β*-D-(4’’-*O*-methyl)-glucopyranoside (**3b**) antiviral activity prediction using the Way2Drug AntiVir-Pred modelling

**Figure S88.** MS analysis of 2-chloro-2’,3-dihydroxydihydrochalcone 3’-*O*-*β*-D-(4’’-*O*-methyl)-glucopyranoside (**3c**)

**Figure S89.** HPLC analysis of 2-chloro-2’,3-dihydroxydihydrochalcone 3’-*O*-*β*-D-(4’’-*O*-methyl)-glucopyranoside (**3c**)

**Figure S90.** ^1^H NMR spectrum (*δ,* acetone-d6, 600 MHz) of 2-chloro-2’,3-dihydroxydihydrochalcone 3’-*O*-*β*-D-(4’’-*O*-methyl)-glucopyranoside (**3c**)

**Figure S91** ^1^H NMR spectrum expansion (*δ,* acetone-d6, 600 MHz) of 2-chloro-2’,3-dihydroxydihydrochalcone 3’-*O*-*β*-D-(4’’-*O*-methyl)-glucopyranoside (**3c**)

**Figure S92** ^1^H NMR spectrum expansion (*δ,* acetone-d6, 600 MHz) of 2-chloro-2’,3-dihydroxydihydrochalcone 3’-*O*-*β*-D-(4’’-*O*-methyl)-glucopyranoside (**3c**)

**Figure S93.** ^13^C NMR spectrum (*δ,* acetone-d6, 151 MHz) of 2-chloro-2’,3-dihydroxydihydrochalcone 3’-*O*-*β*-D-(4’’-*O*-methyl)-glucopyranoside (**3c**)

**Figure S94.** ^13^C NMR spectrum expansion (*δ,* acetone-d6, 151 MHz) of 2-chloro-2’,3-dihydroxydihydrochalcone 3’-*O*-*β*-D-(4’’-*O*-methyl)-glucopyranoside (**3c**)

**Figure S95.** ^13^C NMR spectrum expansion (*δ,* acetone-d6, 151 MHz) of 2-chloro-2’,3-dihydroxydihydrochalcone 3’-*O*-*β*-D-(4’’-*O*-methyl)-glucopyranoside (**3c**)

**Figure S96.** COSY contour map – ^1^H x ^1^H of 2-chloro-2’,3-dihydroxydihydrochalcone 3’-*O*-*β*-D-(4’’-*O*-methyl)-glucopyranoside (**3c**)

**Figure S97.** COSY contour map – ^1^H x ^1^H expansion of 2-chloro-2’,3-dihydroxydihydrochalcone 3’-*O*-*β*-D-(4’’-*O*-methyl)-glucopyranoside (**3c**)

**Figure S98.** COSY contour map – ^1^H x ^1^H expansion of 2-chloro-2’,3-dihydroxydihydrochalcone 3’-*O*-*β*-D-(4’’-*O*-methyl)-glucopyranoside (**3c**)

**Figure S99.** HMQC contour map – ^1^H x ^13^C of 2-chloro-2’,3-dihydroxydihydrochalcone 3’-*O*-*β*-D-(4’’-*O*-methyl)-glucopyranoside (**3c**)

**Figure S100.** HMQC contour map – ^1^H x ^13^C expansion of 2-chloro-2’,3-dihydroxydihydrochalcone 3’-*O*-*β*-D-(4’’-*O*-methyl)-glucopyranoside (**3c**)

**Figure S101** HMQC contour map – ^1^H x ^13^C expansion of 2-chloro-2’,3-dihydroxydihydrochalcone 3’-*O*-*β*-D-(4’’-*O*-methyl)-glucopyranoside (**3c**)

**Figure S102.** HMBC contour map – ^1^H x ^13^C of 2-chloro-2’,3-dihydroxydihydrochalcone 3’-*O*-*β*-D-(4’’-*O*-methyl)-glucopyranoside (**3c**)

**Figure S103.** HMBC contour map – ^1^H x ^13^C expansion of 2-chloro-2’,3-dihydroxydihydrochalcone 3’-*O*-*β*-D-(4’’-*O*-methyl)-glucopyranoside (**3c**)

**Figure S104.** HMBC contour map – ^1^H x ^13^C expansion of 2-chloro-2’,3-dihydroxydihydrochalcone 3’-*O*-*β*-D-(4’’-*O*-methyl)-glucopyranoside (**3c**)

**Figure S105.** HMBC contour map – ^1^H x ^13^C expansion of 2-chloro-2’,3-dihydroxydihydrochalcone 3’-*O*-*β*-D-(4’’-*O*-methyl)-glucopyranoside (**3c**)

**Figure S106.** HMBC contour map – ^1^H x ^13^C expansion of 2-chloro-2’,3-dihydroxydihydrochalcone 3’-*O*-*β*-D-(4’’-*O*-methyl)-glucopyranoside (**3c**)

**Figure S107.** HMBC contour map – ^1^H x ^13^C expansion of 2-chloro-2’,3-dihydroxydihydrochalcone 3’-*O*-*β*-D-(4’’-*O*-methyl)-glucopyranoside (**3c**)

**Figure S108.** 2-Chloro-2’,3-dihydroxydihydrochalcone 3’-*O*-*β*-D-(4’’-*O*-methyl)-glucopyranoside (**3c**) physicochemical and ADME parameters prediction using the SwissADME modelling

**Figure S109.** 2-Chloro-2’,3-dihydroxydihydrochalcone 3’-*O*-*β*-D-(4’’-*O*-methyl)-glucopyranoside (**3c**) physicochemical biological activity prediction using the Way2Drug Pass online modelling

**Figure S110.** 2-Chloro-2’,3-dihydroxydihydrochalcone 3’-*O*-*β*-D-(4’’-*O*-methyl)-glucopyranoside (**3c**) antibacterial activity prediction using the Way2Drug AntiBac-Pred modelling

**Figure S111.** 2-Chloro-2’,3-dihydroxydihydrochalcone 3’-*O*-*β*-D-(4’’-*O*-methyl)-glucopyranoside (**3c**) antifungal activity prediction using the Way2Drug AntiFun-Pred modelling

**Figure S112.** 2-Chloro-2’,3-dihydroxydihydrochalcone 3’-*O*-*β*-D-(4’’-*O*-methyl)-glucopyranoside (**3c**) antiviral activity prediction using the Way2Drug AntiVir-Pred modelling

**Figure S113.** MS analysis of 3-chlorodihydrochalcone 2’-*O*-*β*-D-(4’’-*O*-methyl)-glucopyranoside (**5a**)

**Figure S114.** HPLC analysis of 3-chlorodihydrochalcone 2’-*O*-*β*-D-(4’’-*O*-methyl)-glucopyranoside (**5a**)

**Figure S115.** ^1^H NMR spectrum (*δ,* acetone-d6, 600 MHz) of 3-chlorodihydrochalcone 2’-*O*-*β*-D-(4’’-*O*-methyl)-glucopyranoside (**5a**)

**Figure S116.** ^1^H NMR spectrum expansion (*δ,* acetone-d6, 600 MHz) of 3-chlorodihydrochalcone 2’-*O*-*β*-D-(4’’-*O*-methyl)-glucopyranoside (**5a**)

**Figure S117.** ^1^H NMR spectrum expansion (*δ,* acetone-d6, 600 MHz) of 3-chlorodihydrochalcone 2’-*O*-*β*-D-(4’’-*O*-methyl)-glucopyranoside (**5a**)

**Figure S118.** ^13^C NMR spectrum (*δ,* acetone-d6, 151 MHz) of 3-chlorodihydrochalcone 2’-*O*-*β*-D-(4’’-*O*-methyl)-glucopyranoside (**5a**)

**Figure S119.** ^13^C NMR spectrum expansion (*δ,* acetone-d6, 151 MHz) of 3-chlorodihydrochalcone 2’-*O*-*β*-D-(4’’-*O*-methyl)-glucopyranoside (**5a**)

**Figure S120.** ^13^C NMR spectrum expansion (*δ,* acetone-d6, 151 MHz) of 3-chlorodihydrochalcone 2’-*O*-*β*-D-(4’’-*O*-methyl)-glucopyranoside (**5a**)

**Figure S121.** COSY contour map – ^1^H x ^1^H of 3-chlorodihydrochalcone 2’-*O*-*β*-D-(4’’-*O*-methyl)-glucopyranoside (**5a**)

**Figure S122.** COSY contour map – ^1^H x ^1^H expansion of 3-chlorodihydrochalcone 2’-*O*-*β*-D-(4’’-*O*-methyl)-glucopyranoside (**5a**)

**Figure S123.** COSY contour map – ^1^H x ^1^H expansion of 3-chlorodihydrochalcone 2’-*O*-*β*-D-(4’’-*O*-methyl)-glucopyranoside (**5a**)

**Figure S124.** HMQC contour map – ^1^H x ^13^C of 3-chlorodihydrochalcone 2’-*O*-*β*-D-(4’’-*O*-methyl)-glucopyranoside (**5a**)

**Figure S125.** HMQC contour map – ^1^H x ^13^C expansion of 3-chlorodihydrochalcone 2’-*O*-*β*-D-(4’’-*O*-methyl)-glucopyranoside (**5a**)

**Figure S126.** HMQC contour map – ^1^H x ^13^C expansion of 3-chlorodihydrochalcone 2’-*O*-*β*-D-(4’’-*O*-methyl)-glucopyranoside (**5a**)

**Figure S127.** HMBC contour map – ^1^H x ^13^C of 3-chlorodihydrochalcone 2’-*O*-*β*-D-(4’’-*O*-methyl)-glucopyranoside (**5a**)

**Figure S128.** HMBC contour map – ^1^H x ^13^C expansion of 3-chlorodihydrochalcone 2’-*O*-*β*-D-(4’’-*O*-methyl)-glucopyranoside (**5a**)

**Figure S129.** HMBC contour map – ^1^H x ^13^C expansion of 3-chlorodihydrochalcone 2’-*O*-*β*-D-(4’’-*O*-methyl)-glucopyranoside (**5a**)

**Figure S130.** HMBC contour map – ^1^H x ^13^C expansion of 3-chlorodihydrochalcone 2’-*O*-*β*-D-(4’’-*O*-methyl)-glucopyranoside (**5a**)

**Figure S131.** HMBC contour map – ^1^H x ^13^C expansion of 3-chlorodihydrochalcone 2’-*O*-*β*-D-(4’’-*O*-methyl)-glucopyranoside (**5a**)

**Figure S132.** HMBC contour map – ^1^H x ^13^C expansion of 3-chlorodihydrochalcone 2’-*O*-*β*-D-(4’’-*O*-methyl)-glucopyranoside (**5a**)

**Figure S133.** 3-Chlorodihydrochalcone 2’-*O*-*β*-D-(4’’-*O*-methyl)-glucopyranoside (**5a**) physicochemical and ADME parameters prediction using the SwissADME modelling

**Figure S134.** 3-Chlorodihydrochalcone 2’-*O*-*β*-D-(4’’-*O*-methyl)-glucopyranoside (**5a**) physicochemical biological activity prediction using the Way2Drug Pass online modelling

**Figure S135.** 3-Chlorodihydrochalcone 2’-*O*-*β*-D-(4’’-*O*-methyl)-glucopyranoside (**5a**) antibacterial activity prediction using the Way2Drug AntiBac-Pred modelling

**Figure S136.** 3-Chlorodihydrochalcone 2’-*O*-*β*-D-(4’’-*O*-methyl)-glucopyranoside (**5a**) antifungal activity prediction using the Way2Drug AntiFun-Pred modelling

**Figure S137.** 3-Chlorodihydrochalcone 2’-*O*-*β*-D-(4’’-*O*-methyl)-glucopyranoside (**5a**) antiviral activity prediction using the Way2Drug AntiVir-Pred modelling

**Figure S138.** MS analysis of 3-chloro-2’-hydroxydihydrochalcone 5’-*O*-*β*-D-(4’’-*O*-methyl)-glucopyranoside (**5b**)

**Figure S139.** HPLC analysis of 3-chloro-2’-hydroxydihydrochalcone 5’-*O*-*β*-D-(4’’-*O*-methyl)-glucopyranoside (**5b**)

**Figure S140.** ^1^H NMR spectrum (*δ,* acetone-d6, 600 MHz) of 3-chloro-2’-hydroxydihydrochalcone 5’-*O*-*β*-D-(4’’-*O*-methyl)-glucopyranoside (**5b**)

**Figure S141.** ^1^H NMR spectrum expansion (*δ,* acetone-d6, 600 MHz) of 3-chloro-2’-hydroxydihydrochalcone 5’-*O*-*β*-D-(4’’-*O*-methyl)-glucopyranoside (**5b**)

**Figure S142.** ^1^H NMR spectrum expansion (*δ,* acetone-d6, 600 MHz) of 3-chloro-2’-hydroxydihydrochalcone 5’-*O*-*β*-D-(4’’-*O*-methyl)-glucopyranoside (**5b**)

**Figure S143.** ^13^C NMR spectrum (*δ,* acetone-d6, 151 MHz) of 3-chloro-2’-hydroxydihydrochalcone 5’-*O*-*β*-D-(4’’-*O*-methyl)-glucopyranoside (**5b**)

**Figure S144.** ^13^C NMR spectrum expansion (*δ,* acetone-d6, 151 MHz) of 3-chloro-2’-hydroxydihydrochalcone 5’-*O*-*β*-D-(4’’-*O*-methyl)-glucopyranoside (**5b**)

**Figure S145.** ^13^C NMR spectrum expansion (*δ,* acetone-d6, 151 MHz) of 3-chloro-2’-hydroxydihydrochalcone 5’-*O*-*β*-D-(4’’-*O*-methyl)-glucopyranoside (**5b**)

**Figure S146.** COSY contour map – ^1^H x ^1^H of 3-chloro-2’-hydroxydihydrochalcone 5’-*O*-*β*-D-(4’’-*O*-methyl)-glucopyranoside (**5b**)

**Figure S147.** COSY contour map – ^1^H x ^1^H expansion of 3-chloro-2’-hydroxydihydrochalcone 5’-*O*-*β*-D-(4’’-*O*-methyl)-glucopyranoside (**5b**)

**Figure S148.** COSY contour map – ^1^H x ^1^H expansion of 3-chloro-2’-hydroxydihydrochalcone 5’-*O*-*β*-D-(4’’-*O*-methyl)-glucopyranoside (**5b**)

**Figure S149.** HMQC contour map – ^1^H x ^13^C of 3-chloro-2’-hydroxydihydrochalcone 5’-*O*-*β*-D-(4’’-*O*-methyl)-glucopyranoside (**5b**)

**Figure S150.** HMQC contour map – ^1^H x ^13^C expansion of 3-chloro-2’-hydroxydihydrochalcone 5’-*O*-*β*-D-(4’’-*O*-methyl)-glucopyranoside (**5b**)

**Figure S151.** HMQC contour map – ^1^H x ^13^C expansion of 3-chloro-2’-hydroxydihydrochalcone 5’-*O*-*β*-D-(4’’-*O*-methyl)-glucopyranoside (**5b**)

**Figure S152.** HMBC contour map – ^1^H x ^13^C of 3-chloro-2’-hydroxydihydrochalcone 5’-*O*-*β*-D-(4’’-*O*-methyl)-glucopyranoside (**5b**)

**Figure S153.** HMBC contour map – ^1^H x ^13^C expansion of 3-chloro-2’-hydroxydihydrochalcone 5’-*O*-*β*-D-(4’’-*O*-methyl)-glucopyranoside (**5b**)

**Figure S154.** HMBC contour map – ^1^H x ^13^C expansion of 3-chloro-2’-hydroxydihydrochalcone 5’-*O*-*β*-D-(4’’-*O*-methyl)-glucopyranoside (**5b**)

**Figure S155.** HMBC contour map – ^1^H x ^13^C expansion of 3-chloro-2’-hydroxydihydrochalcone 5’-*O*-*β*-D-(4’’-*O*-methyl)-glucopyranoside (**5b**)

**Figure S156.** HMBC contour map – ^1^H x ^13^C expansion of 3-chloro-2’-hydroxydihydrochalcone 5’-*O*-*β*-D-(4’’-*O*-methyl)-glucopyranoside (**5b**)

**Figure S157.** HMBC contour map – ^1^H x ^13^C expansion of 3-chloro-2’-hydroxydihydrochalcone 5’-*O*-*β*-D-(4’’-*O*-methyl)-glucopyranoside (**5b**)

**Figure S158.** 3-Chloro-2’-hydroxydihydrochalcone 5’-*O*-*β*-D-(4’’-*O*-methyl)-glucopyranoside (**5b**) physicochemical and ADME parameters prediction using the SwissADME modelling

**Figure S159.** 3-Chloro-2’-hydroxydihydrochalcone 5’-*O*-*β*-D-(4’’-*O*-methyl)-glucopyranoside (**5b**) physicochemical biological activity prediction using the Way2Drug Pass online modelling

**Figure S160.** 3-Chloro-2’-hydroxydihydrochalcone 5’-*O*-*β*-D-(4’’-*O*-methyl)-glucopyranoside (**5b**) antibacterial activity prediction using the Way2Drug AntiBac-Pred modelling

**Figure S161.** 3-Chloro-2’-hydroxydihydrochalcone 5’-*O*-*β*-D-(4’’-*O*-methyl)-glucopyranoside (**5b**) antifungal activity prediction using the Way2Drug AntiFun-Pred modelling

**Figure S162.** 3-Chloro-2’-hydroxydihydrochalcone 5’-*O*-*β*-D-(4’’-*O*-methyl)-glucopyranoside (**5b**) antiviral activity prediction using the Way2Drug AntiVir-Pred modelling

**Figure S163.** MS analysis of 3-chloro-2’-hydroxydihydrochalcone 4-*O*-*β*-D-(4’’-*O*-methyl)-glucopyranoside (**5c**)

**Figure S164.** HPLC analysis of 3-chloro-2’-hydroxydihydrochalcone 4-*O*-*β*-D-(4’’-*O*-methyl)-glucopyranoside (**5c**)

**Figure S165.** ^1^H NMR spectrum (*δ,* acetone-d6, 600 MHz) of 3-chloro-2’-hydroxydihydrochalcone 4-*O*-*β*-D-(4’’-*O*-methyl)-glucopyranoside (**5c**)

**Figure S166.** ^1^H NMR spectrum expansion (*δ,* acetone-d6, 600 MHz) of 3-chloro-2’-hydroxydihydrochalcone 4-*O*-*β*-D-(4’’-*O*-methyl)-glucopyranoside (**5c**)

**Figure S167.** ^1^H NMR spectrum expansion (*δ,* acetone-d6, 600 MHz) of 3-chloro-2’-hydroxydihydrochalcone 4-*O*-*β*-D-(4’’-*O*-methyl)-glucopyranoside (**5c**)

**Figure S168.** ^13^C NMR spectrum (*δ,* acetone-d6, 151 MHz) of 3-chloro-2’-hydroxydihydrochalcone 4-*O*-*β*-D-(4’’-*O*-methyl)-glucopyranoside (**5c**)

**Figure S169.** ^13^C NMR spectrum expansion (*δ,* acetone-d6, 151 MHz) of 3-chloro-2’-hydroxydihydrochalcone 4-*O*-*β*-D-(4’’-*O*-methyl)-glucopyranoside (**5c**)

**Figure S170.** ^13^C NMR spectrum expansion (*δ,* acetone-d6, 151 MHz) of 3-chloro-2’-hydroxydihydrochalcone 4-*O*-*β*-D-(4’’-*O*-methyl)-glucopyranoside (**5c**)

**Figure S171.** COSY contour map – ^1^H x ^1^H of 3-chloro-2’-hydroxydihydrochalcone 4-*O*-*β*-D-(4’’-*O*-methyl)-glucopyranoside (**5c**)

**Figure S172.** COSY contour map – ^1^H x ^1^H expansion of 3-chloro-2’-hydroxydihydrochalcone 4-*O*-*β*-D-(4’’-*O*-methyl)-glucopyranoside (**5c**)

**Figure S173.** COSY contour map – ^1^H x ^1^H expansion of 3-chloro-2’-hydroxydihydrochalcone 4-*O*-*β*-D-(4’’-*O*-methyl)-glucopyranoside (**5c**)

**Figure S174.** HMQC contour map – ^1^H x ^13^C of 3-chloro-2’-hydroxydihydrochalcone 4-*O*-*β*-D-(4’’-*O*-methyl)-glucopyranoside (**5c**)

**Figure S175.** HMQC contour map – ^1^H x ^13^C expansion of 3-chloro-2’-hydroxydihydrochalcone 4-*O*-*β*-D-(4’’-*O*-methyl)-glucopyranoside (**5c**)

**Figure S176.** HMQC contour map – ^1^H x ^13^C expansion of 3-chloro-2’-hydroxydihydrochalcone 4-*O*-*β*-D-(4’’-*O*-methyl)-glucopyranoside (**5c**)

**Figure S177.** HMBC contour map – ^1^H x ^13^C of 3-chloro-2’-hydroxydihydrochalcone 4-*O*-*β*-D-(4’’-*O*-methyl)-glucopyranoside (**5c**)

**Figure S178.** HMBC contour map – ^1^H x ^13^C expansion of 3-chloro-2’-hydroxydihydrochalcone 4-*O*-*β*-D-(4’’-*O*-methyl)-glucopyranoside (**5c**)

**Figure S179.** HMBC contour map – ^1^H x ^13^C expansion of 3-chloro-2’-hydroxydihydrochalcone 4-*O*-*β*-D-(4’’-*O*-methyl)-glucopyranoside (**5c**)

**Figure S180.** HMBC contour map – ^1^H x ^13^C expansion of 3-chloro-2’-hydroxydihydrochalcone 4-*O*-*β*-D-(4’’-*O*-methyl)-glucopyranoside (**5c**)

**Figure S181.** HMBC contour map – ^1^H x ^13^C expansion of 3-chloro-2’-hydroxydihydrochalcone 4-*O*-*β*-D-(4’’-*O*-methyl)-glucopyranoside (**5c**)

**Figure S182.** HMBC contour map – ^1^H x ^13^C expansion of 3-chloro-2’-hydroxydihydrochalcone 4-*O*-*β*-D-(4’’-*O*-methyl)-glucopyranoside (**5c**)

**Figure S183.** 3-Chloro-2’-hydroxydihydrochalcone 4-*O*-*β*-D-(4’’-*O*-methyl)-glucopyranoside (**5c**) physicochemical and ADME parameters prediction using the SwissADME modelling

**Figure S184.** 3-Chloro-2’-hydroxydihydrochalcone 4-*O*-*β*-D-(4’’-*O*-methyl)-glucopyranoside (**5c**) physicochemical biological activity prediction using the Way2Drug Pass online modelling

**Figure S185.** 3-Chloro-2’-hydroxydihydrochalcone 4-*O*-*β*-D-(4’’-*O*-methyl)-glucopyranoside (**5c**) antibacterial activity prediction using the Way2Drug AntiBac-Pred modelling

**Figure S186.** 3-Chloro-2’-hydroxydihydrochalcone 4-*O*-*β*-D-(4’’-*O*-methyl)-glucopyranoside (**5c**) antifungal activity prediction using the Way2Drug AntiFun-Pred modelling

**Figure S187.** 3-Chloro-2’-hydroxydihydrochalcone 4-*O*-*β*-D-(4’’-*O*-methyl)-glucopyranoside (**5c**) antiviral activity prediction using the Way2Drug AntiVir-Pred modelling

**Figure S188.** 2’-Hydroxychalcone (**6**) physicochemical and ADME parameters prediction using the SwissADME modelling

**Figure S189.** 2’-Hydroxychalcone (**6**) physicochemical biological activity prediction using the Way2Drug Pass online modelling

**Figure S190.** 2’-Hydroxychalcone (**6**) activity prediction using the Way2Drug AntiBac-Pred modelling

**Figure S191.** 2’-Hydroxychalcone (**6**) antifungal activity prediction using the Way2Drug AntiFun-Pred modelling

**Figure S192.** 2’-Hydroxychalcone (**6**) antiviral activity prediction using the Way2Drug AntiVir-Pred modelling


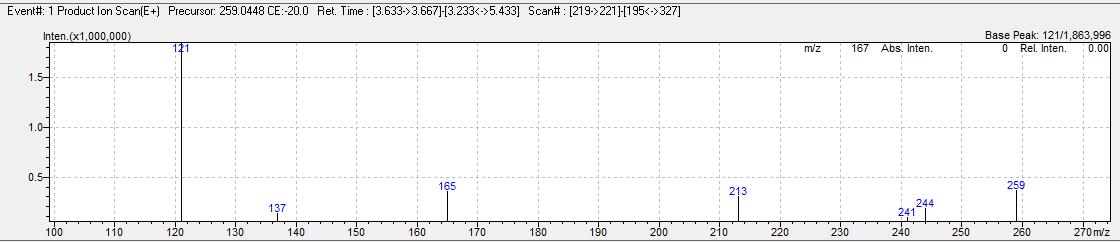


**Figure S1.** MS analysis of 2-chloro-2’-hydroxychalcone (**3**)


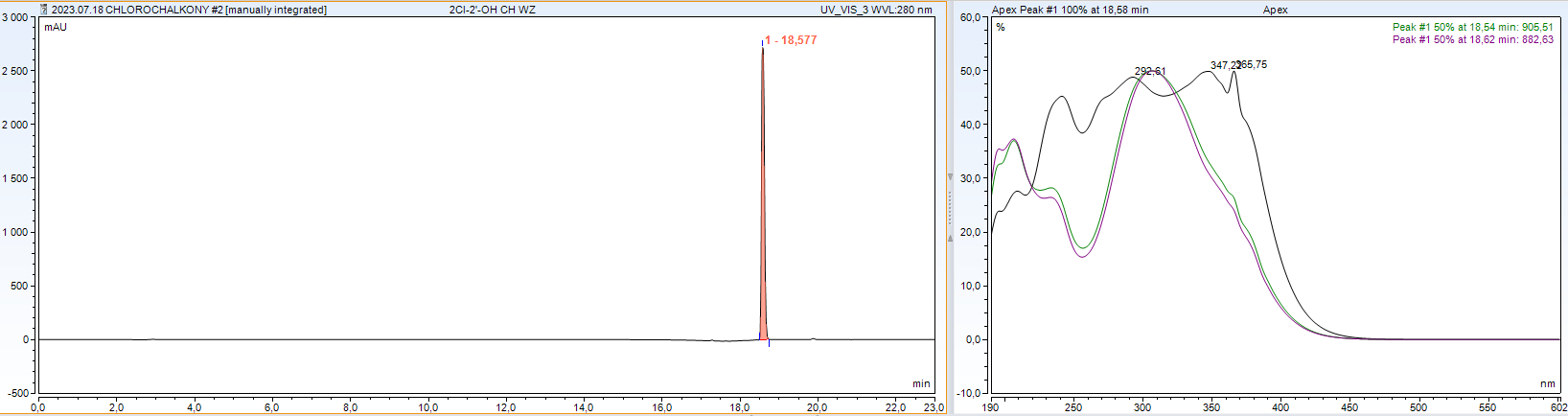


**Figure S2.** HPLC analysis of 2-chloro-2’-hydroxychalcone (**3**)


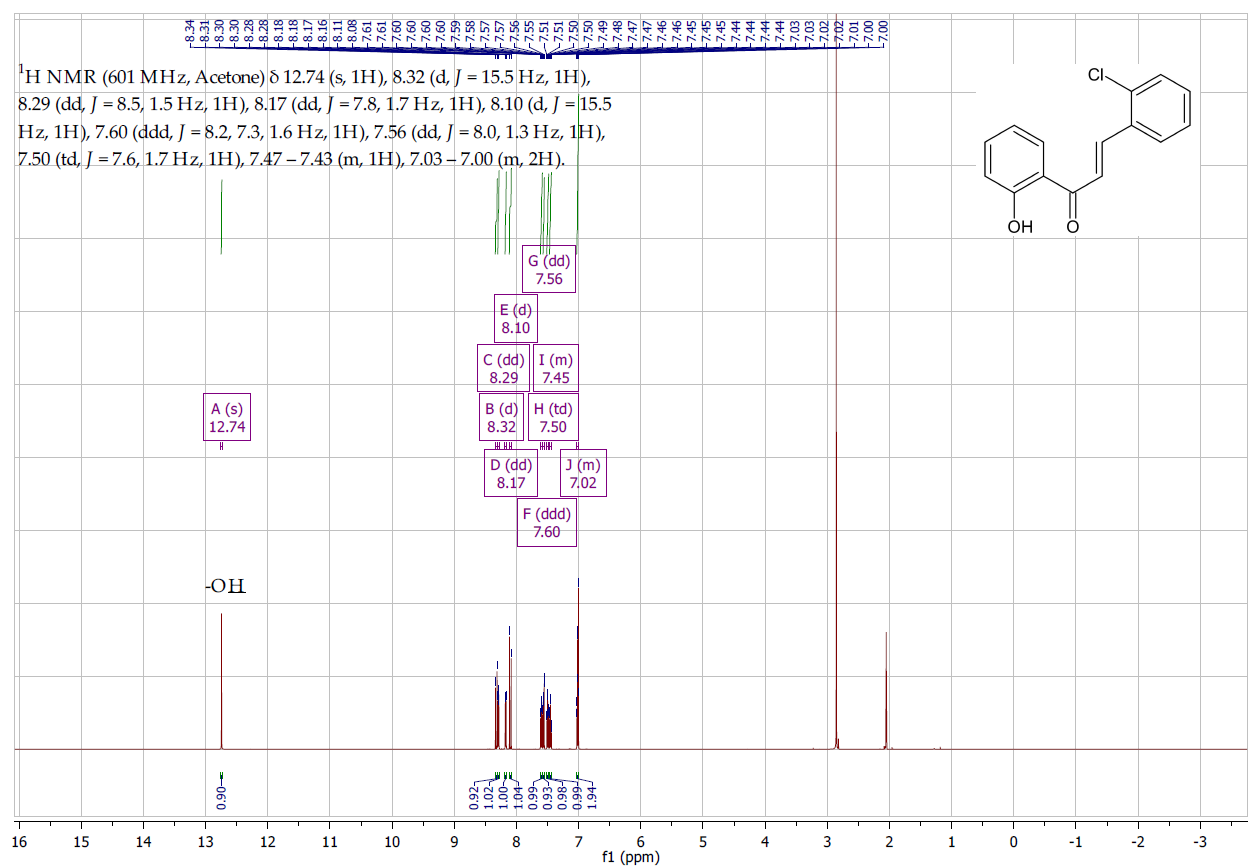


**Figure S3.** ^1^H NMR spectrum (*δ,* acetone-d6, 600 MHz) of 2-chloro-2’-hydroxychalcone (**3**)


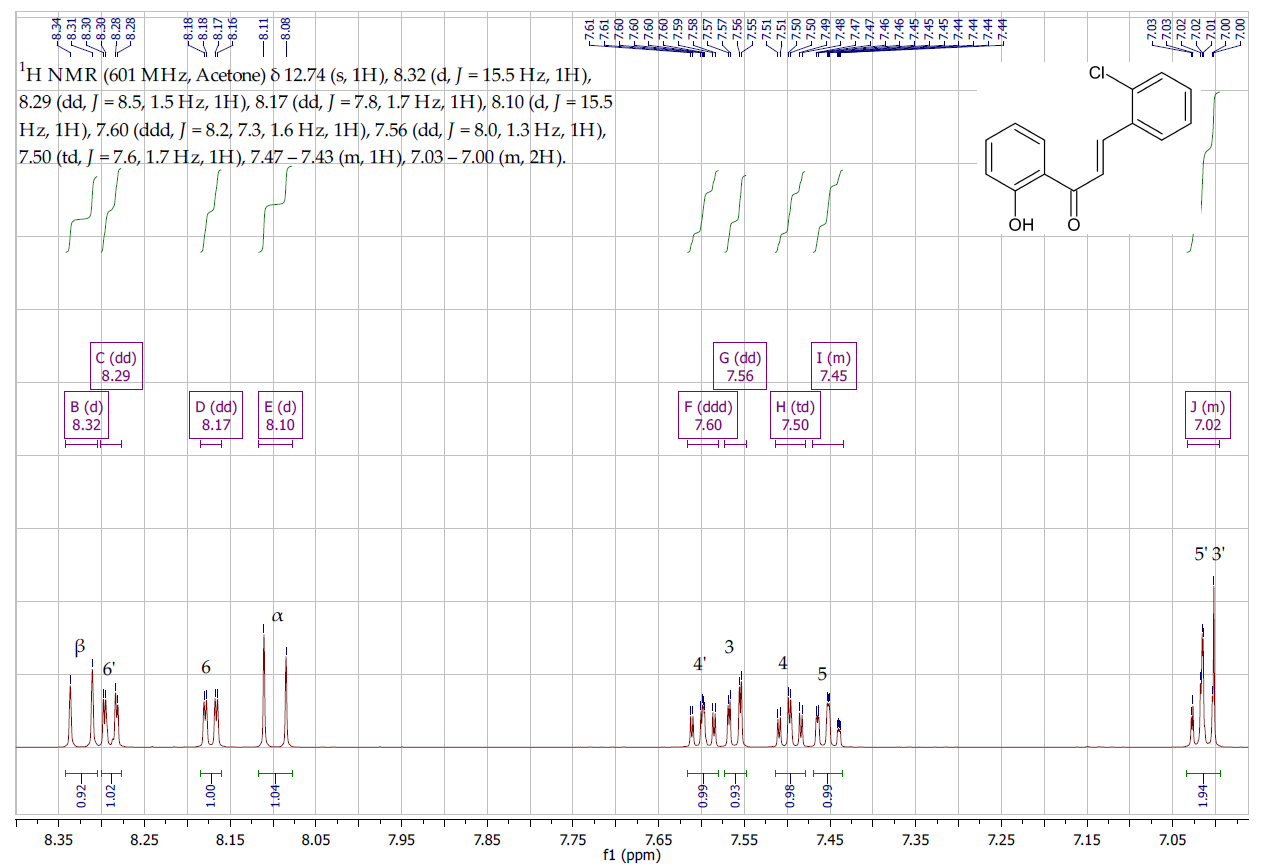


**Figure S4** ^1^H NMR spectrum expansion (*δ,* acetone-d6, 600 MHz) of 2-chloro-2’-hydroxychalcone (**3**)


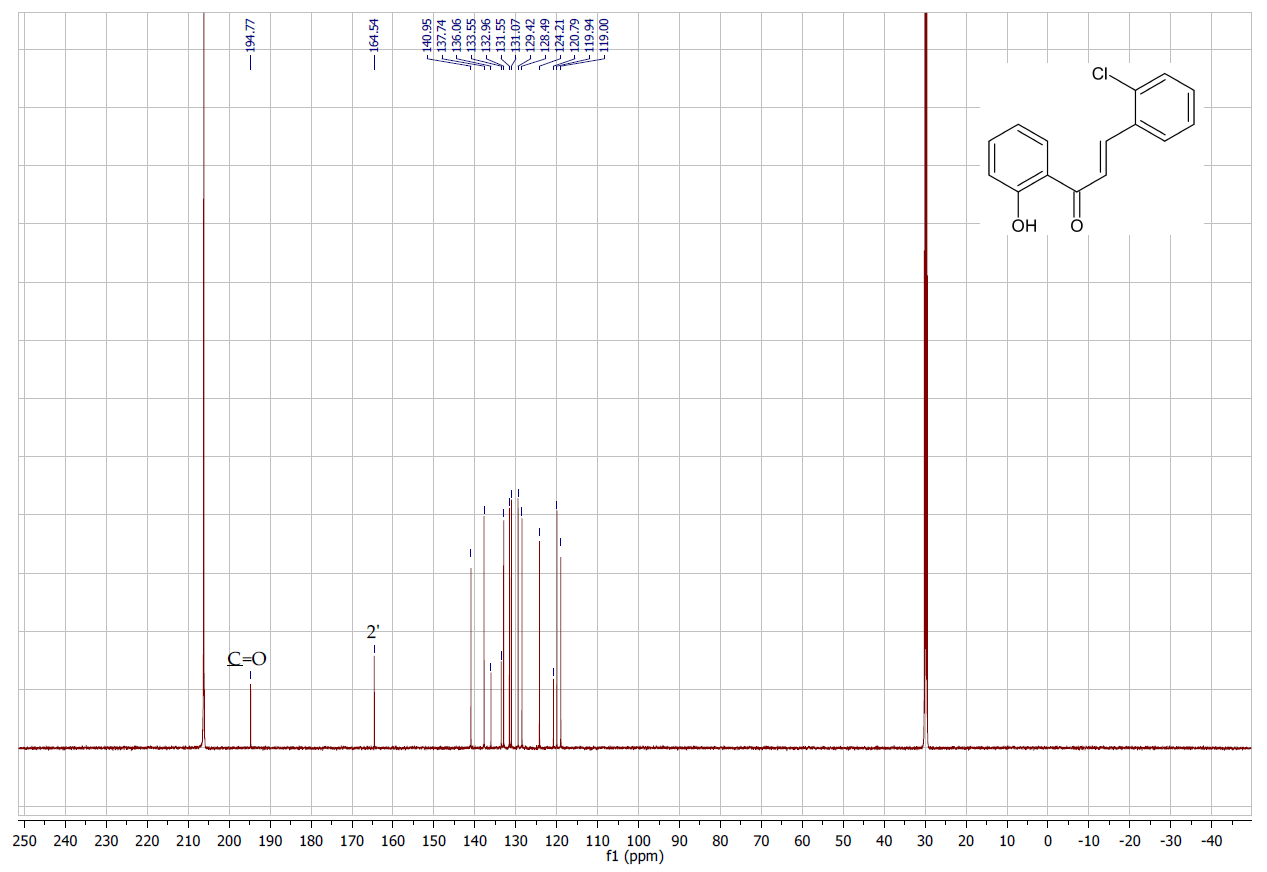


**Figure S5.** ^13^C NMR spectrum (*δ,* acetone-d6, 151 MHz) of 2-chloro-2’-hydroxychalcone (**3**)


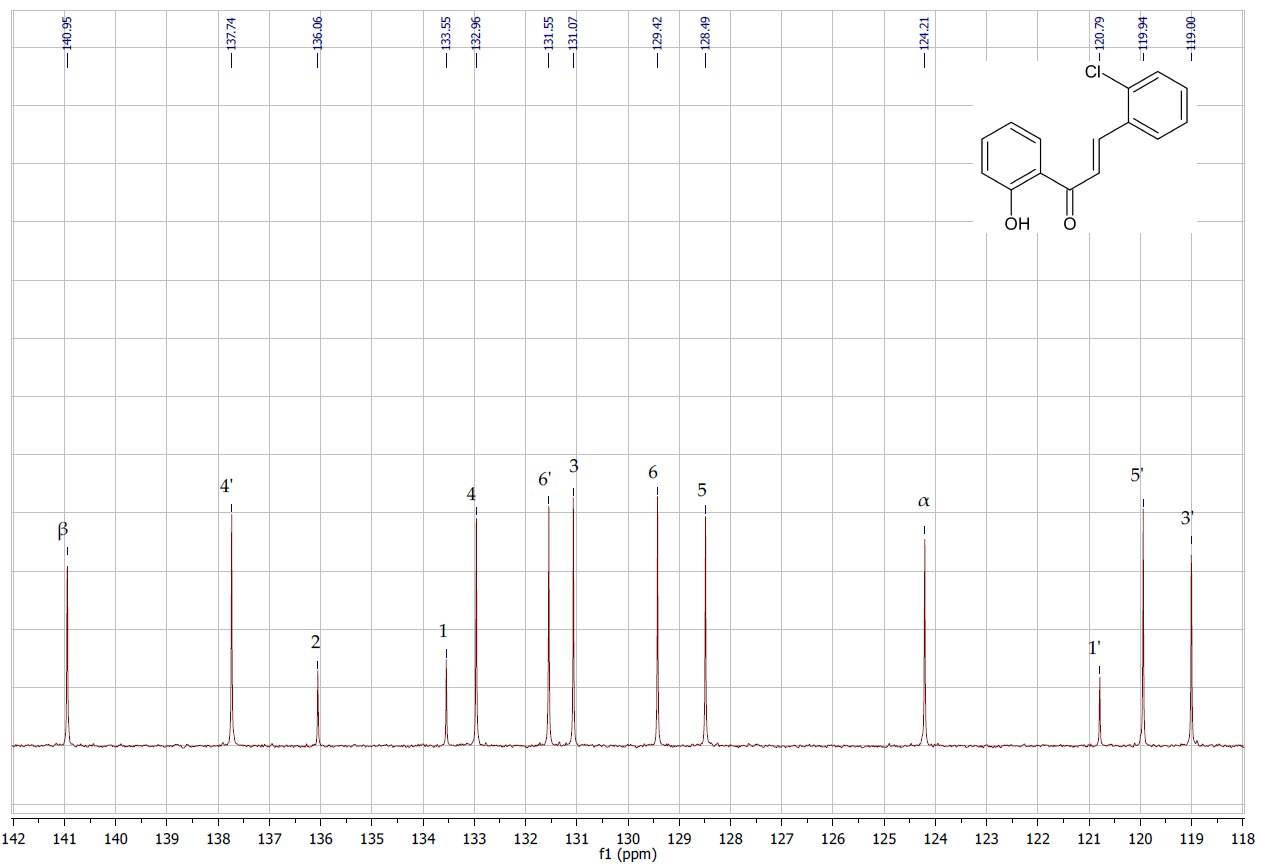


**Figure S6.** ^13^C NMR spectrum expansion (*δ,* acetone-d6, 151 MHz) of 2-chloro-2’-hydroxychalcone (**3**)


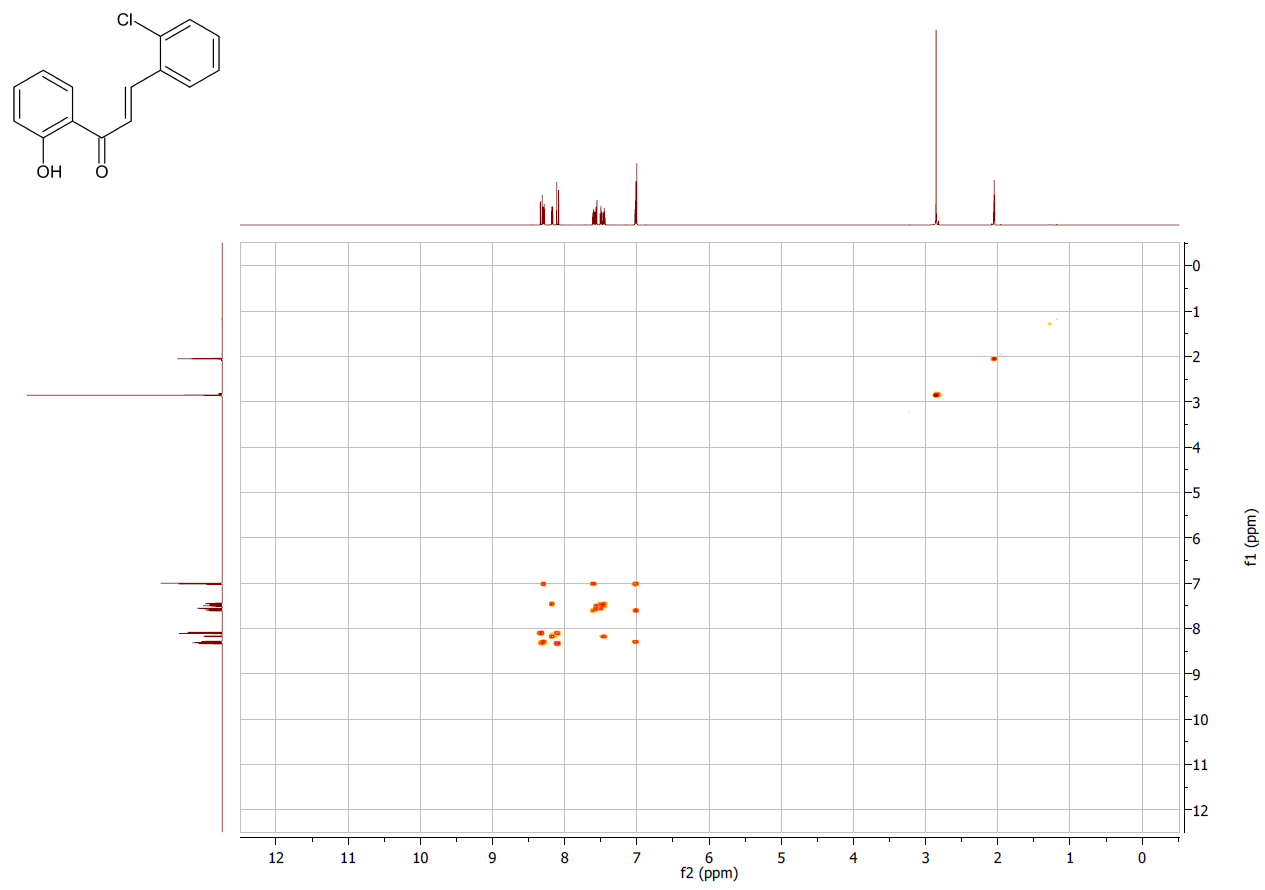


**Figure S7.** COSY contour map – ^1^H x ^1^H of 2-chloro-2’-hydroxychalcone (**3**)


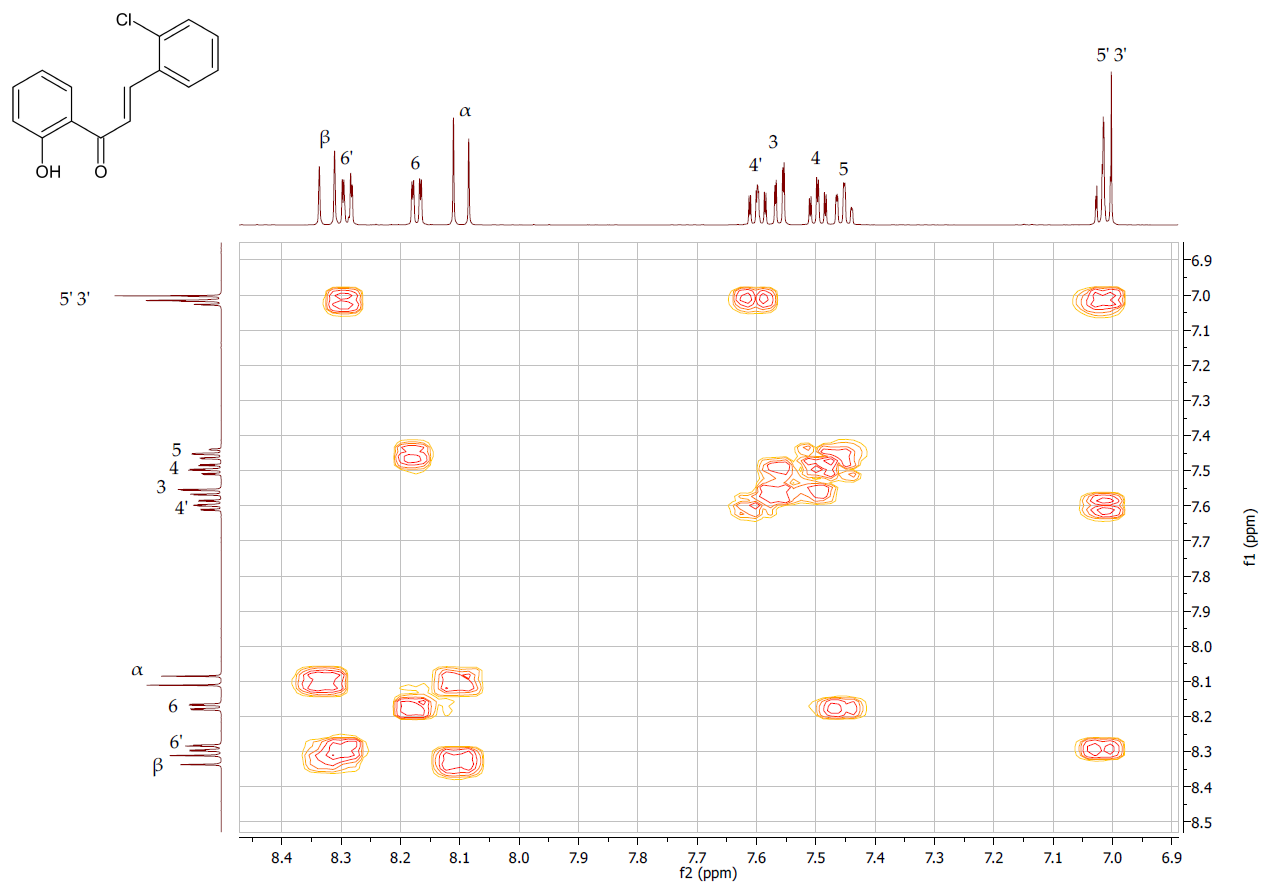


**Figure S8.** COSY contour map – ^1^H x ^1^H expansion of 2-chloro-2’-hydroxychalcone (**3**)


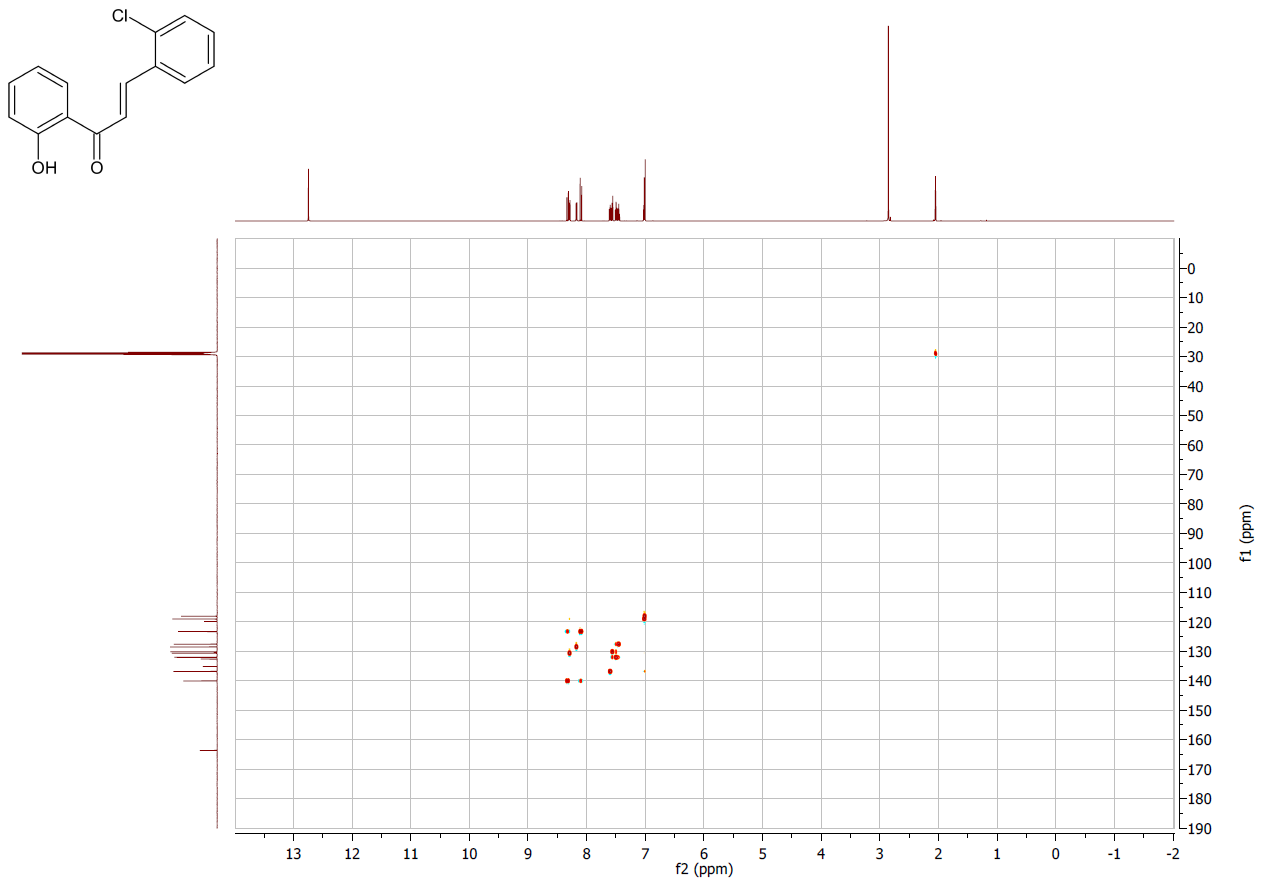


**Figure S9.** HMQC contour map – ^1^H x ^13^C of 2-chloro-2’-hydroxychalcone (**3**)


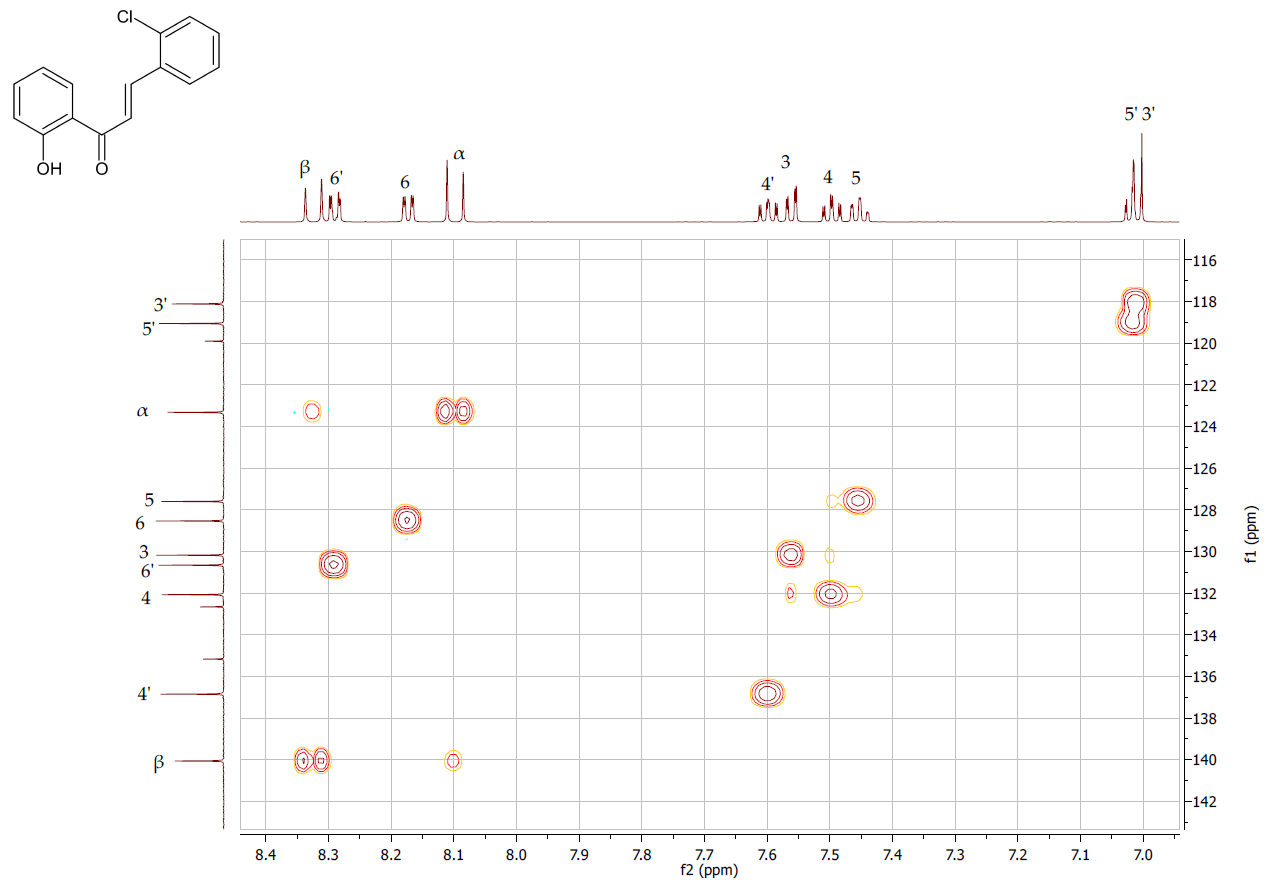


**Figure S10.** HMQC contour map – ^1^H x ^13^C expansion of 2-chloro-2’-hydroxychalcone (**3**)


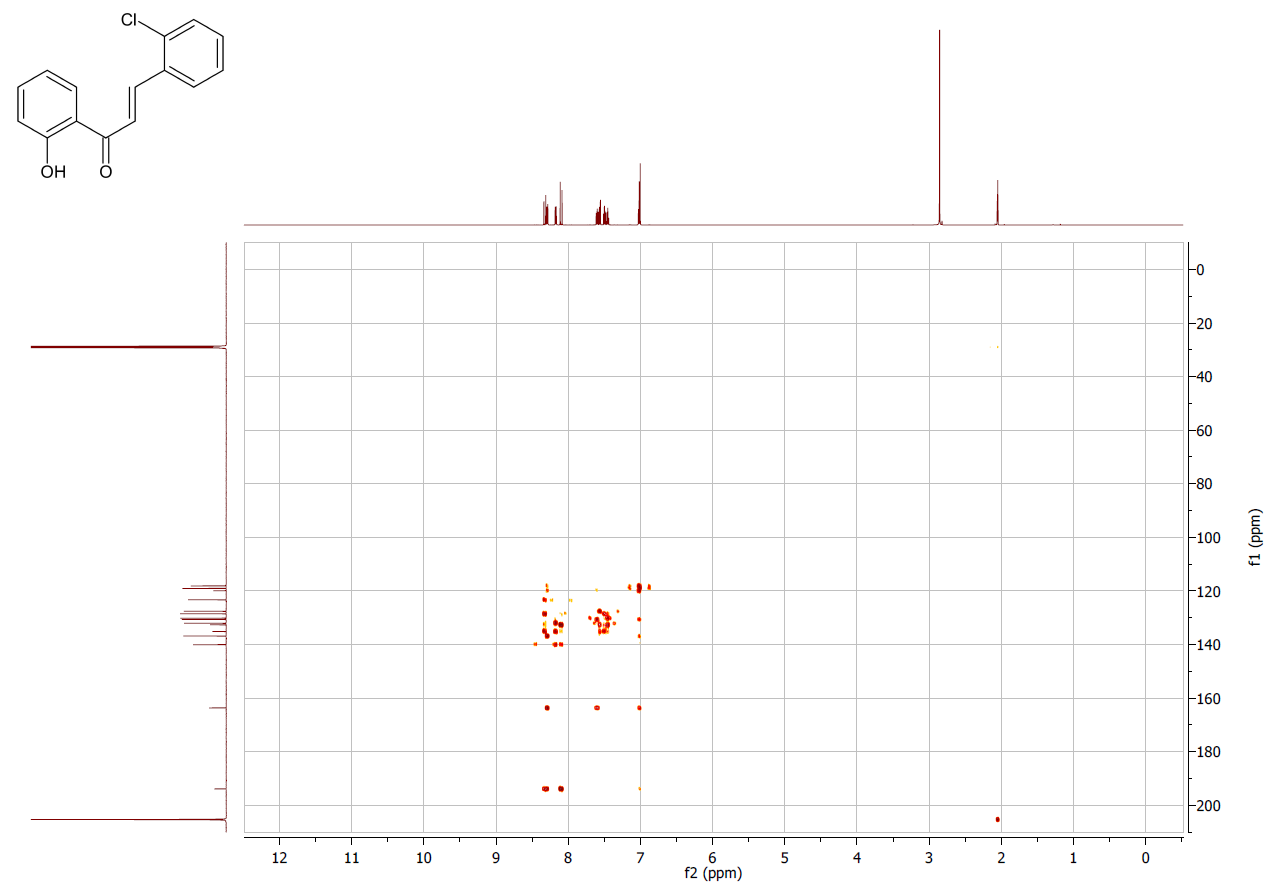


**Figure S11.** HMBC contour map – ^1^H x ^13^C of 2-chloro-2’-hydroxychalcone (**3**)


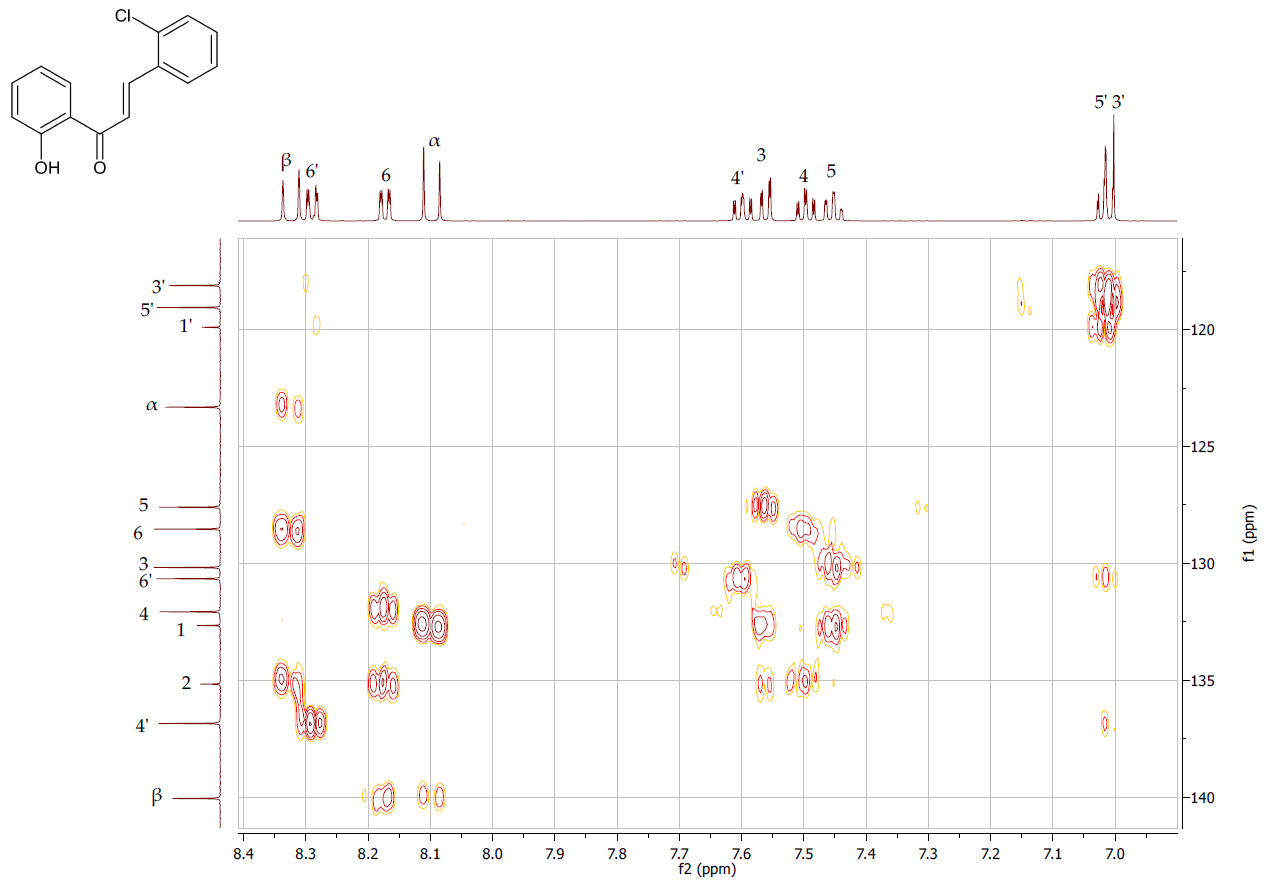


**Figure S12.** HMBC contour map – ^1^H x ^13^C expansion of 2-chloro-2’-hydroxychalcone (**3**)


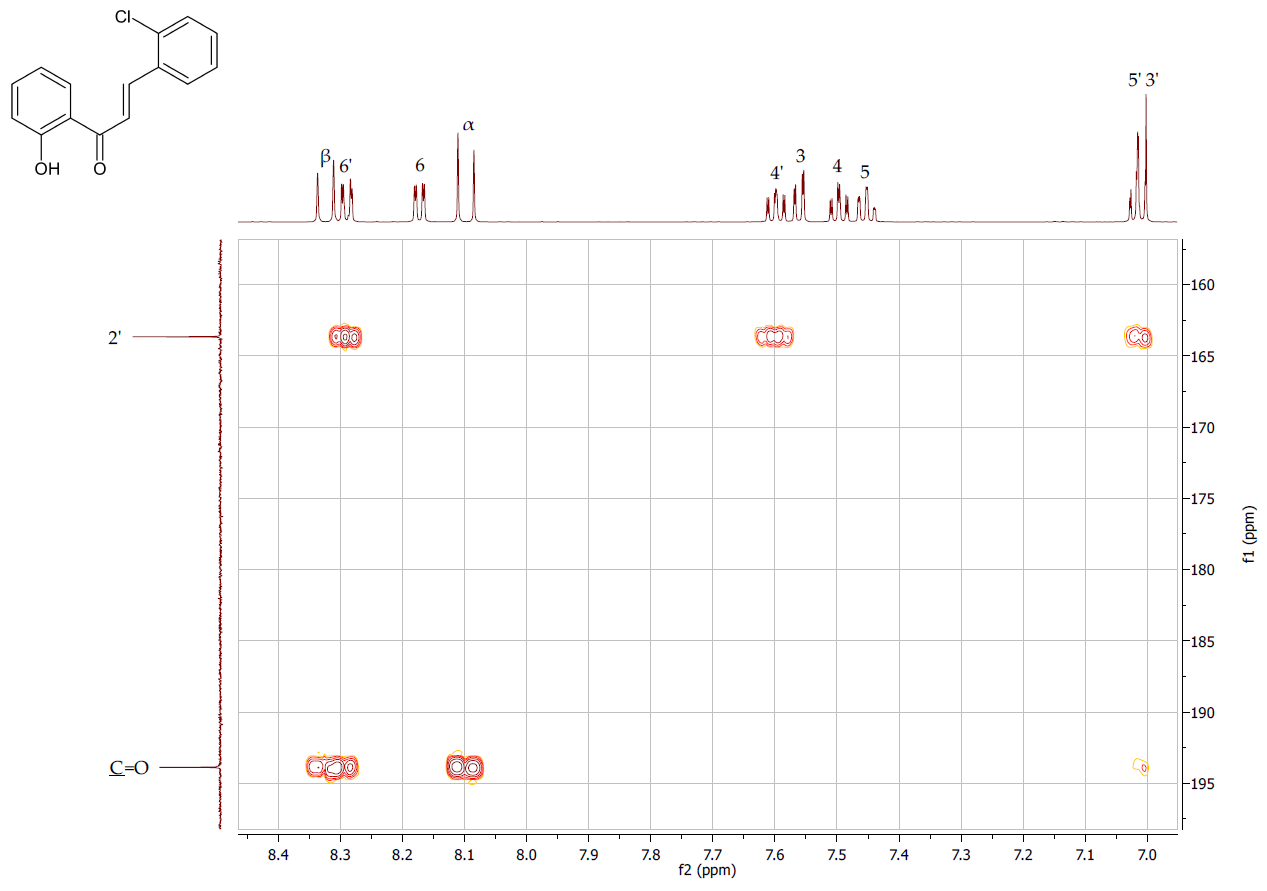


**Figure S13.** HMBC contour map – ^1^H x ^13^C expansion of 2-chloro-2’-hydroxychalcone (**3**)


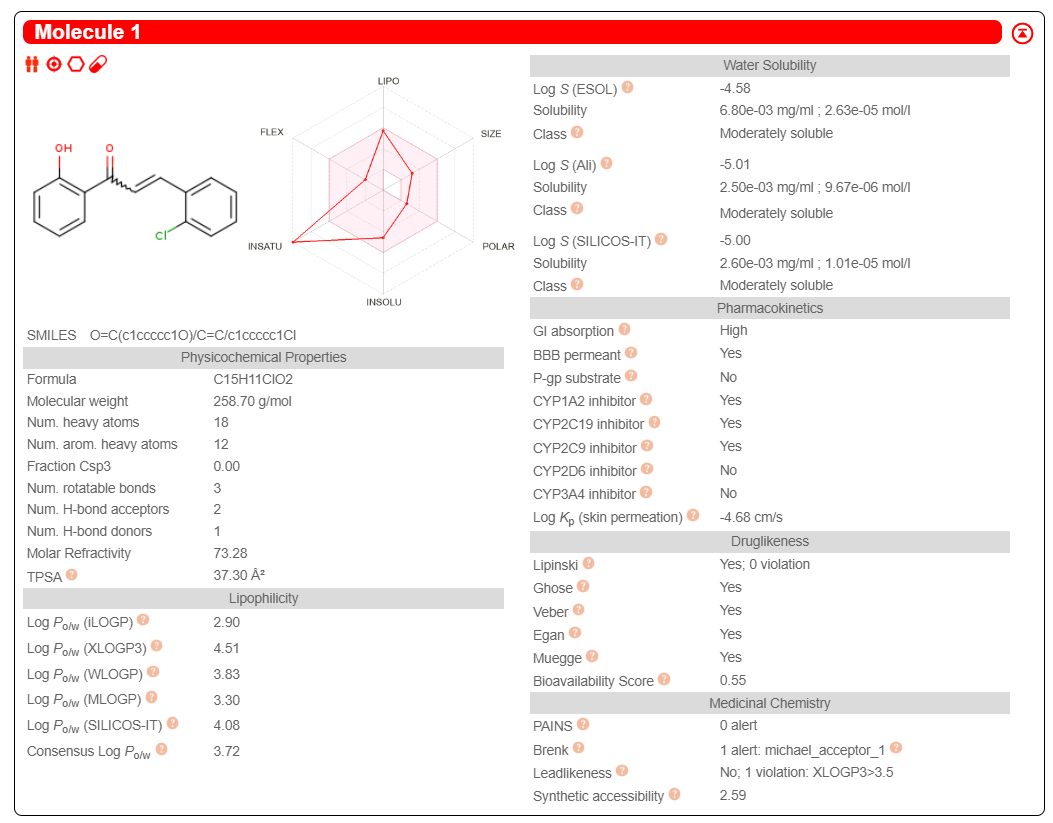


**Figure S14.** 2-Chloro-2’-hydroxychalcone (**3**) physicochemical and ADME parameters prediction using the SwissADME modelling


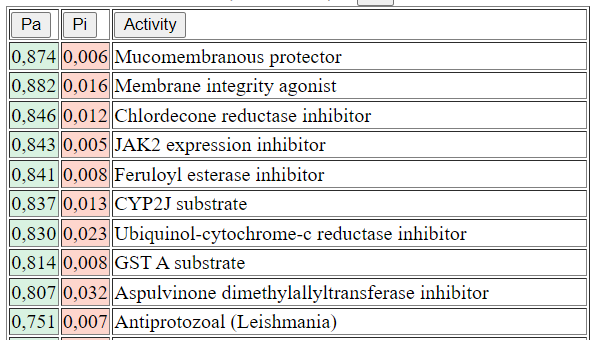


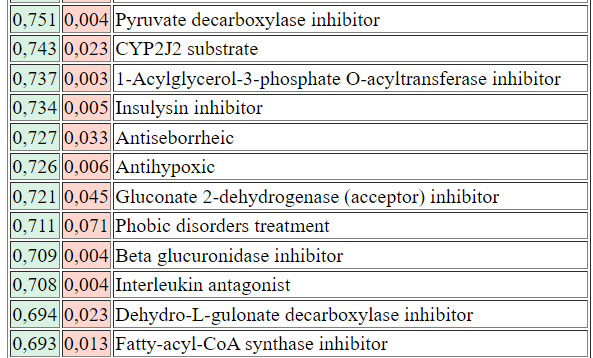


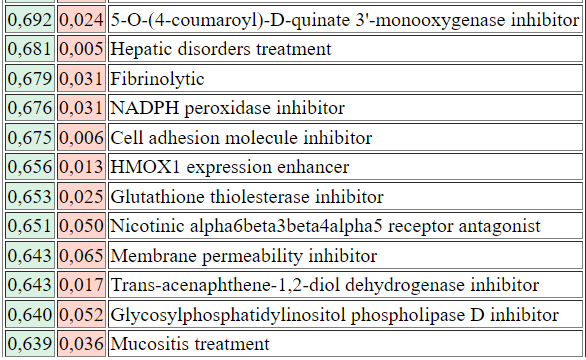


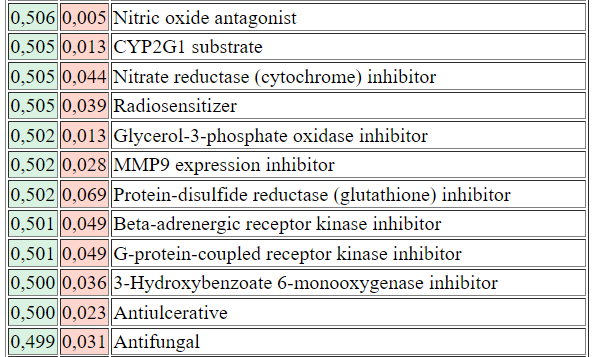


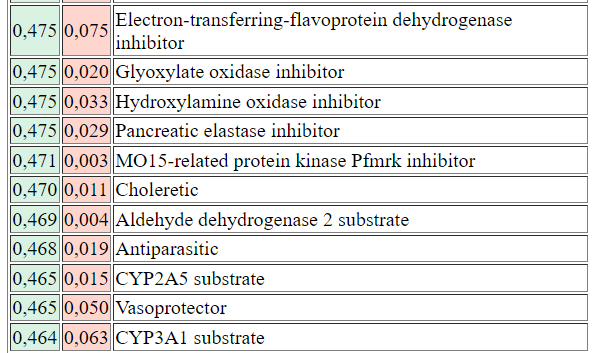


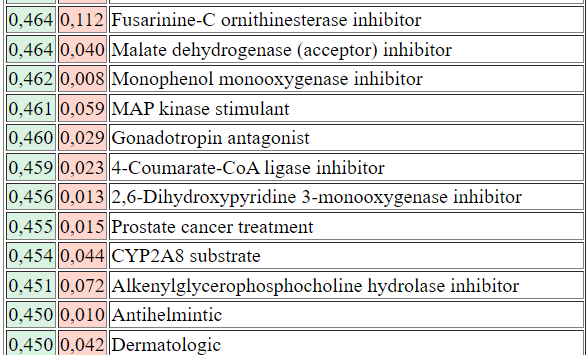


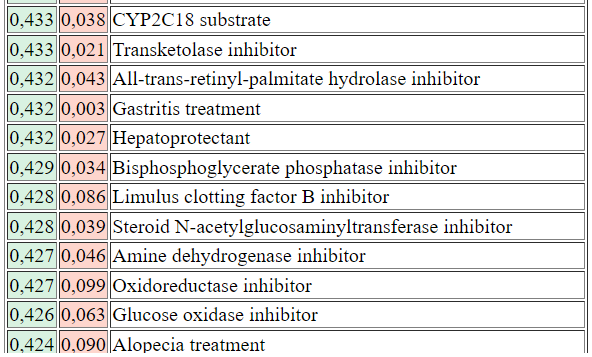


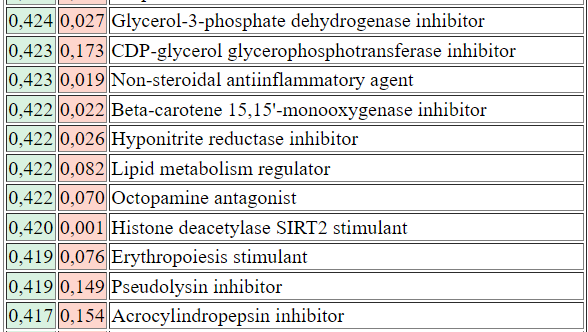


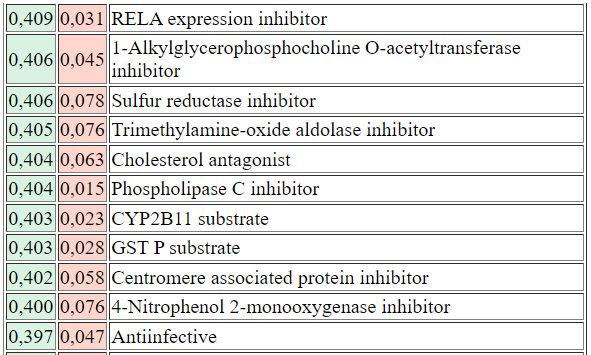


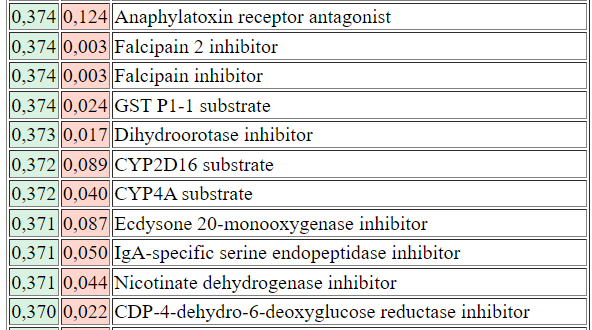


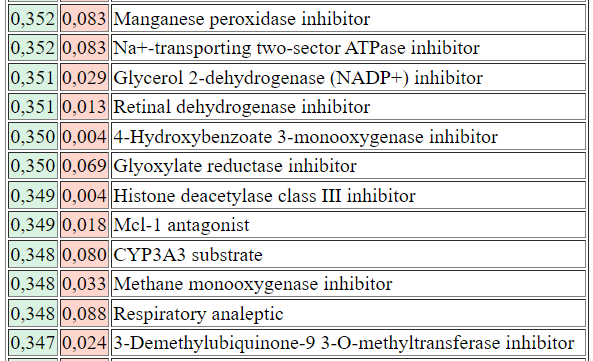


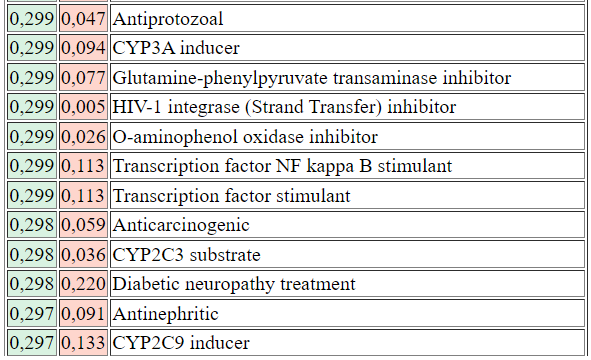


**Figure S15.** 2-Chloro-2’-hydroxychalcone (**3**) physicochemical biological activity prediction using the Way2Drug Pass online modelling


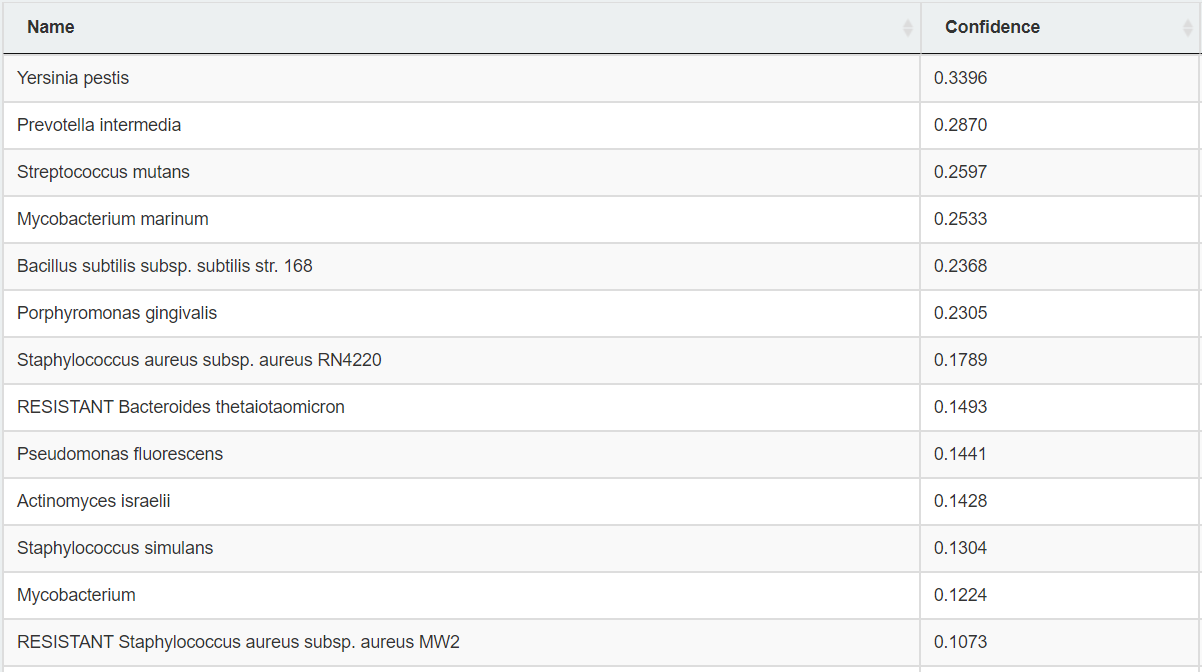


**Figure S16.** 2-Chloro-2’-hydroxychalcone (**3**) antibacterial activity prediction using the Way2Drug AntiBac-Pred modelling


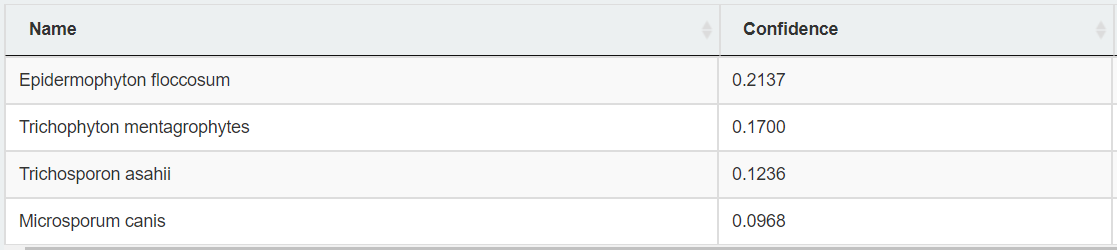


**Figure S17.** 2-Chloro-2’-hydroxychalcone (**3**) antifungal activity prediction using the Way2Drug AntiFun-Pred modelling


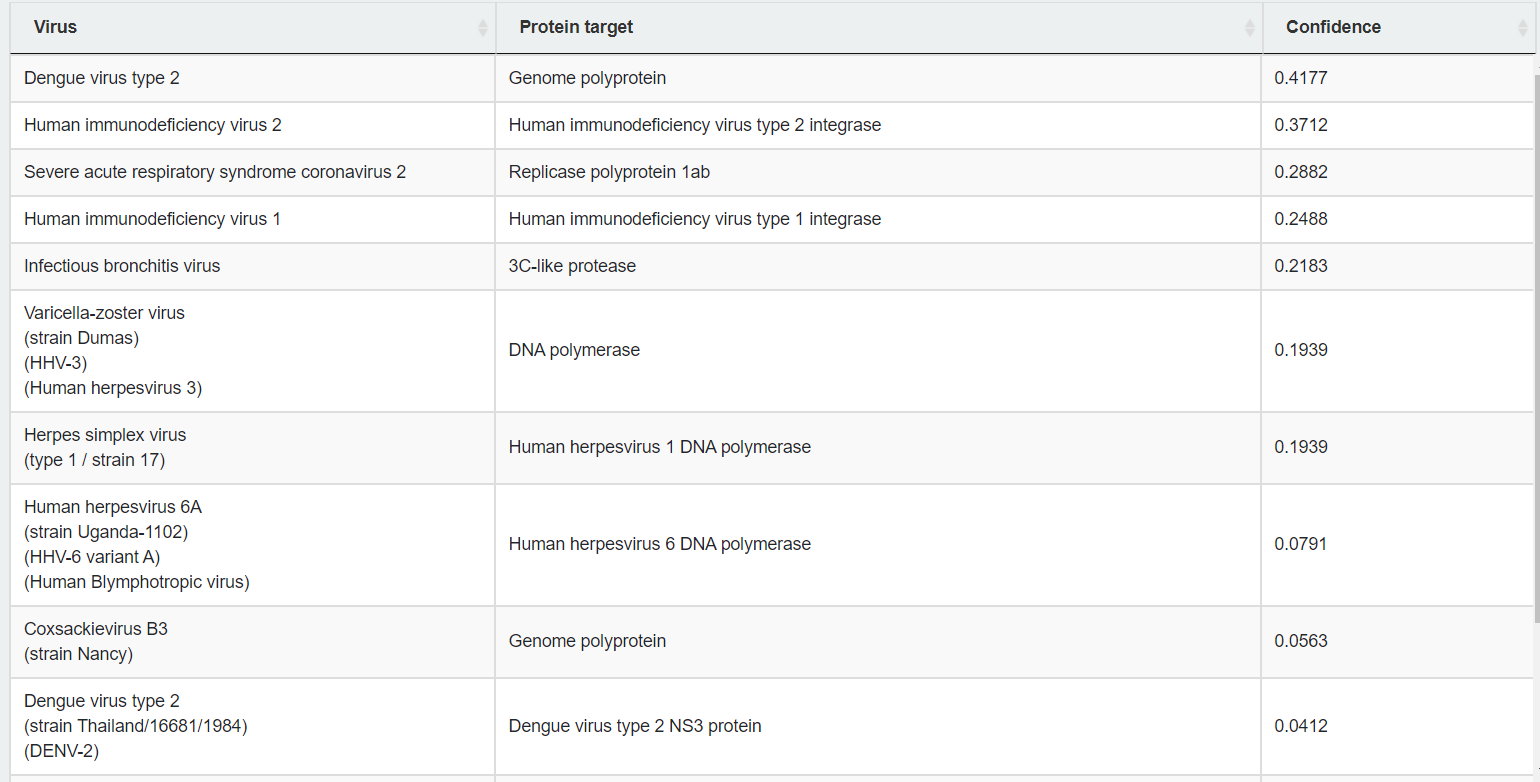


**Figure S18.** 2-Chloro-2’-hydroxychalcone (**3**) antiviral activity prediction using the Way2Drug AntiVir-Pred modelling


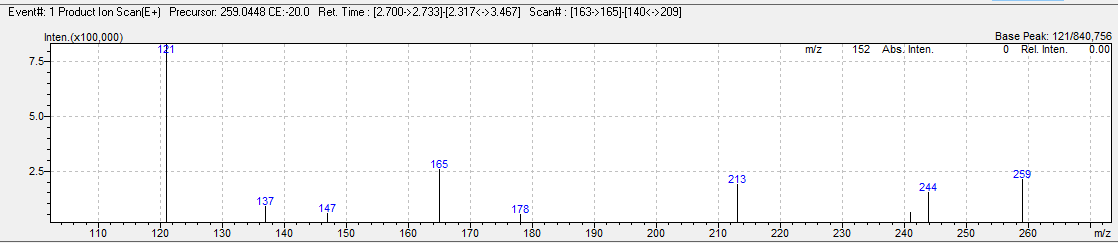


**Figure S19.** MS analysis of 3-chloro-2’-hydroxychalcone (**5**)


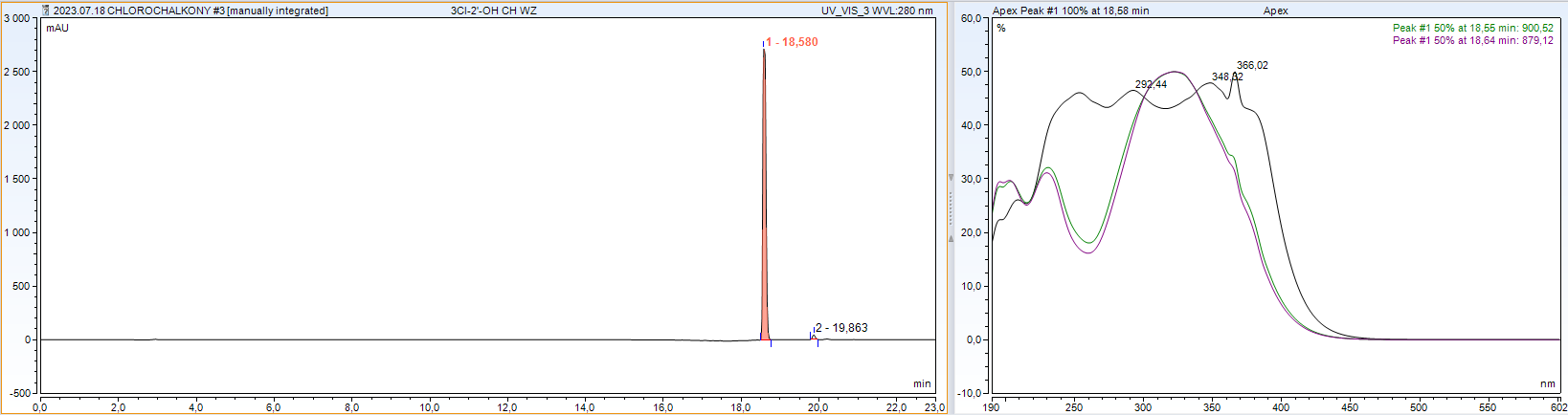


**Figure S20.** HPLC analysis of 3-chloro-2’-hydroxychalcone (**5**)


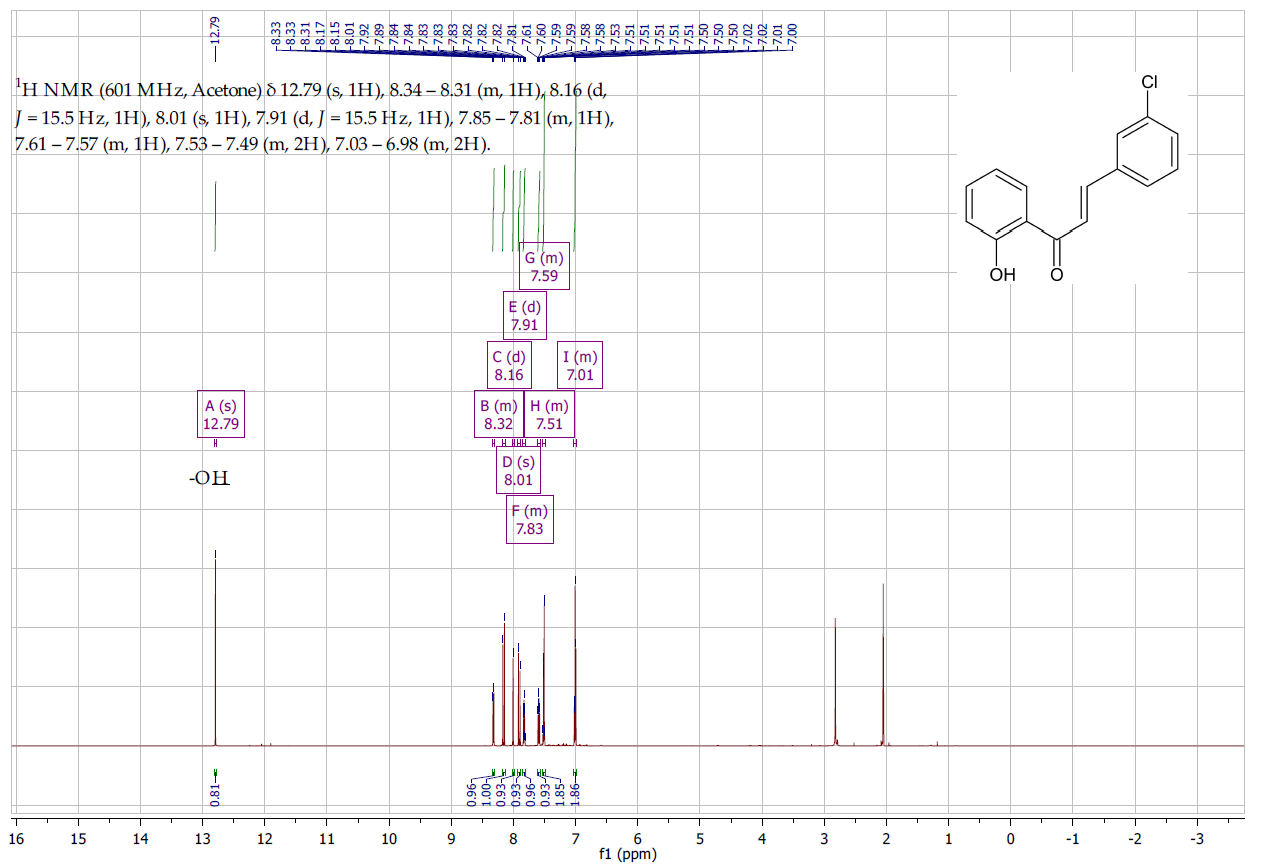


**Figure S21** ^1^H NMR spectrum (*δ,* acetone-d6, 600 MHz) of 3-chloro-2’-hydroxychalcone (**5**)


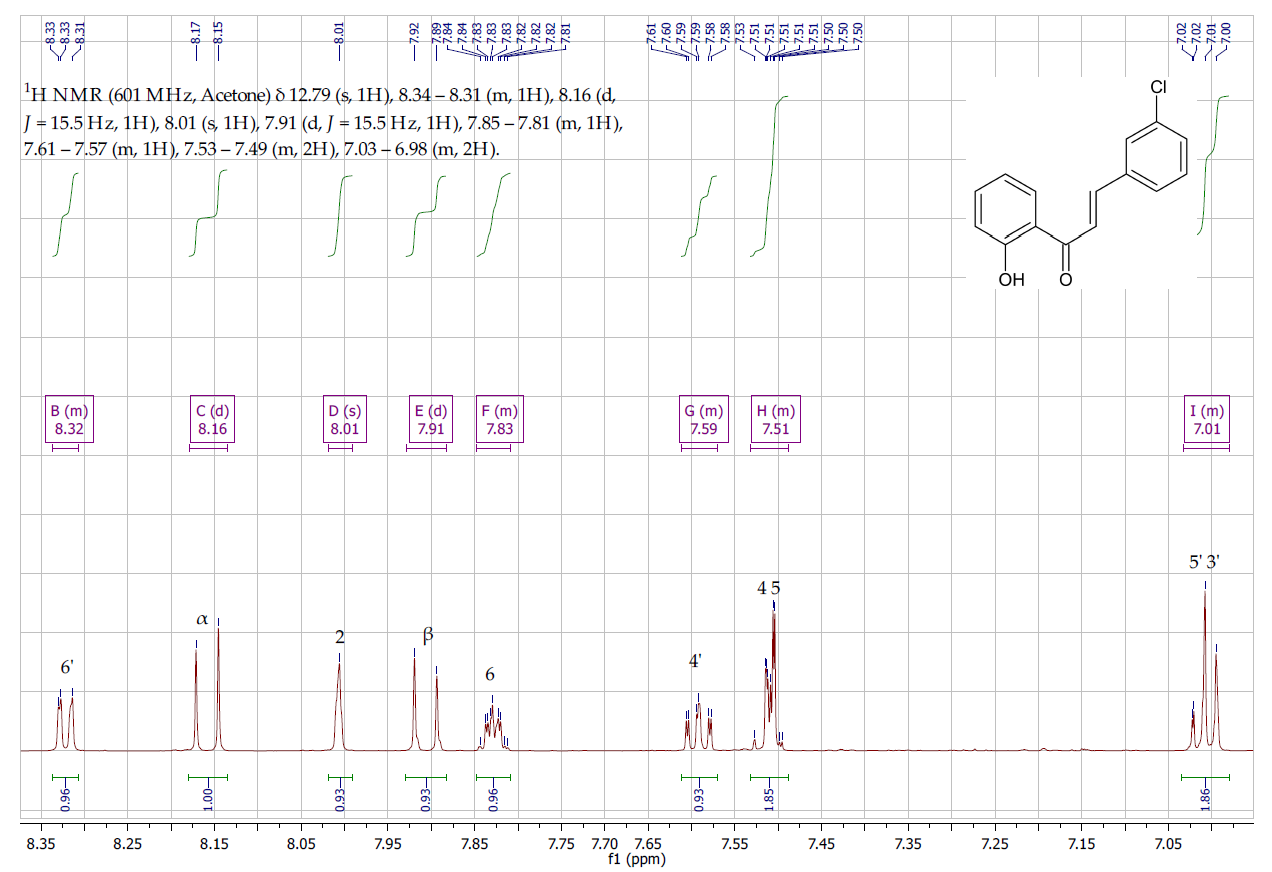


**Figure S22** ^1^H NMR spectrum expansion (*δ,* acetone-d6, 600 MHz) of 3-chloro-2’-hydroxychalcone (**5**)


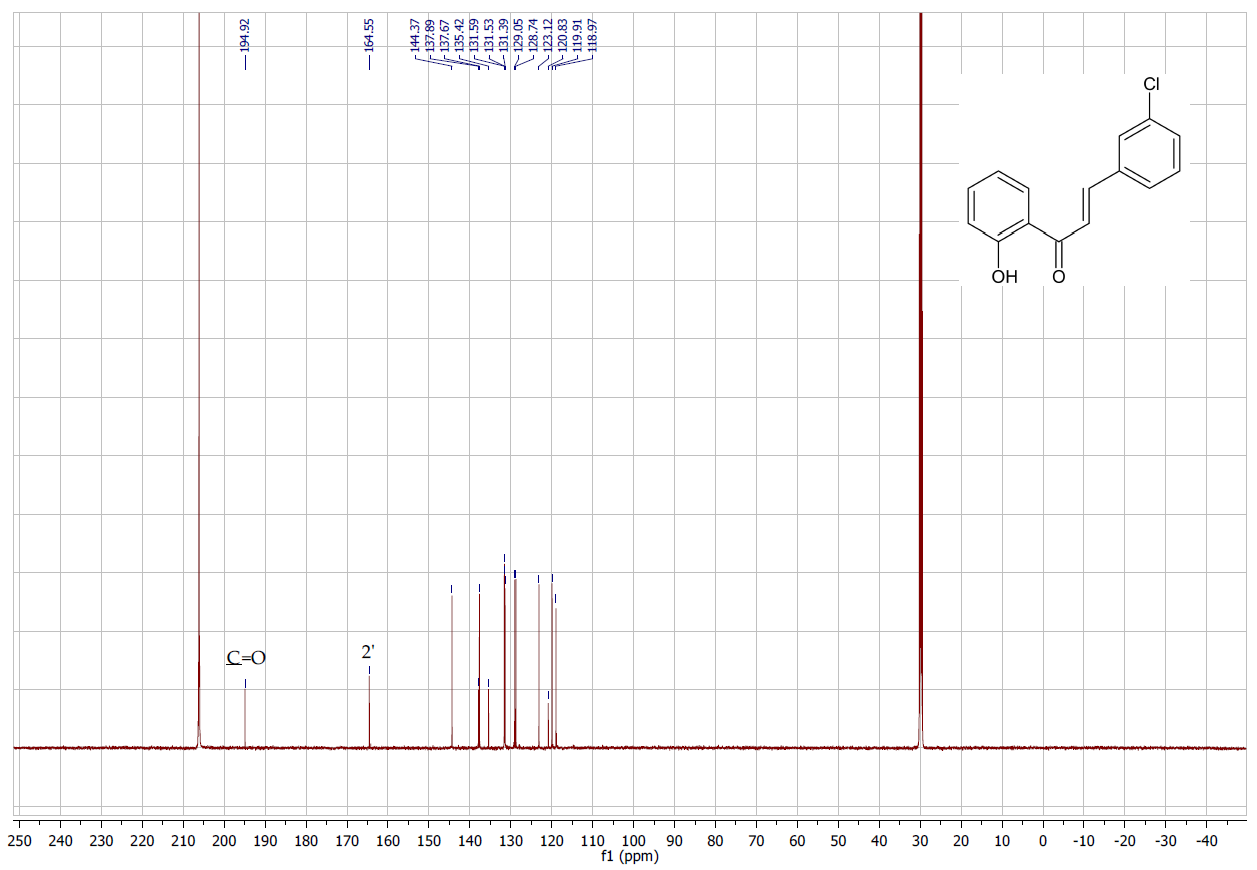


**Figure S23.** ^13^C NMR spectrum (*δ,* acetone-d6, 151 MHz) of 3-chloro-2’-hydroxychalcone (**5**)


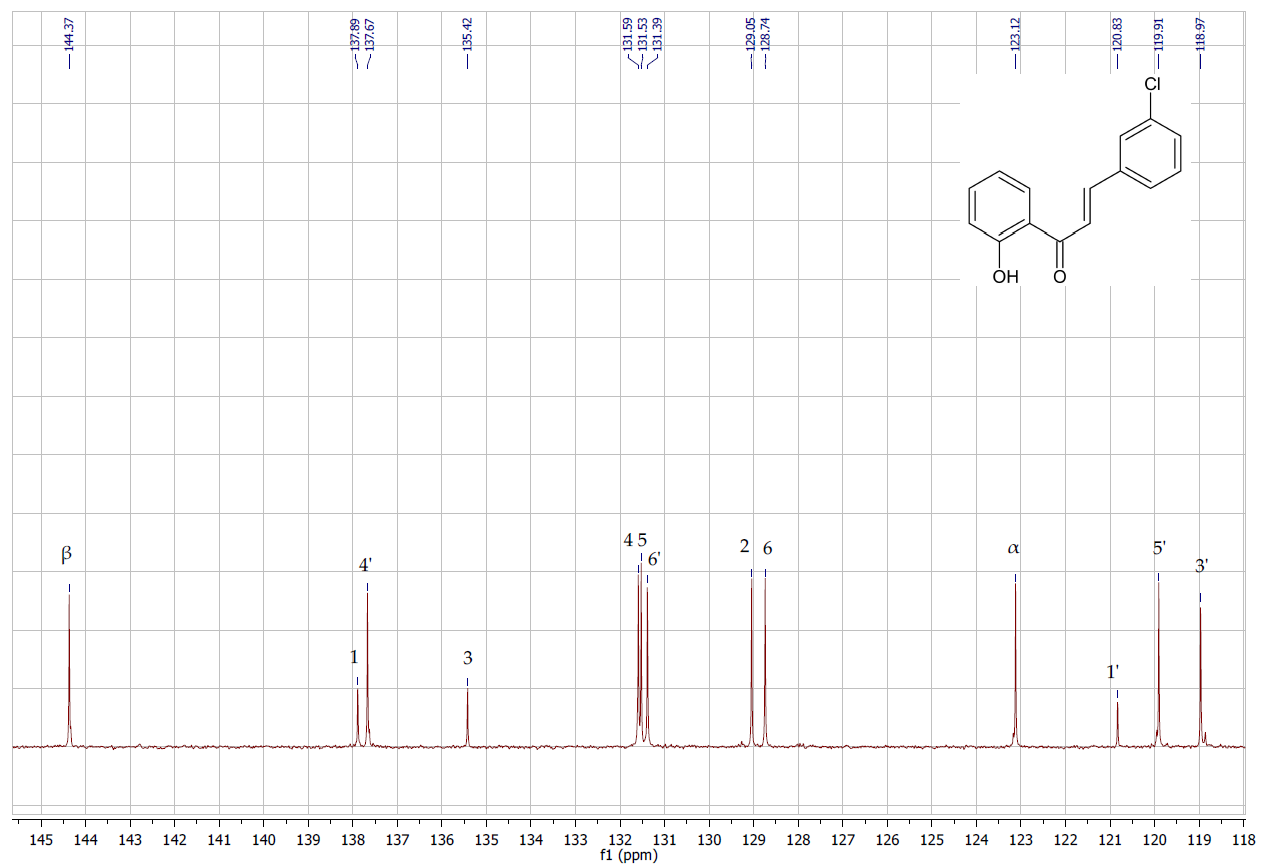


**Figure S24.** ^13^C NMR spectrum expansion (*δ,* acetone-d6, 151 MHz) of 3-chloro-2’-hydroxychalcone (**5**)


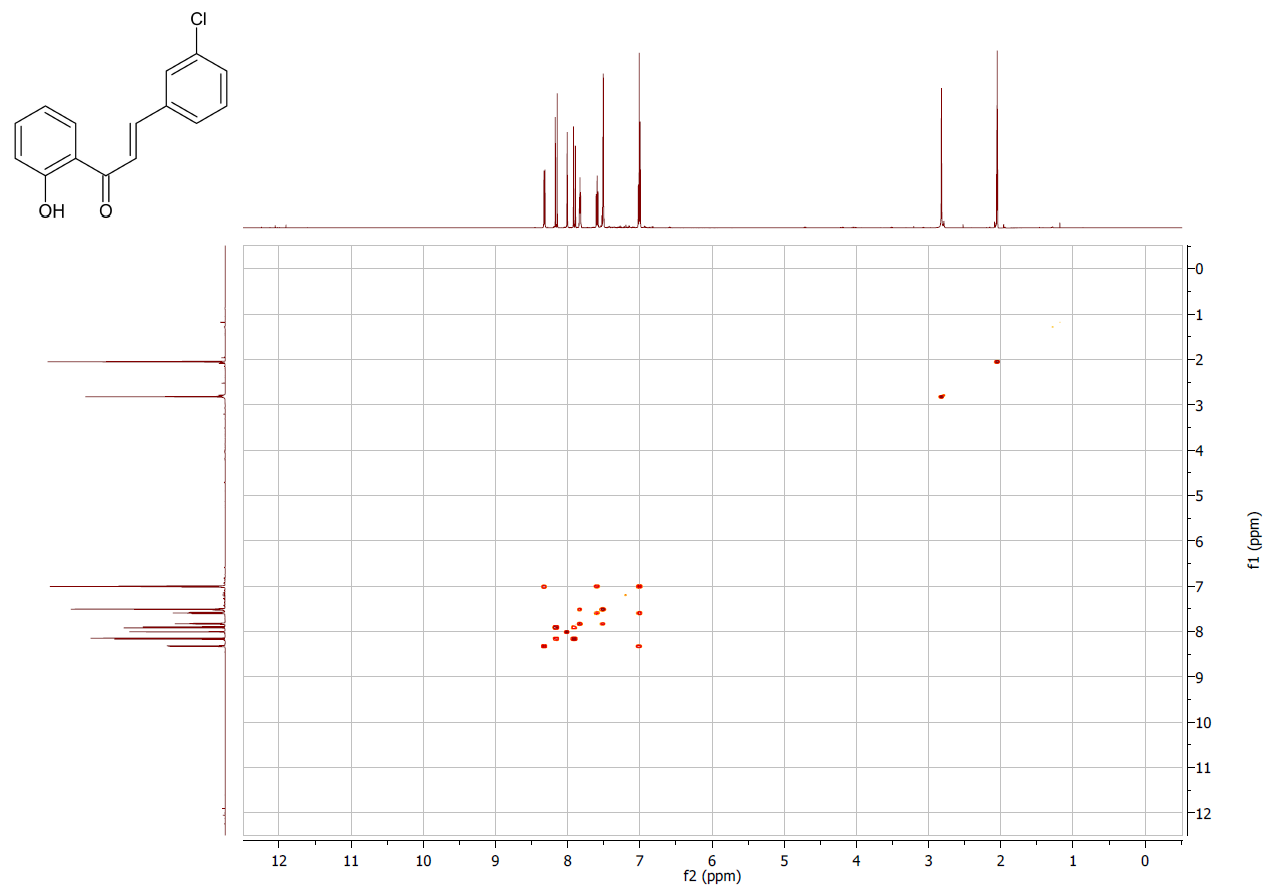


**Figure S25.** COSY contour map – ^1^H x ^1^H of 3-chloro-2’-hydroxychalcone (**5**)


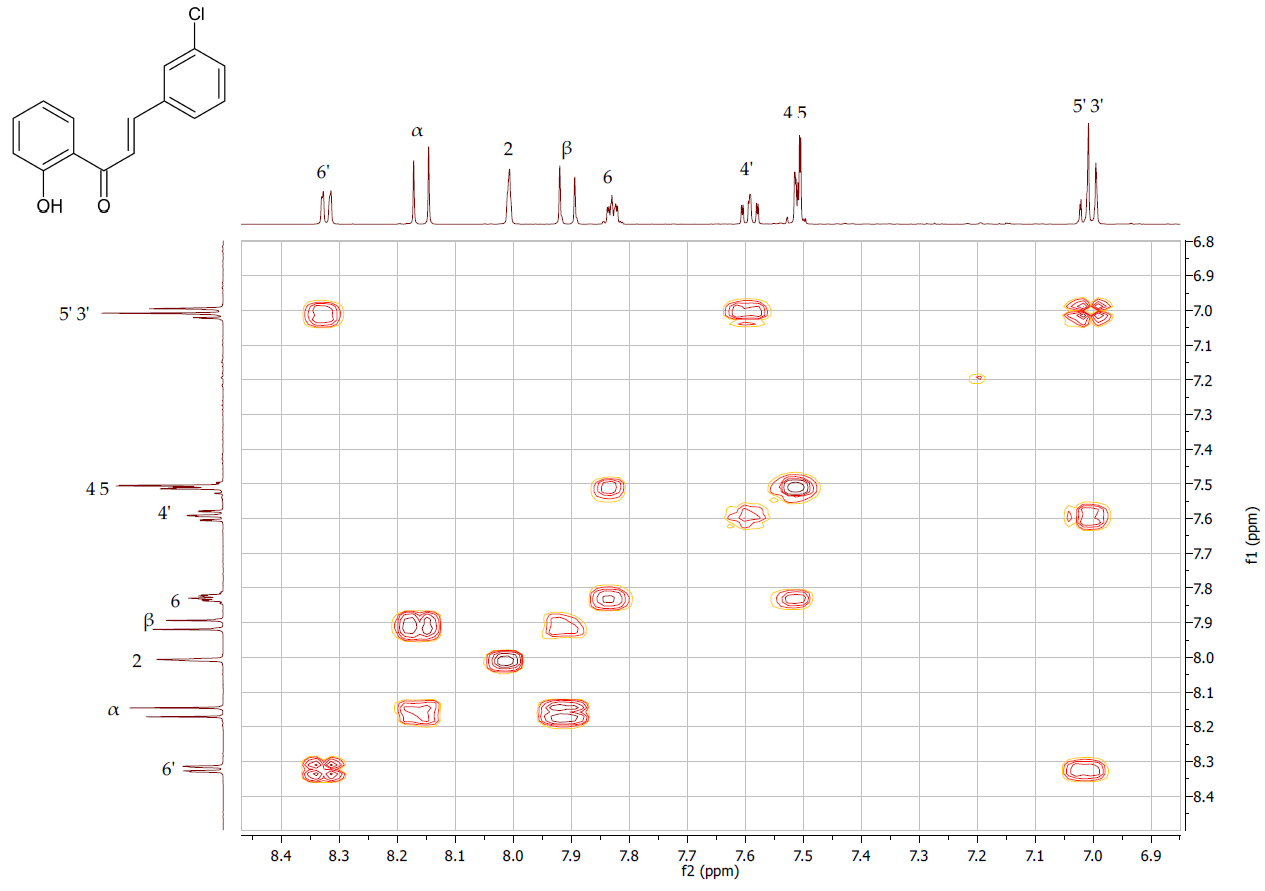


**Figure S26.** COSY contour map – ^1^H x ^1^H expansion of 3-chloro-2’-hydroxychalcone (**5**)


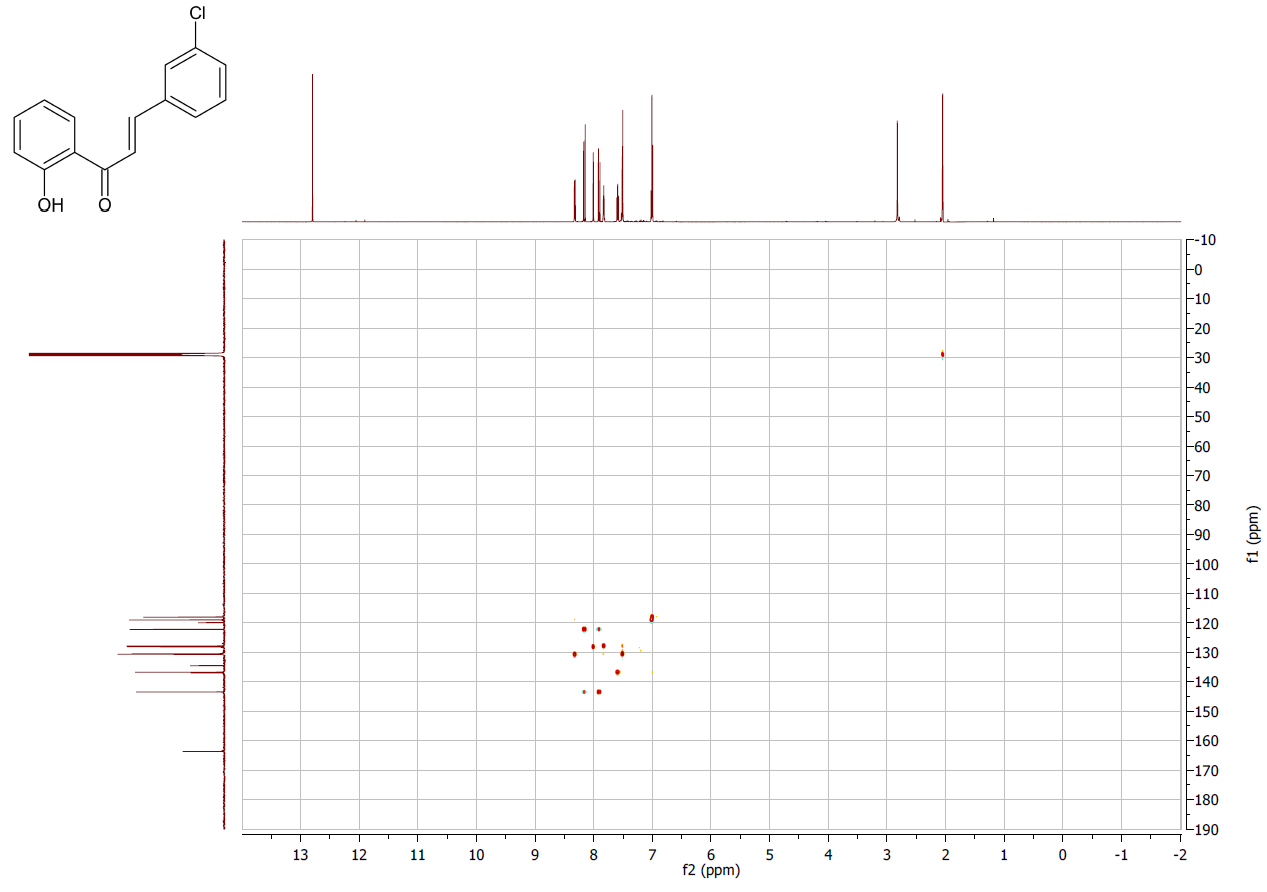


**Figure S27.** HMQC contour map – ^1^H x ^13^C of 3-chloro-2’-hydroxychalcone (**5**)


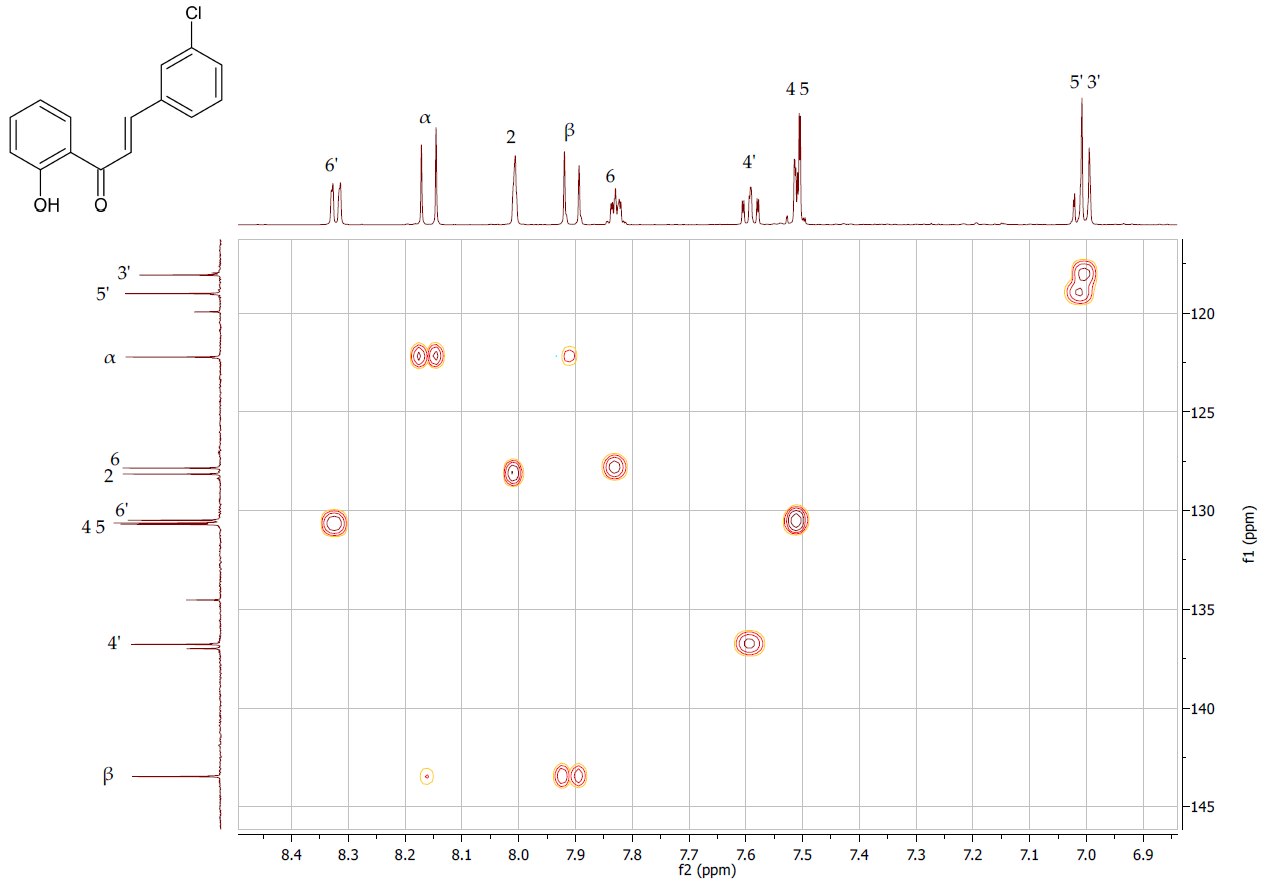


**Figure S28.** HMQC contour map – ^1^H x ^13^C expansion of 3-chloro-2’-hydroxychalcone (**5**)


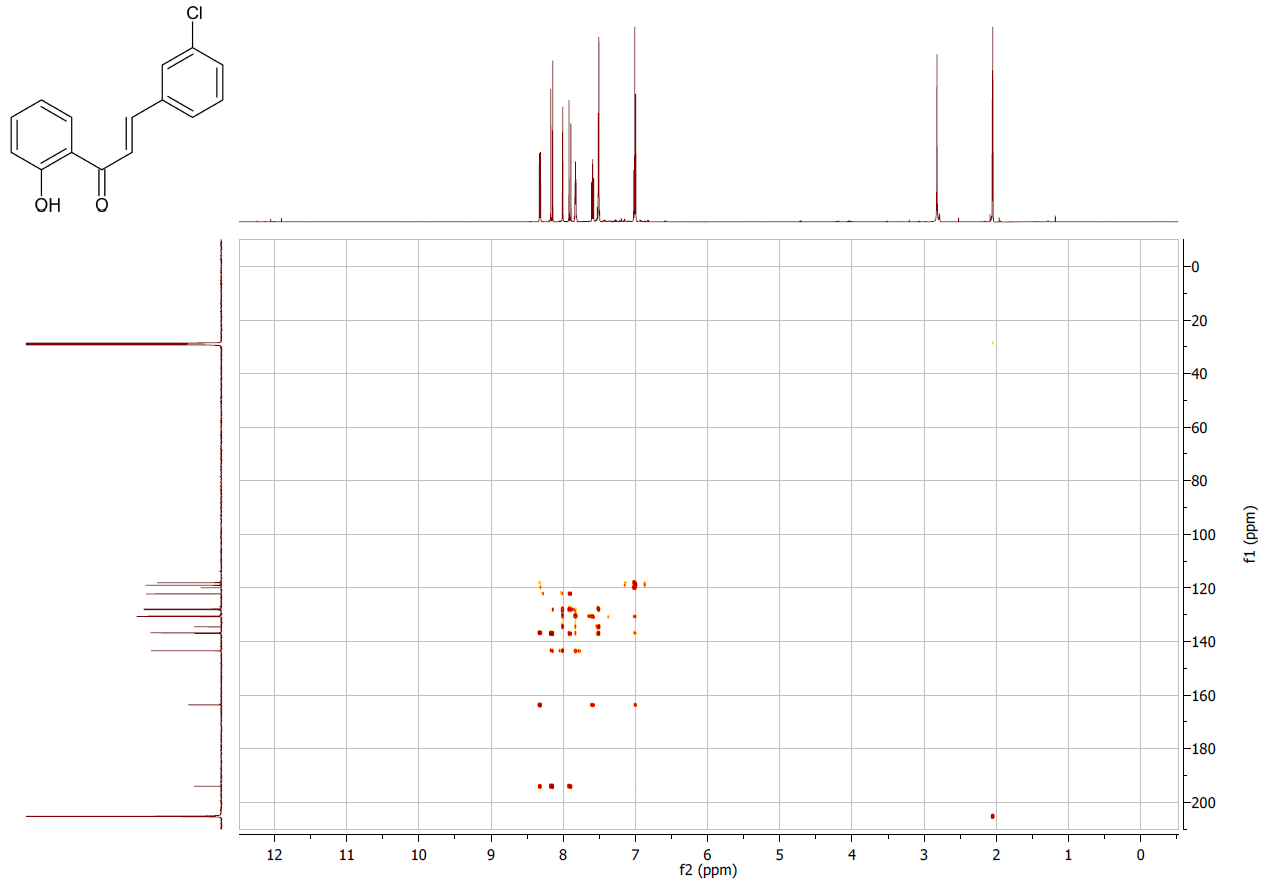


**Figure S29.** HMBC contour map – ^1^H x ^13^C of 3-chloro-2’-hydroxychalcone (**5**)


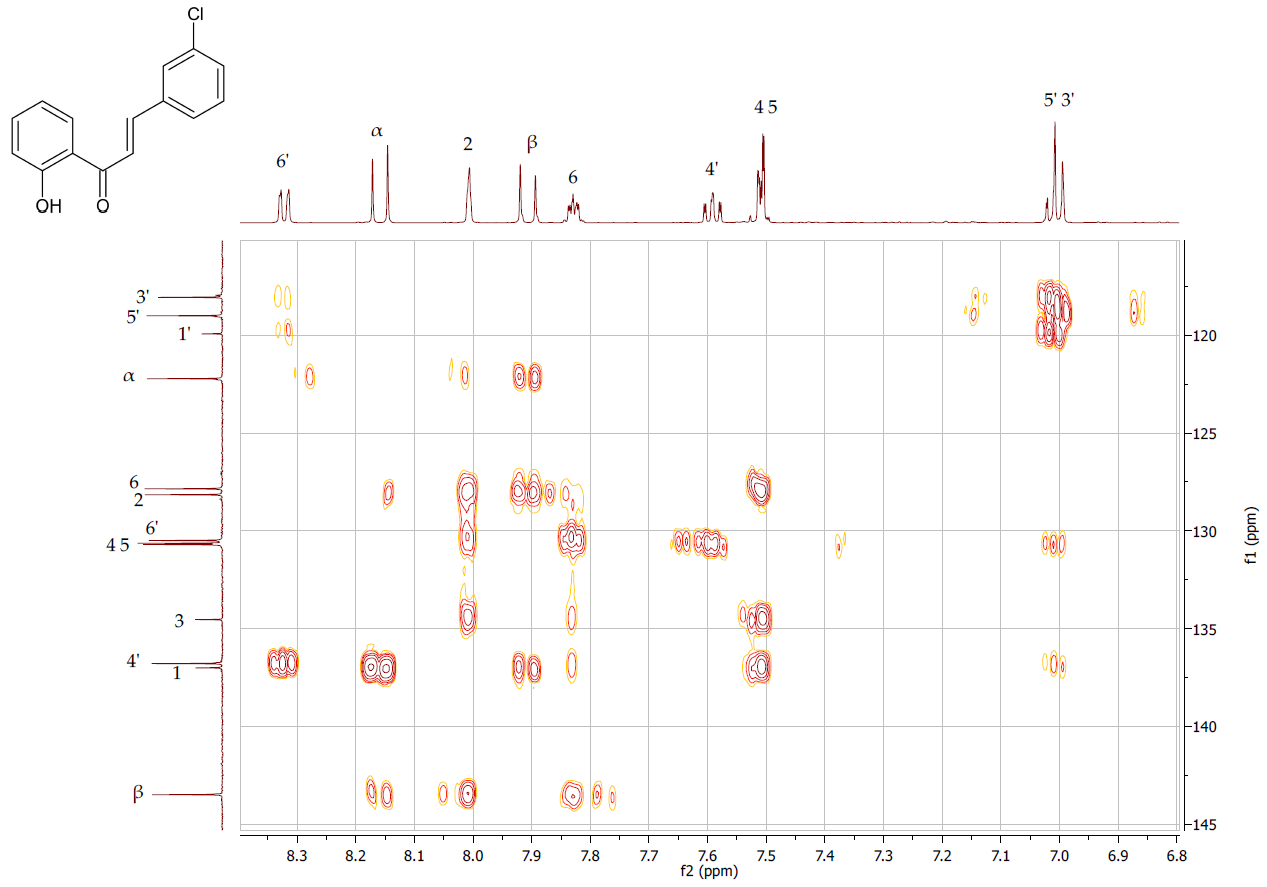


**Figure S30.** HMBC contour map – ^1^H x ^13^C expansion of 3-chloro-2’-hydroxychalcone (**5**)

**
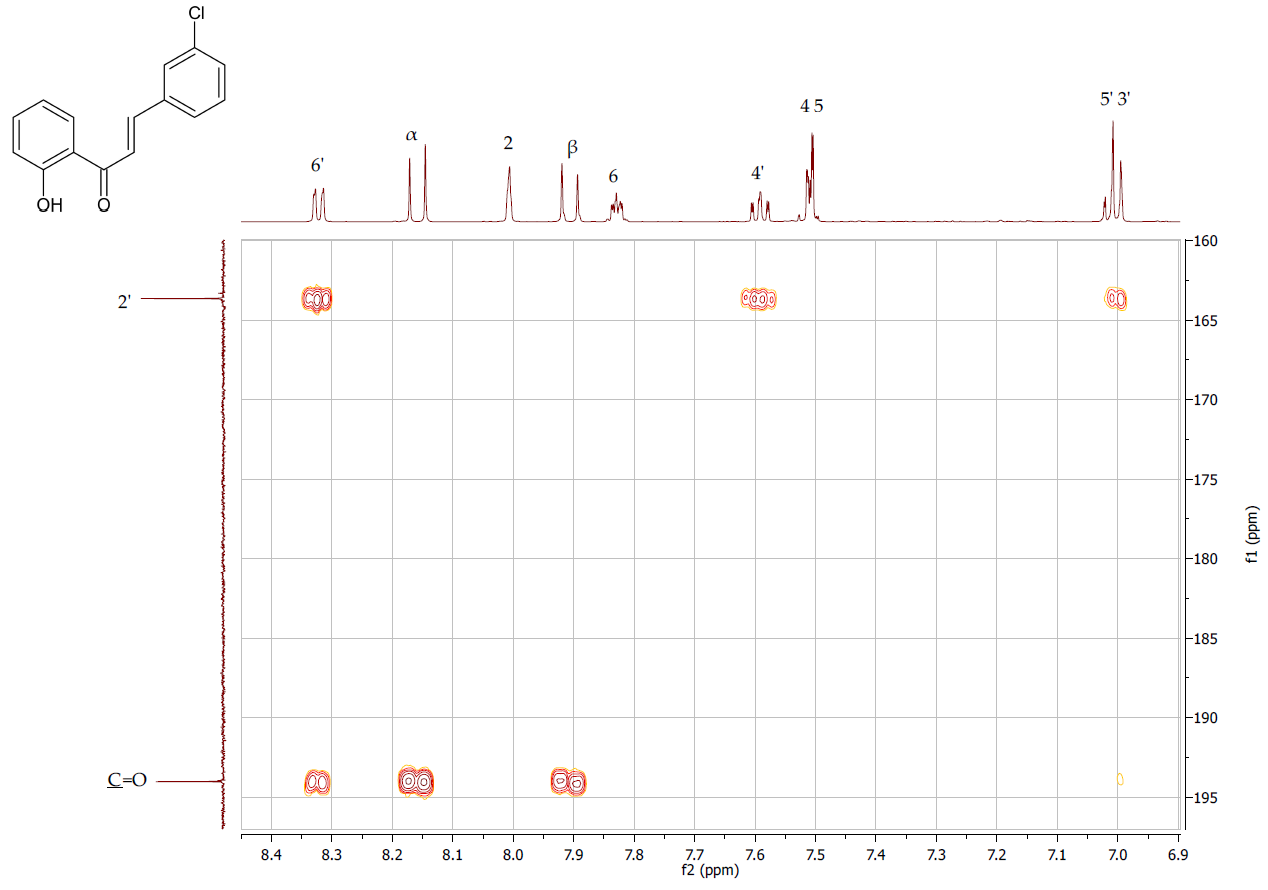
**

**Figure S31.** HMBC contour map – ^1^H x ^13^C expansion of 3-chloro-2’-hydroxychalcone (**5**)


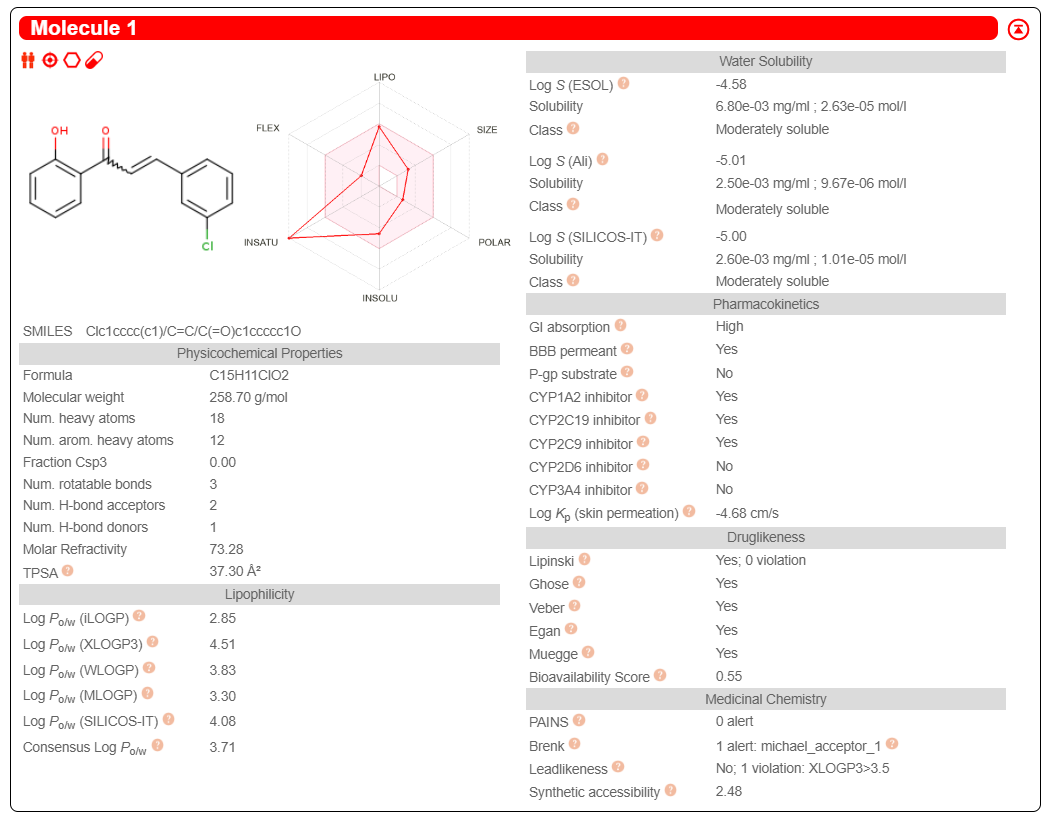


**Figure S32.** 3-Chloro-2’-hydroxychalcone (**5**) physicochemical and ADME parameters prediction using the SwissADME modelling


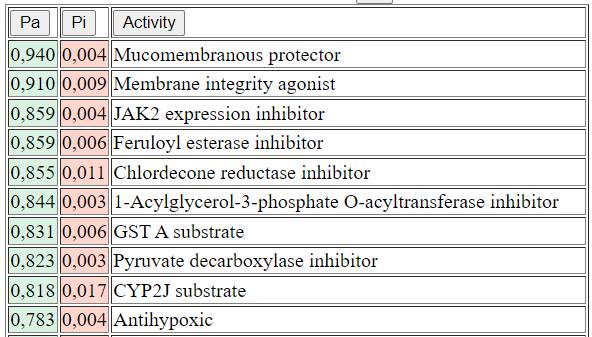


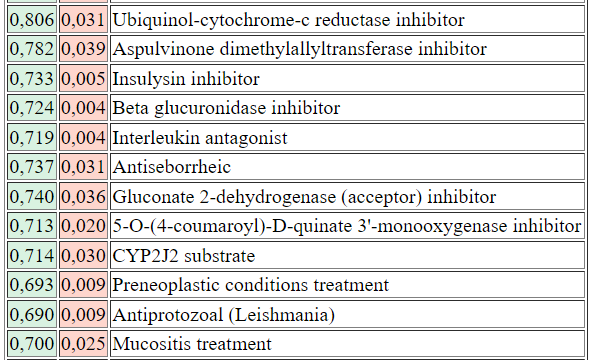


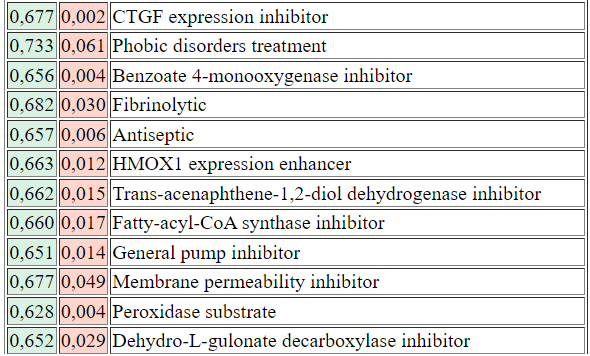


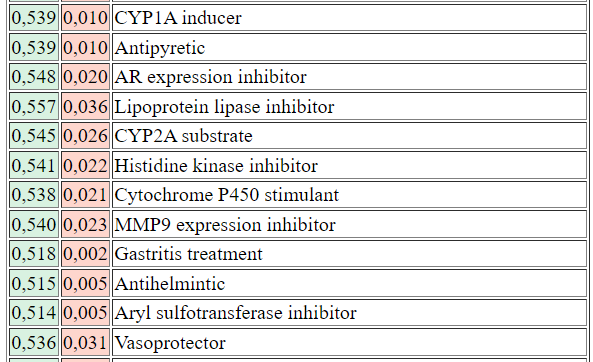


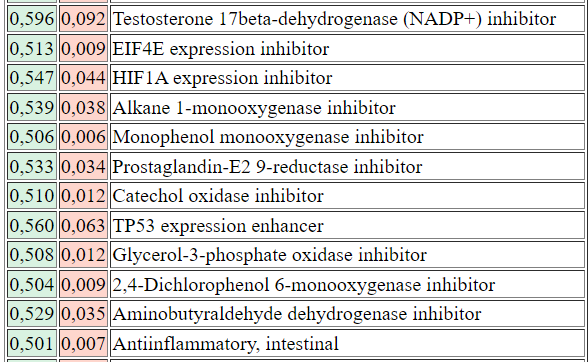


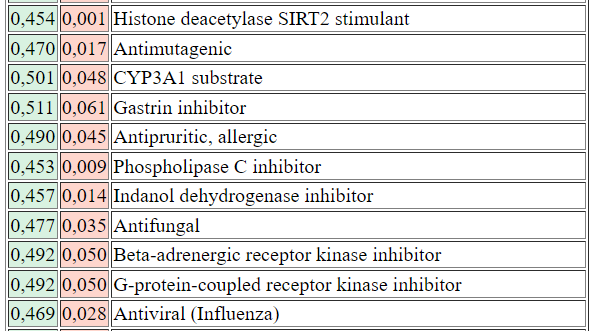


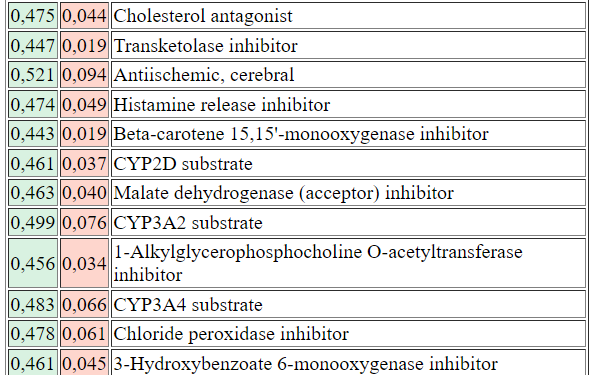


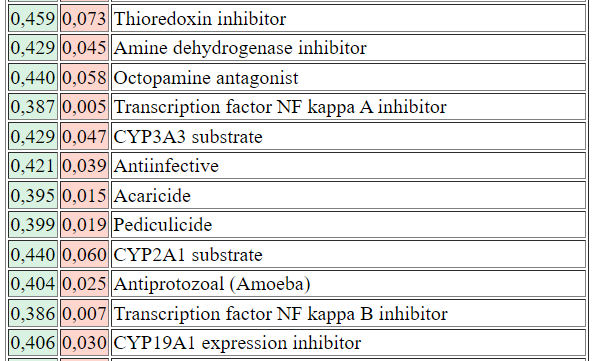


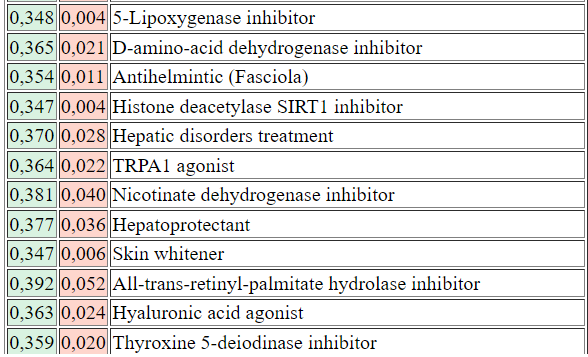


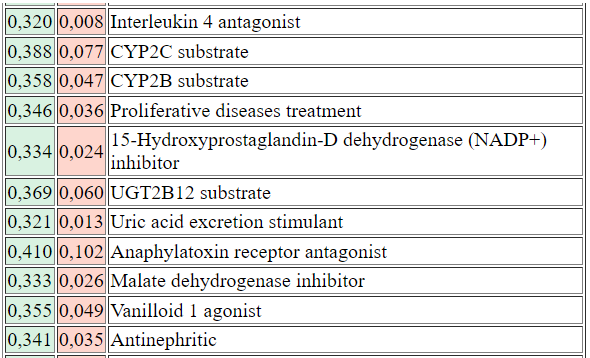


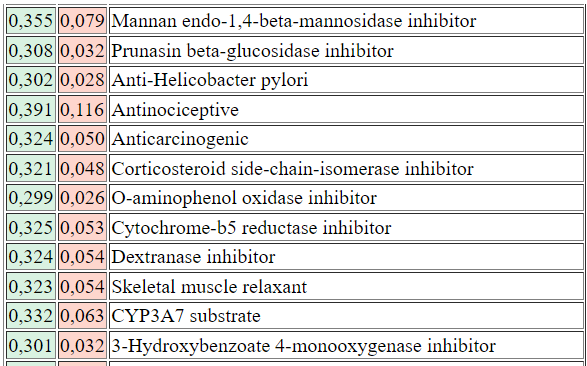


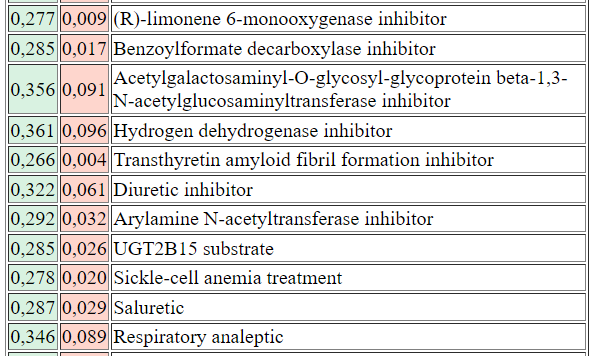


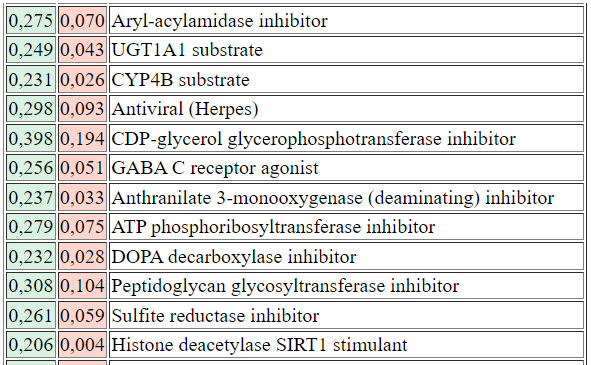


**Figure S33.** 3-Chloro-2’-hydroxychalcone (**5**) physicochemical biological activity prediction using the Way2Drug Pass online modelling


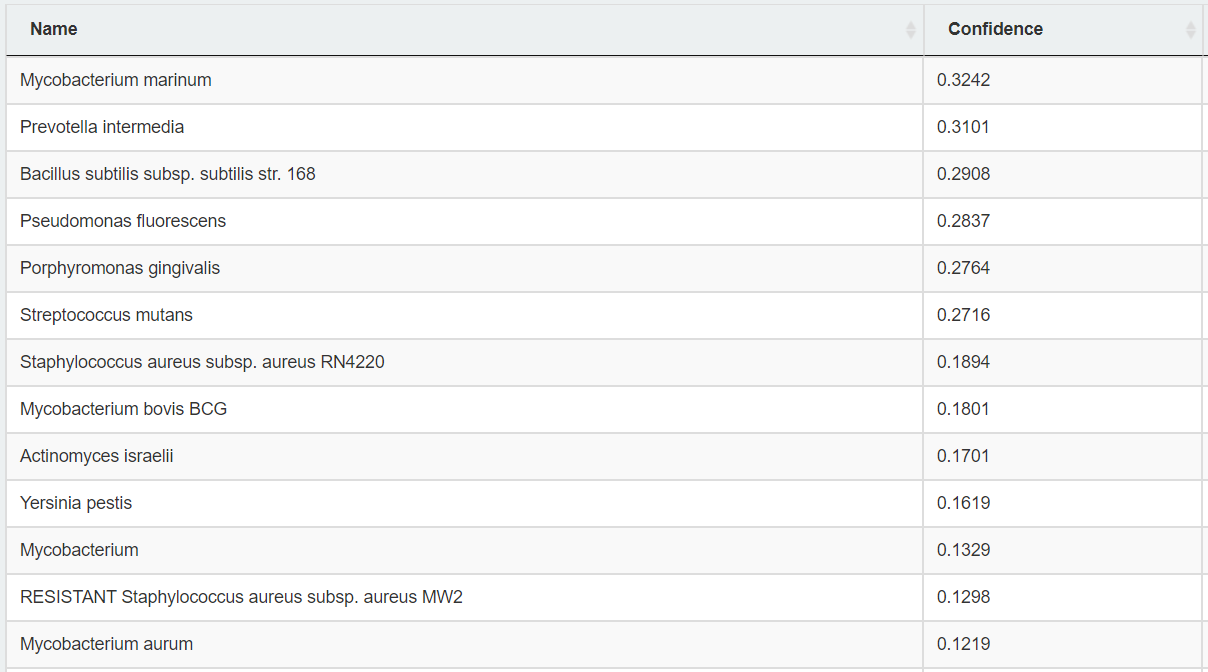


**Figure S34.** 3-Chloro-2’-hydroxychalcone (**5**) antibacterial activity prediction using the Way2Drug AntiBac-Pred modelling


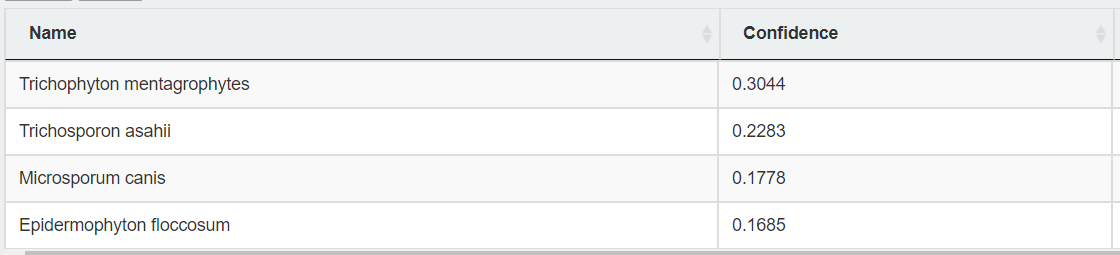


**Figure S35.** 3-Chloro-2’-hydroxychalcone (**5**) antifungal activity prediction using the Way2Drug AntiFun-Pred modelling


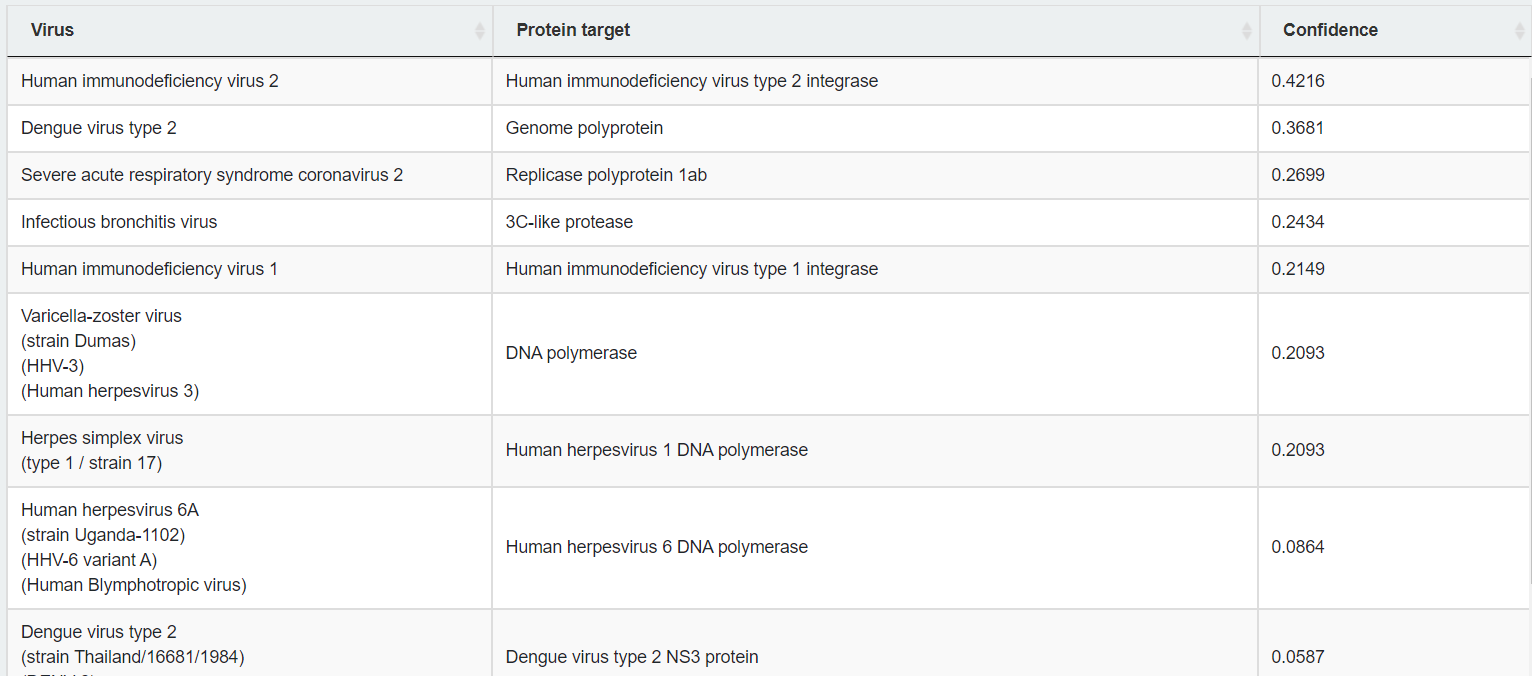


**Figure S36.** 3-Chloro-2’-hydroxychalcone (**5**) antiviral activity prediction using the Way2Drug AntiVir-Pred modelling


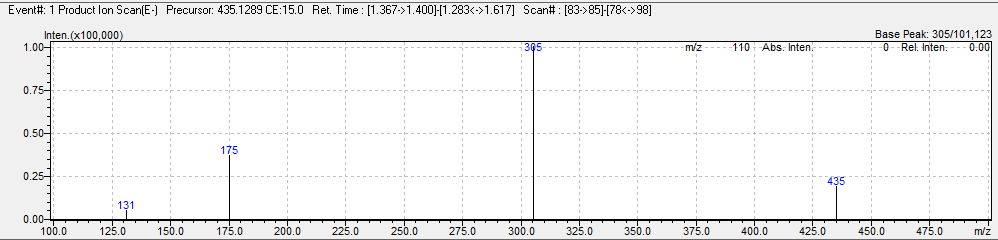


**Figure S37.** MS analysis of 2-chlorodihydrochalcone 2’-*O*-*β*-D-(4’’-*O*-methyl)-glucopyranoside (**3a**)


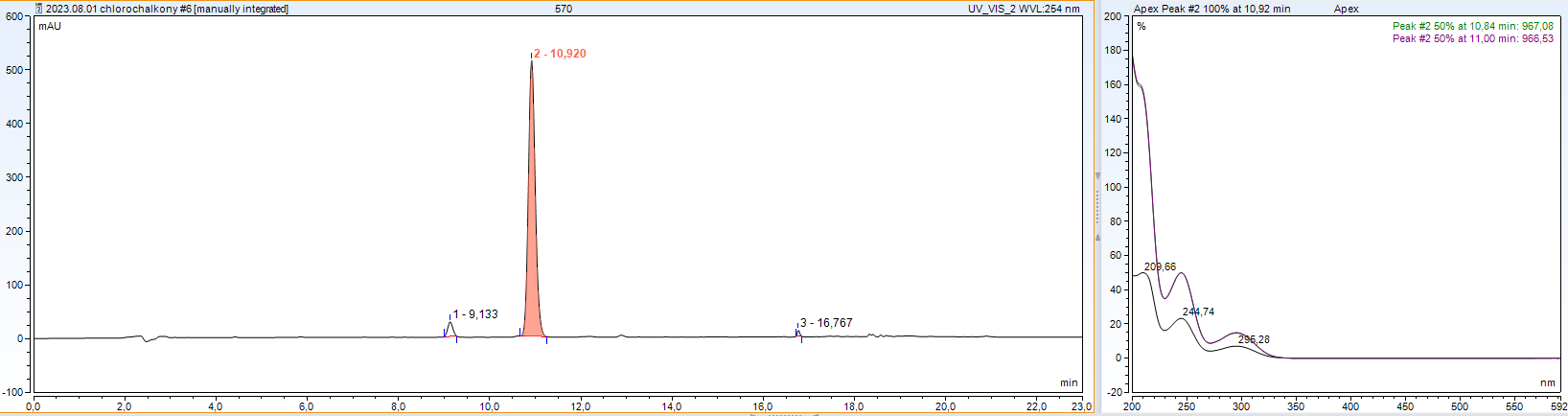


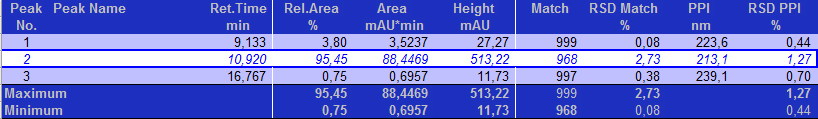


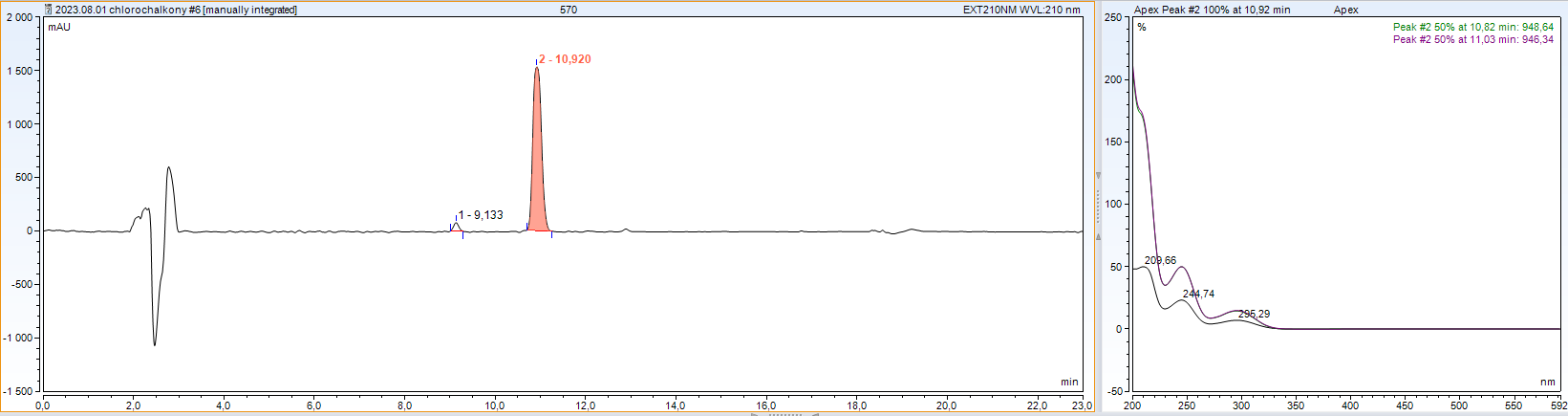


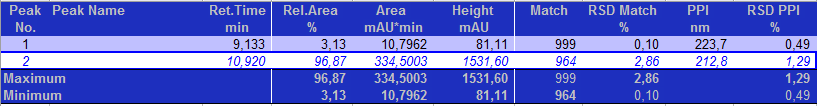


**Figure S38.** HPLC analysis of 2-chlorodihydrochalcone 2’-*O*-*β*-D-(4’’-*O*-methyl)-glucopyranoside (**3a**)


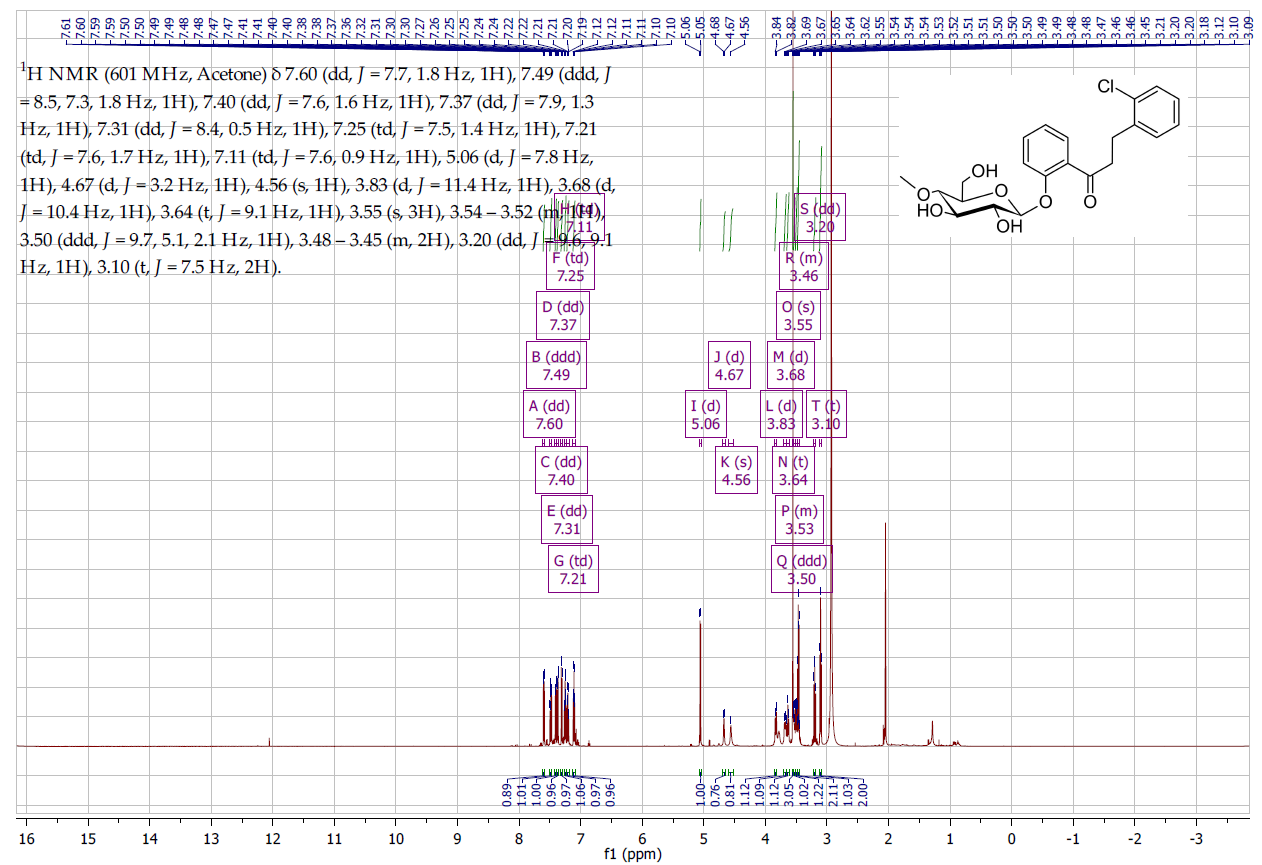


**Figure S39.** ^1^H NMR spectrum (*δ,* acetone-d6, 600 MHz) of 2-chlorodihydrochalcone 2’-*O*-*β*-D-(4’’-*O*-methyl)-glucopyranoside (**3a**)


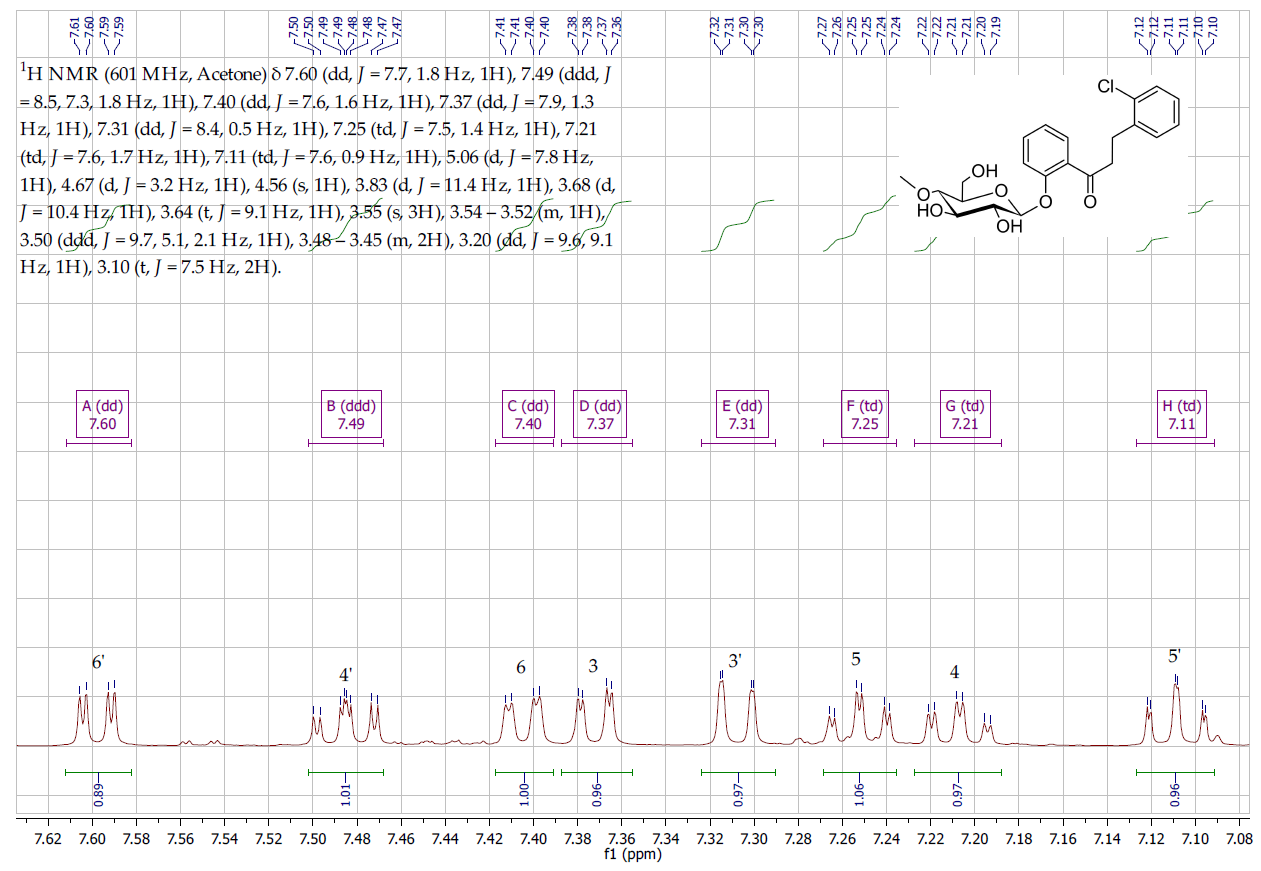


**Figure S40** ^1^H NMR spectrum expansion (*δ,* acetone-d6, 600 MHz) of 2-chlorodihydrochalcone 2’-*O*-*β*-D-(4’’-*O*-methyl)-glucopyranoside (**3a**)


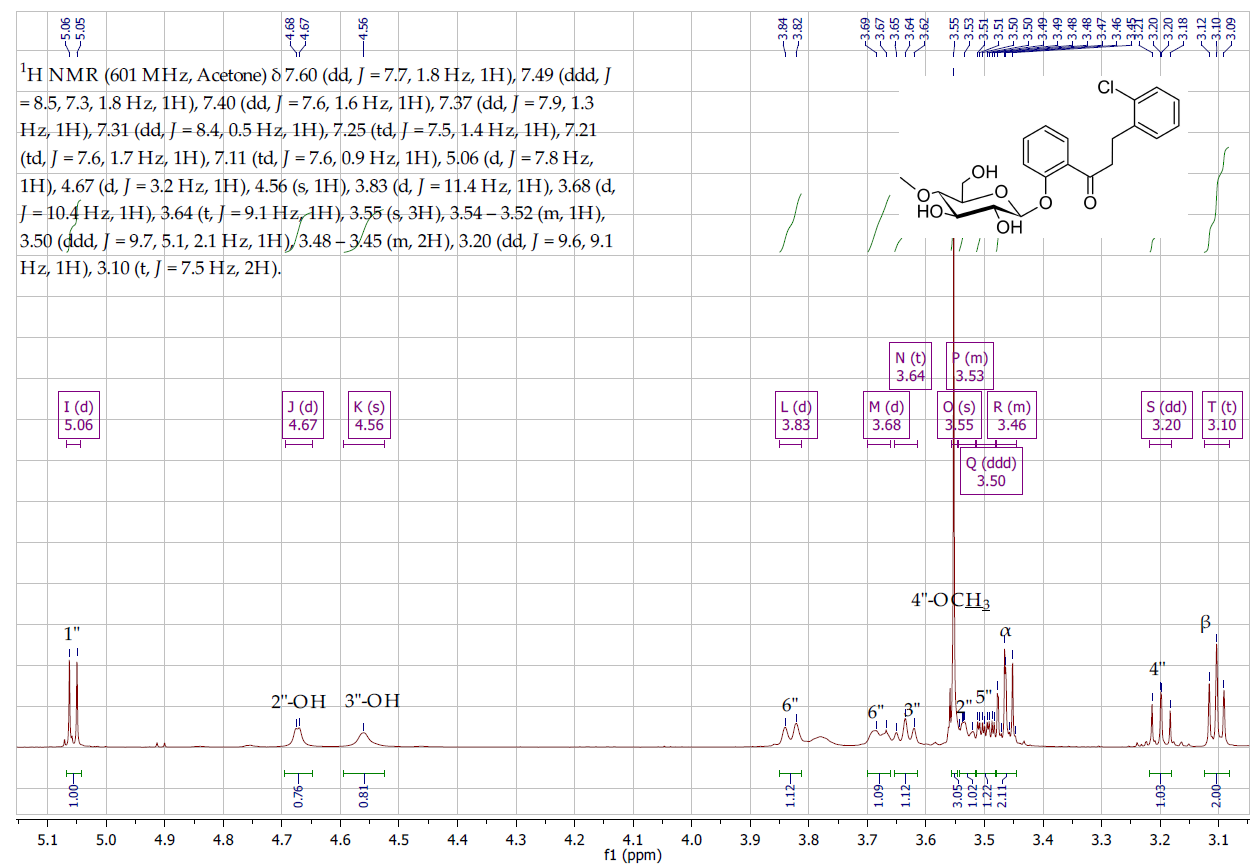


**Figure S41** ^1^H NMR spectrum expansion (*δ,* acetone-d6, 600 MHz) of 2-chlorodihydrochalcone 2’-*O*-*β*-D-(4’’-*O*-methyl)-glucopyranoside (**3a**)


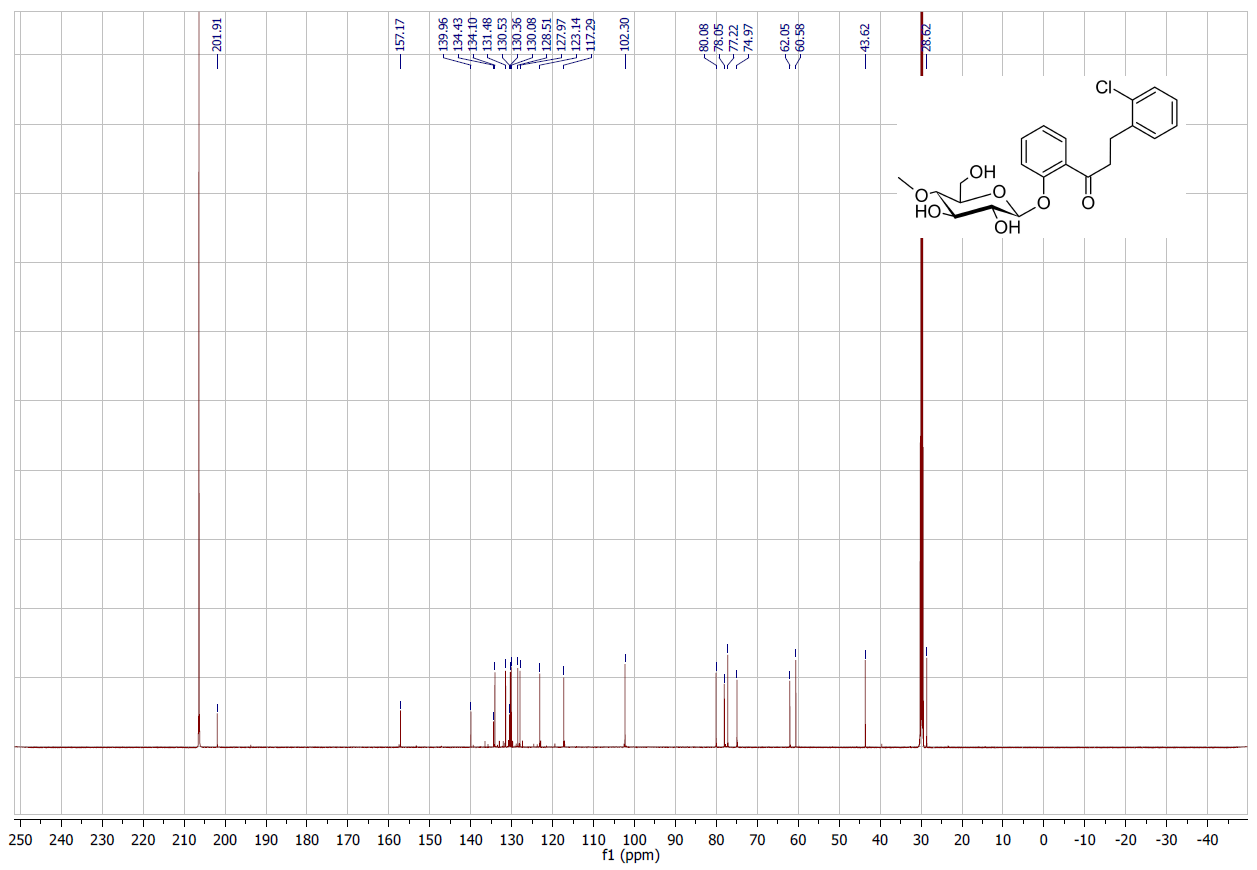


**Figure S42.** ^13^C NMR spectrum (*δ,* acetone-d6, 151 MHz) of 2-chlorodihydrochalcone 2’-*O*-*β*-D-(4’’-*O*-methyl)-glucopyranoside (**3a**)


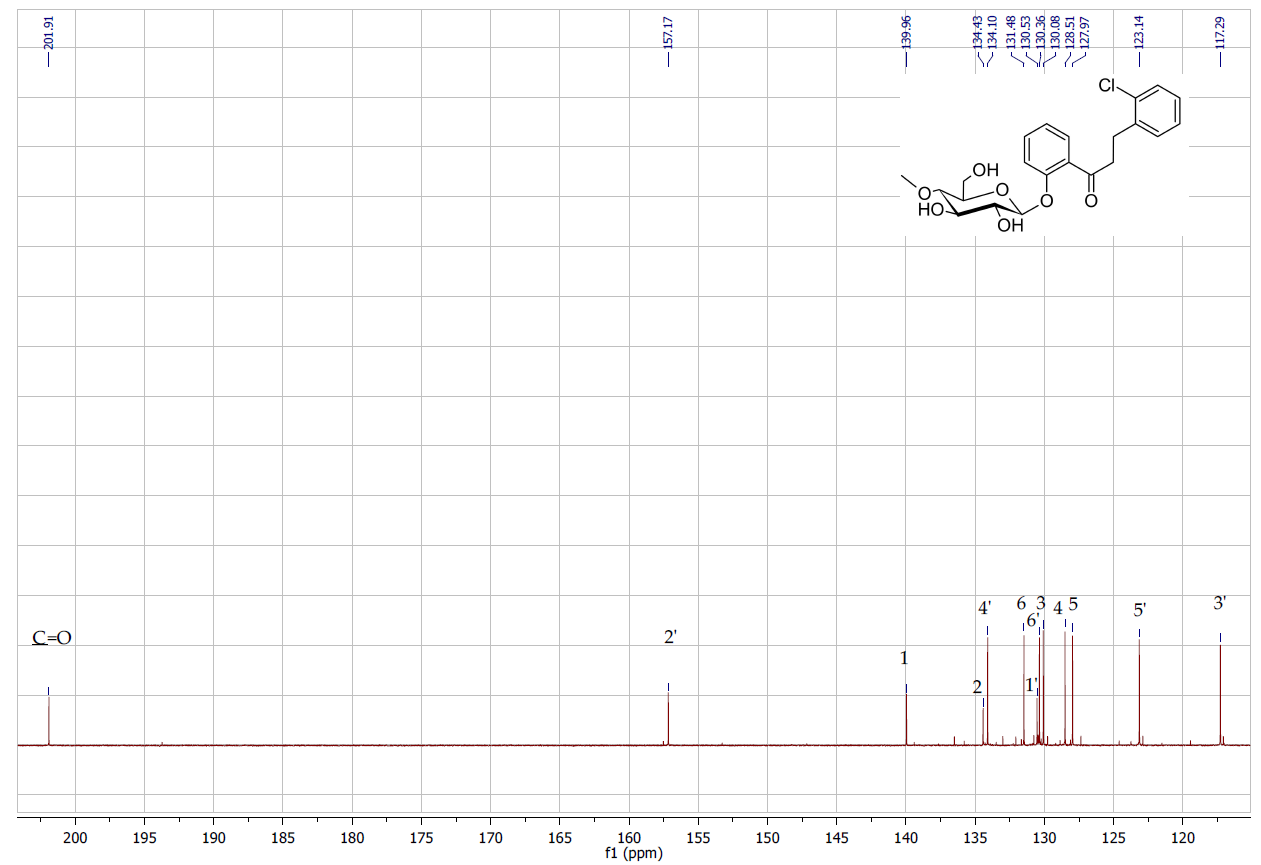


**Figure S43.** ^13^C NMR spectrum expansion (*δ,* acetone-d6, 151 MHz) of 2-chlorodihydrochalcone 2’-*O*-*β*-D-(4’’-*O*-methyl)-glucopyranoside (**3a**)


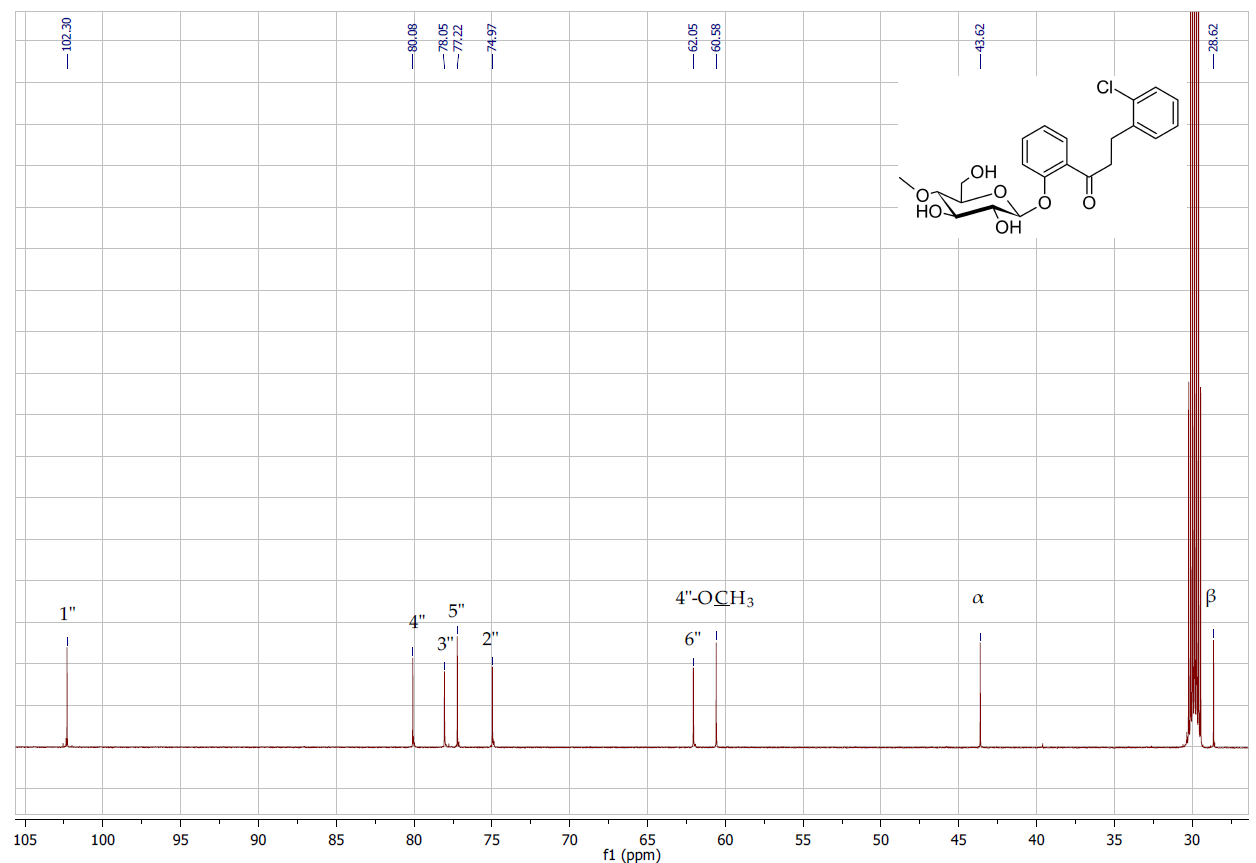


**Figure S44.** ^13^C NMR spectrum expansion (*δ,* acetone-d6, 151 MHz) of 2-chlorodihydrochalcone 2’-*O*-*β*-D-(4’’-*O*-methyl)-glucopyranoside (**3a**)


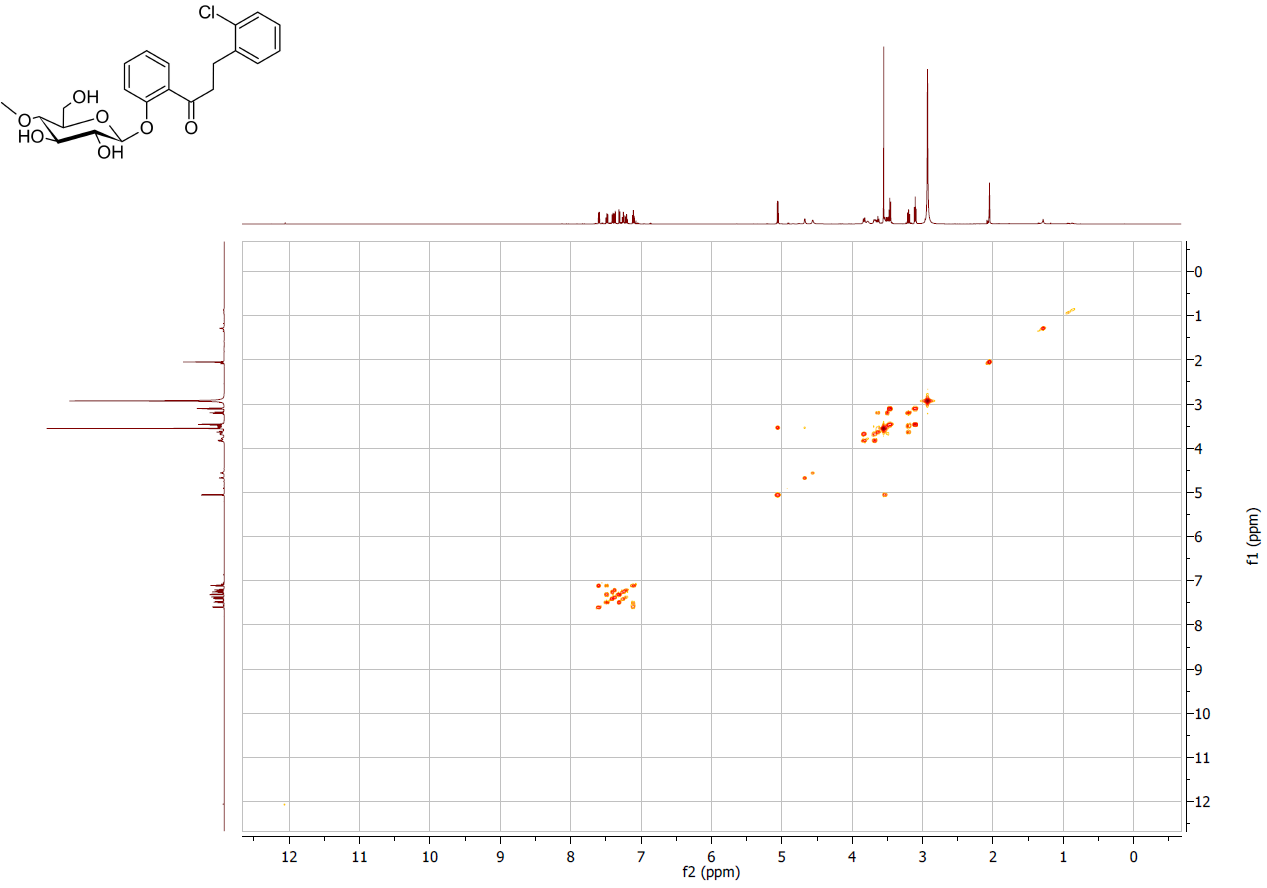


**Figure S45.** COSY contour map – ^1^H x ^1^H of 2-chlorodihydrochalcone 2’-*O*-*β*-D-(4’’-*O*-methyl)-glucopyranoside (**3a**)


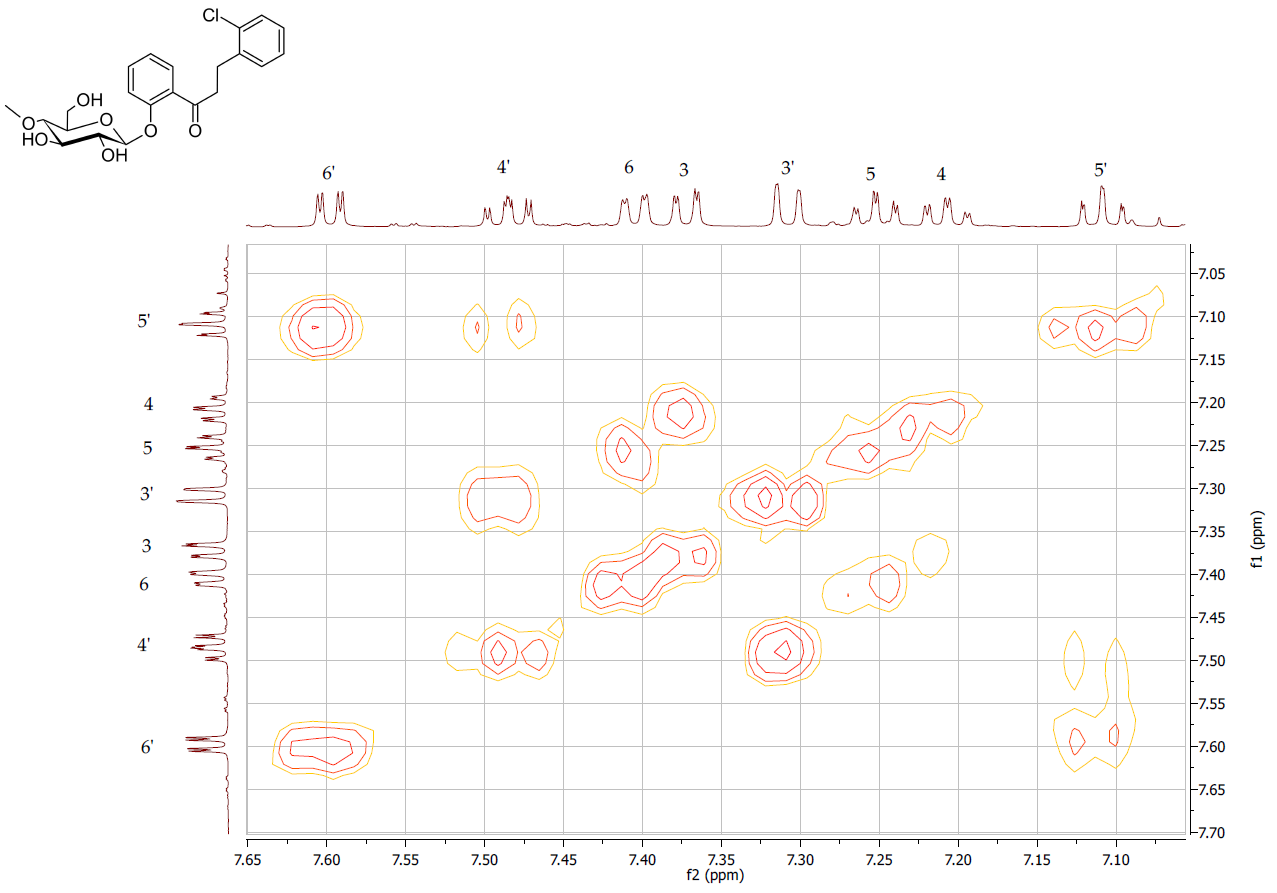


**Figure S46.** COSY contour map – ^1^H x ^1^H expansion of 2-chlorodihydrochalcone 2’-*O*-*β*-D-(4’’-*O*-methyl)-glucopyranoside (**3a**)


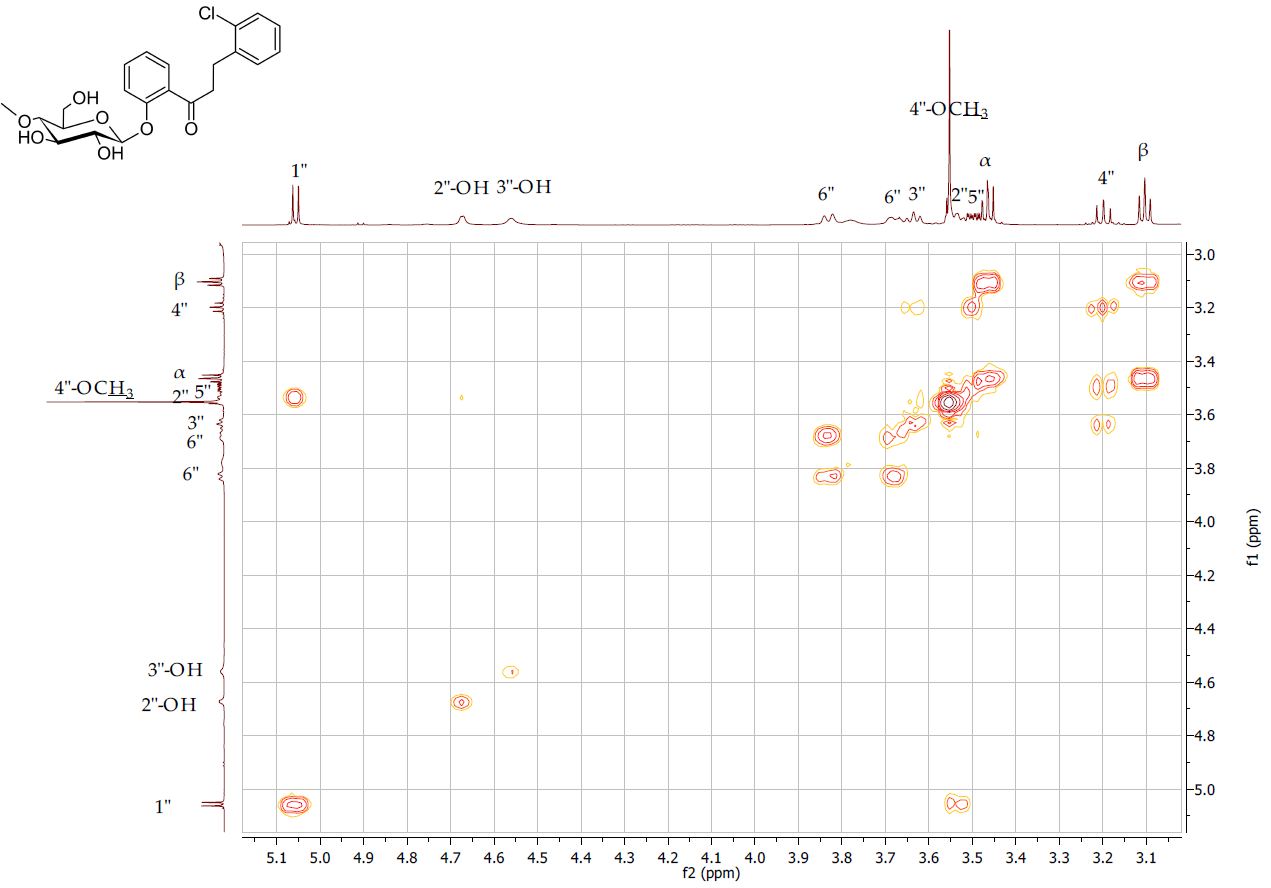


**Figure S47.** COSY contour map – ^1^H x ^1^H expansion of 2-chlorodihydrochalcone 2’-*O*-*β*-D-(4’’-*O*-methyl)-glucopyranoside (**3a**)


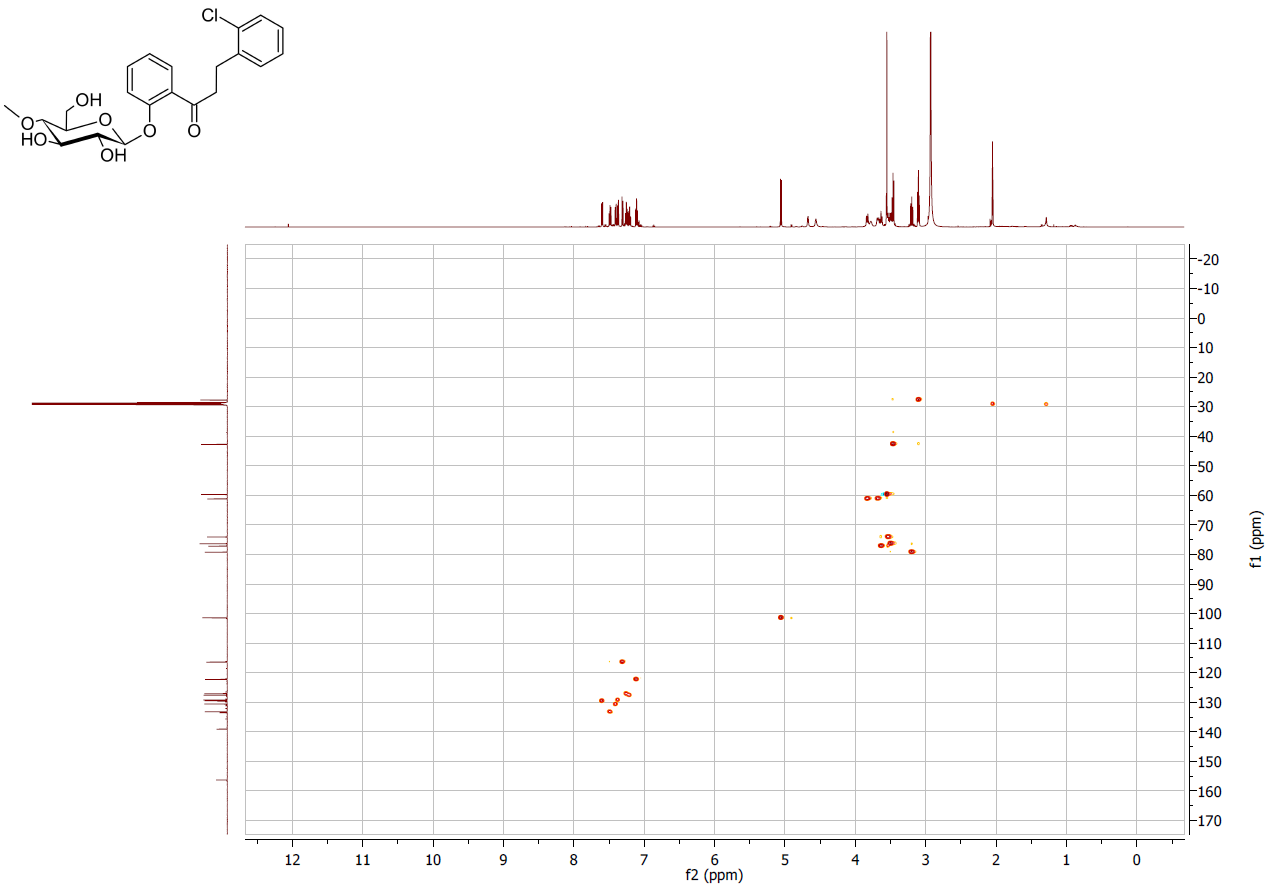


**Figure S48.** HMQC contour map – ^1^H x ^13^C of 2-chlorodihydrochalcone 2’-*O*-*β*-D-(4’’-*O*-methyl)-glucopyranoside (**3a**)


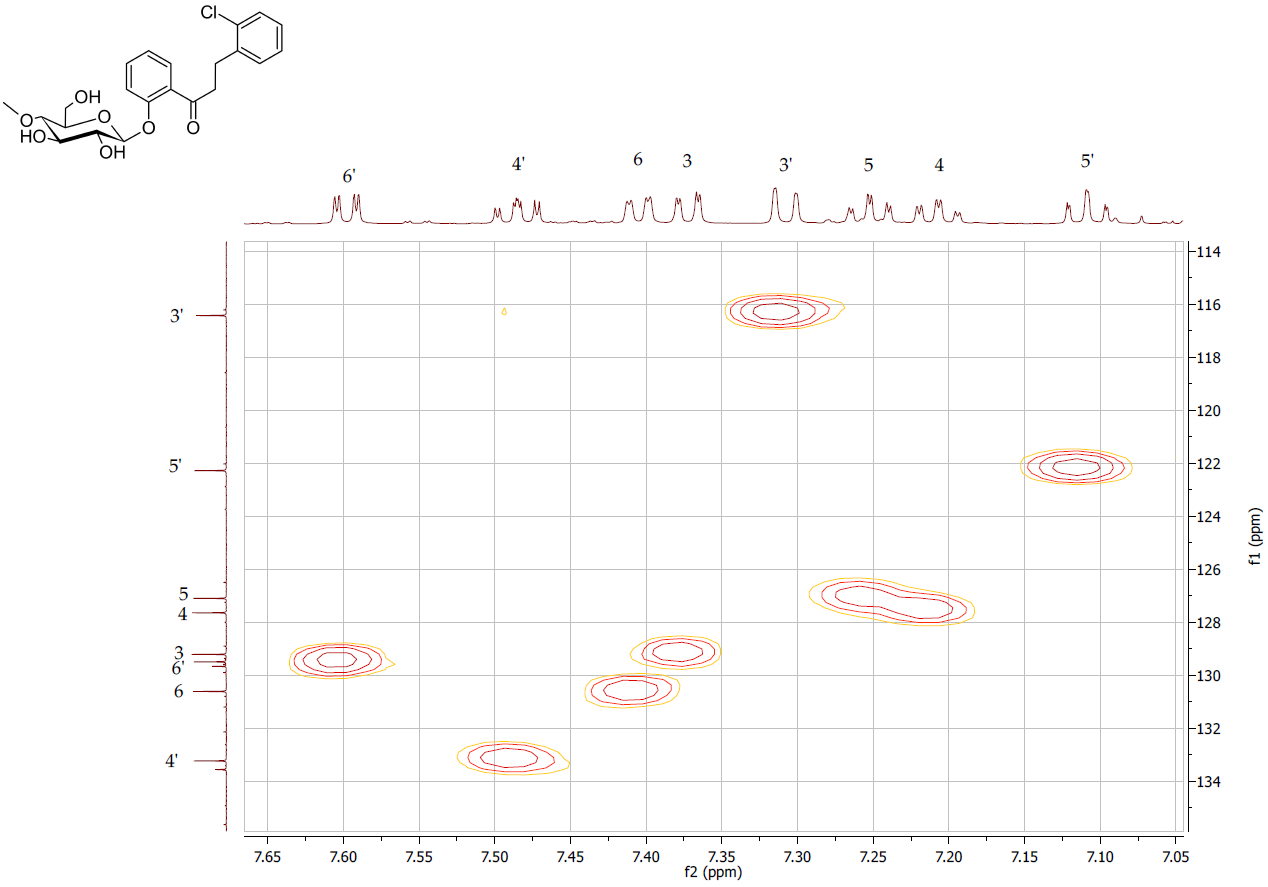


**Figure S49.** HMQC contour map – ^1^H x ^13^C expansion of 2-chlorodihydrochalcone 2’-*O*-*β*-D-(4’’-*O*-methyl)-glucopyranoside (**3a**)


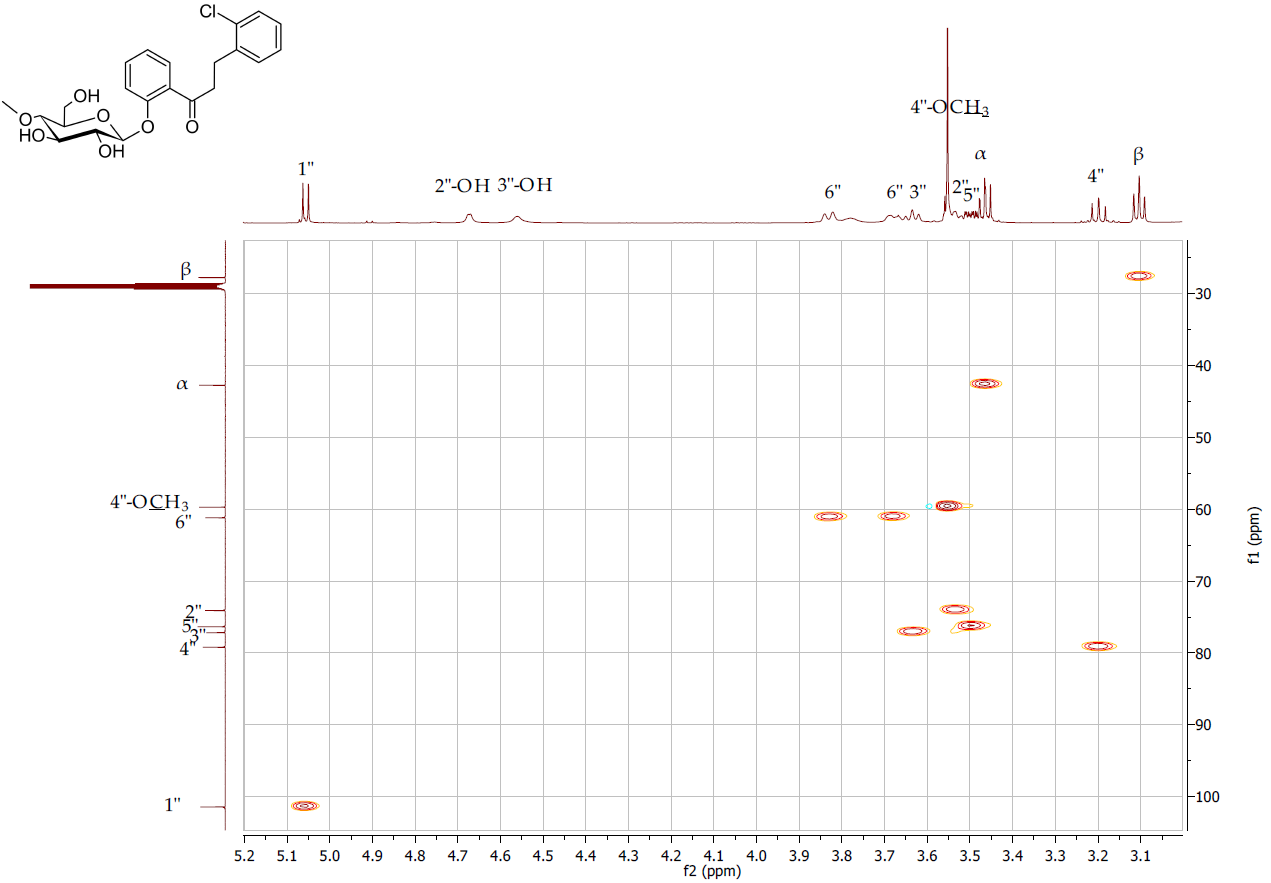


**Figure S50.** HMQC contour map – ^1^H x ^13^C expansion of 2-chlorodihydrochalcone 2’-*O*-*β*-D-(4’’-*O*-methyl)-glucopyranoside (**3a**)


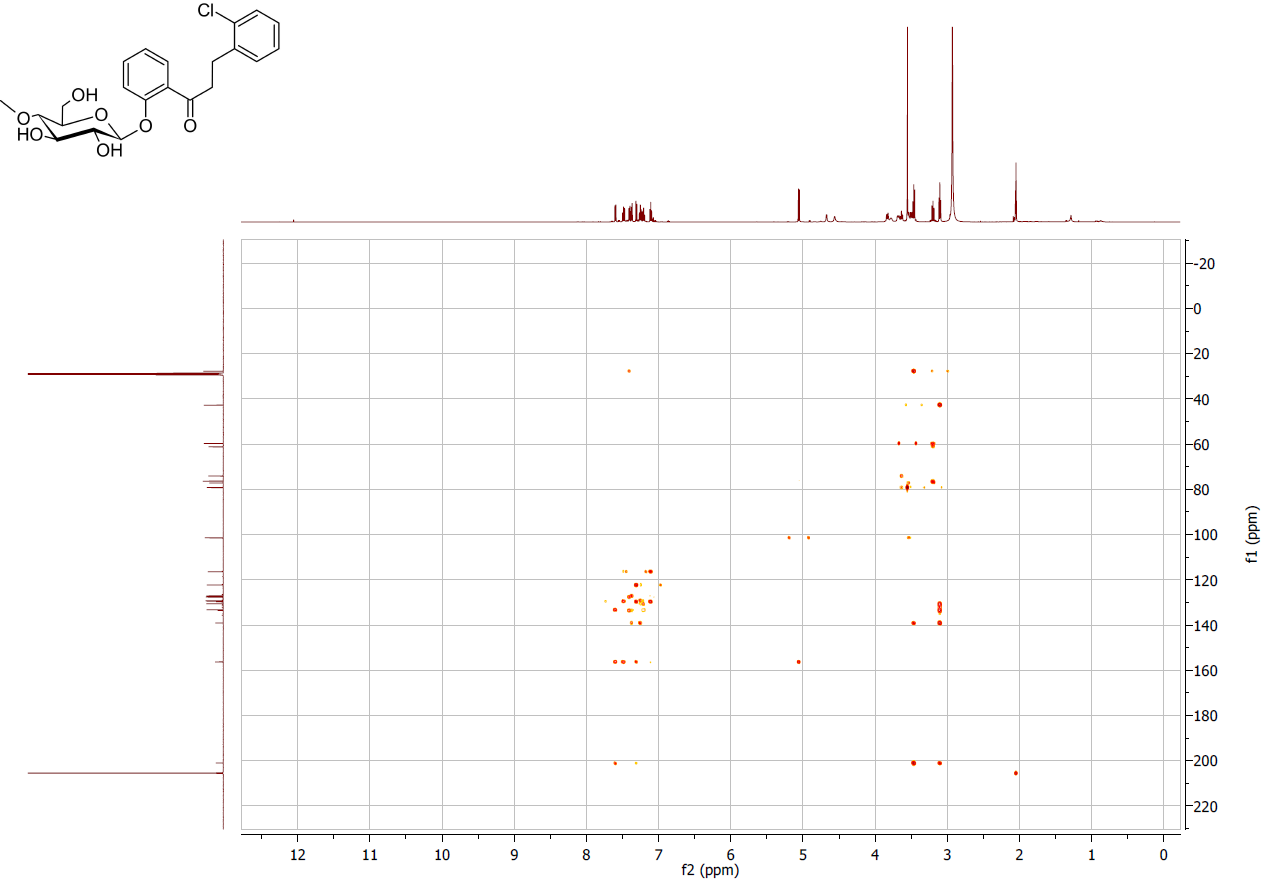


**Figure S51.** HMBC contour map – ^1^H x ^13^C of 2-chlorodihydrochalcone 2’-*O*-*β*-D-(4’’-*O*-methyl)-glucopyranoside (**3a**)


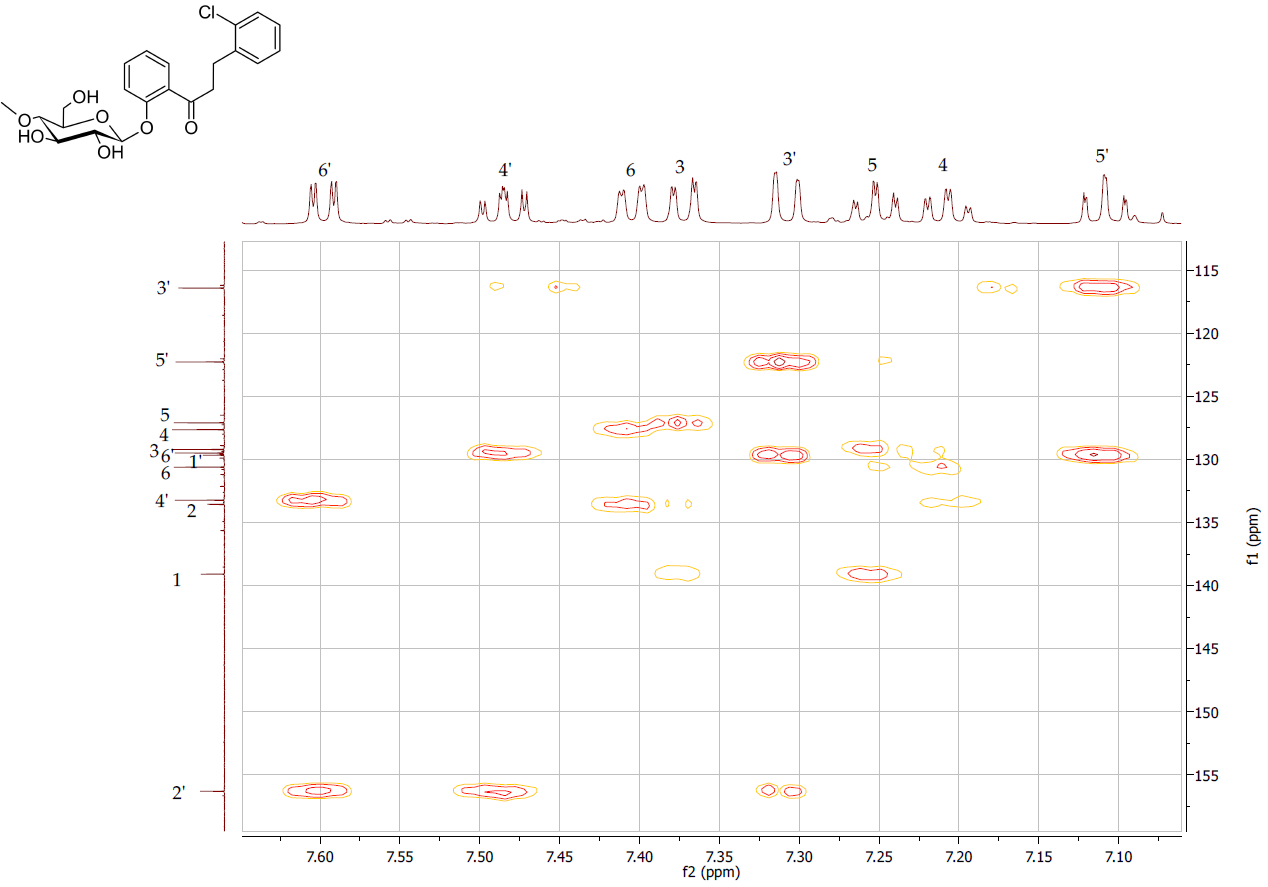


**Figure S52.** HMBC contour map – ^1^H x ^13^C expansion of 2-chlorodihydrochalcone 2’-*O*-*β*-D-(4’’-*O*-methyl)-glucopyranoside (**3a**)


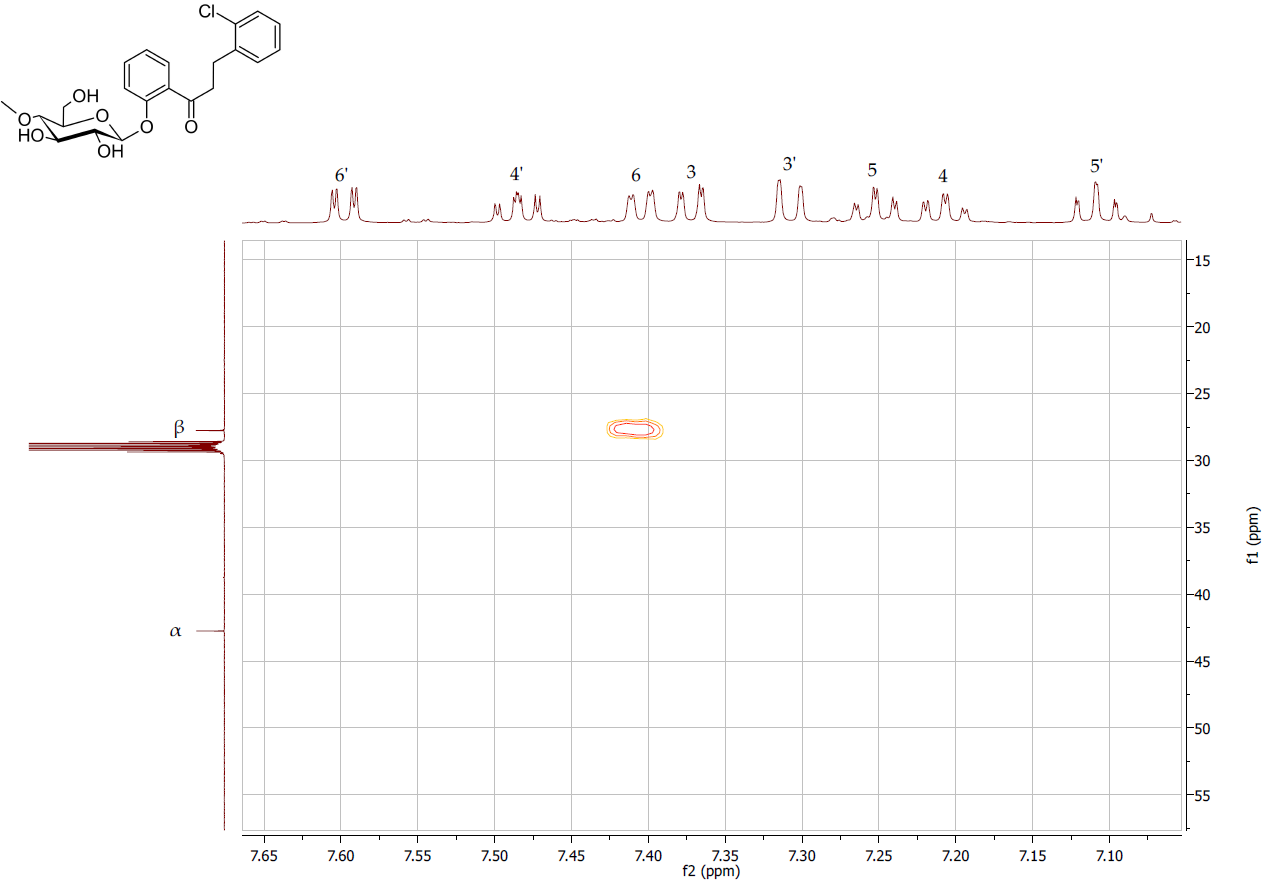


**Figure S53.** HMBC contour map – ^1^H x ^13^C expansion of 2-chlorodihydrochalcone 2’-*O*-*β*-D-(4’’-*O*-methyl)-glucopyranoside (**3a**)


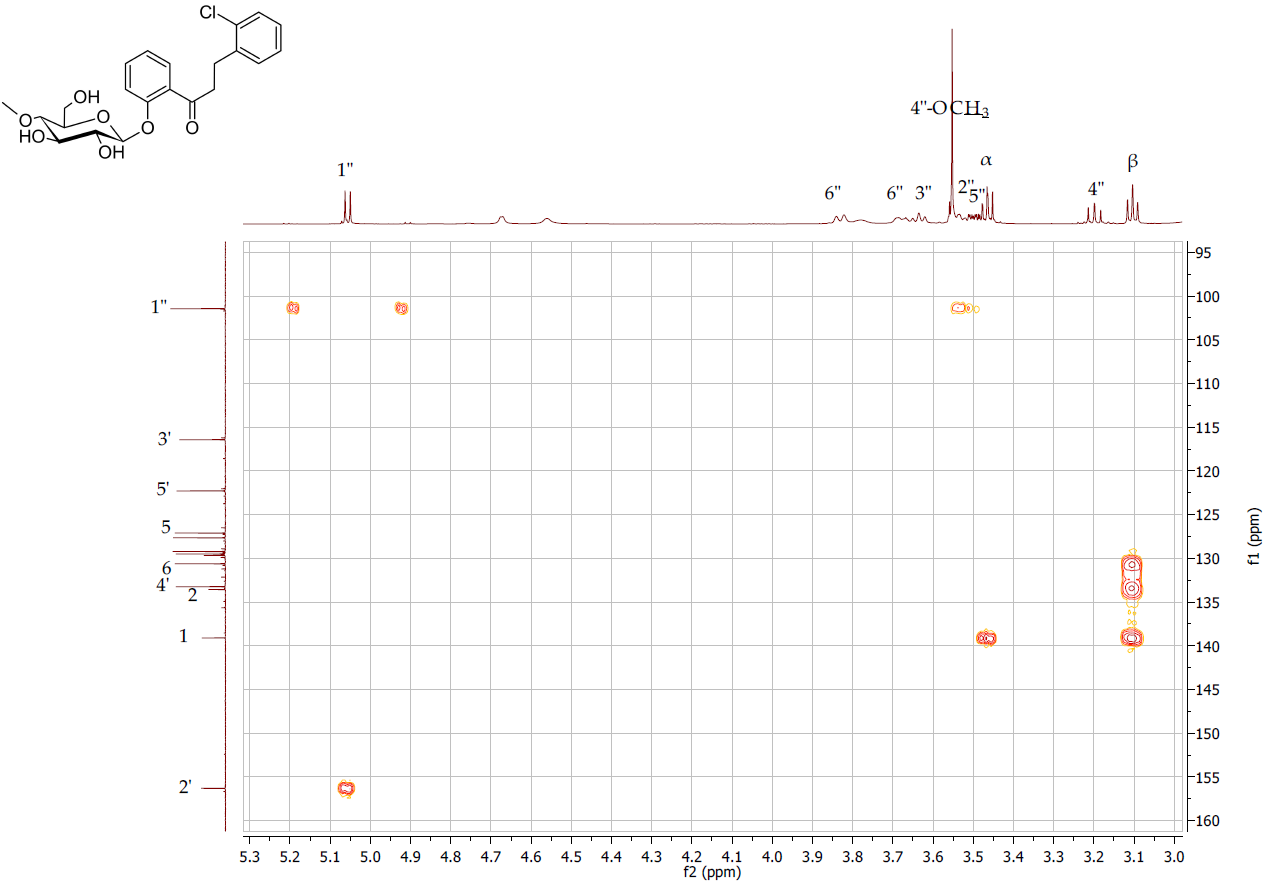


**Figure S54.** HMBC contour map – ^1^H x ^13^C expansion of 2-chlorodihydrochalcone 2’-*O*-*β*-D-(4’’-*O*-methyl)-glucopyranoside (**3a**)


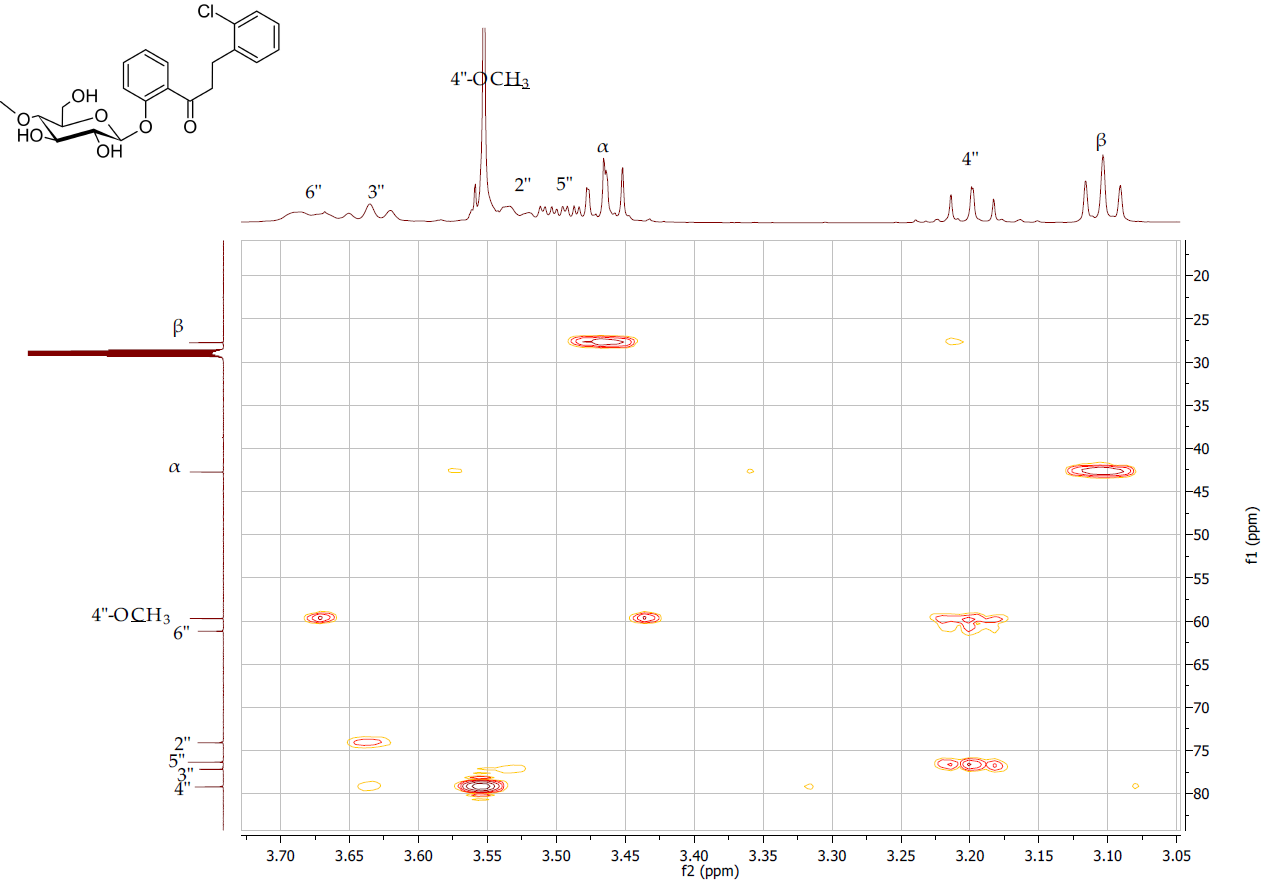


**Figure S55.** HMBC contour map – ^1^H x ^13^C expansion of 2-chlorodihydrochalcone 2’-*O*-*β*-D-(4’’-*O*-methyl)-glucopyranoside (**3a**)


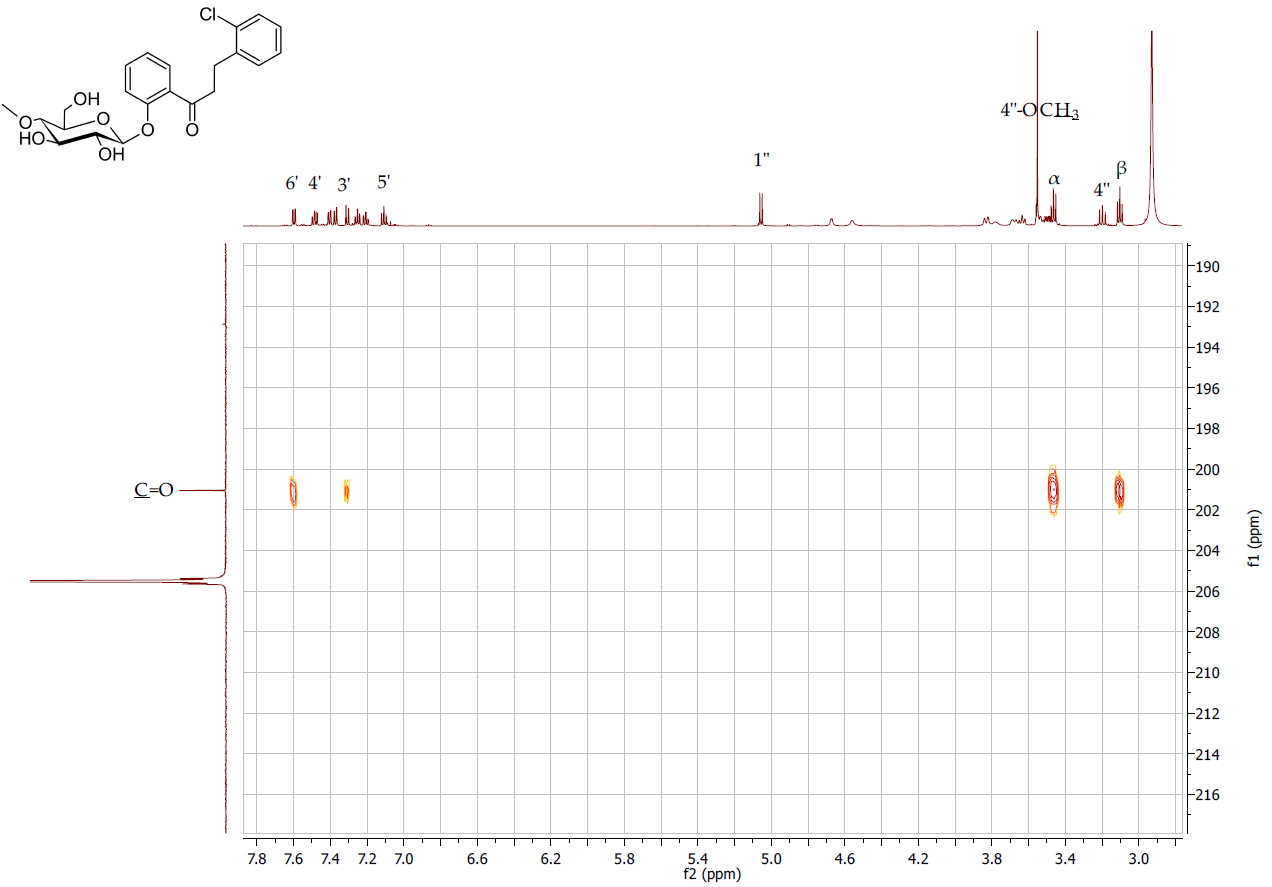


**Figure S56.** HMBC contour map – ^1^H x ^13^C expansion of 2-chlorodihydrochalcone 2’-*O*-*β*-D-(4’’-*O*-methyl)-glucopyranoside (**3a**)


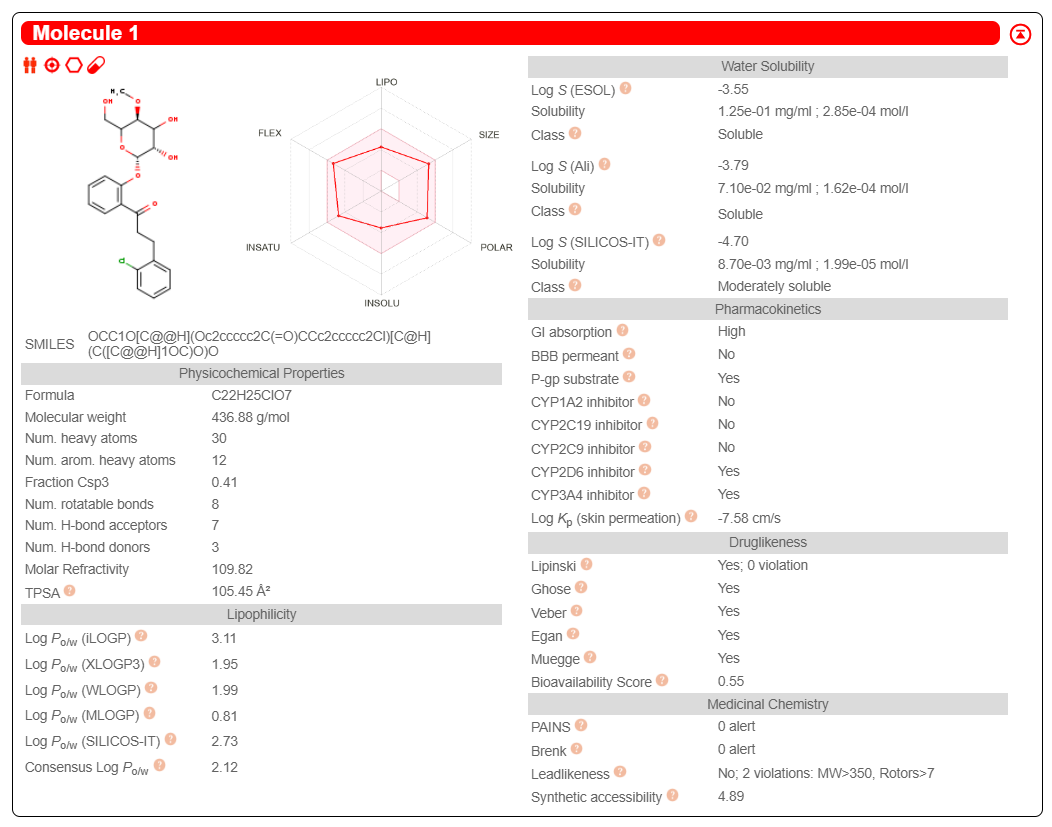


**Figure S57.** 2-Chlorodihydrochalcone 2’-*O*-*β*-D-(4’’-*O*-methyl)-glucopyranoside (**3a**) physicochemical and ADME parameters prediction using the SwissADME modelling


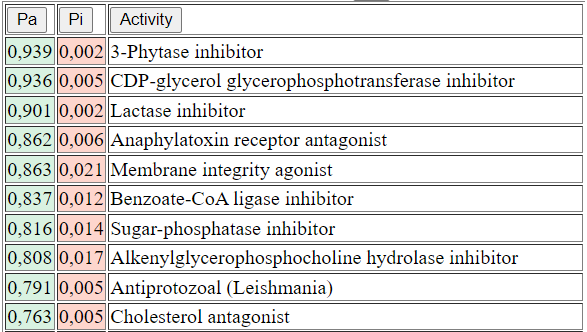


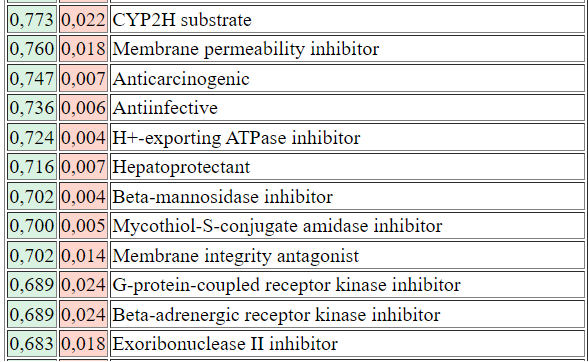


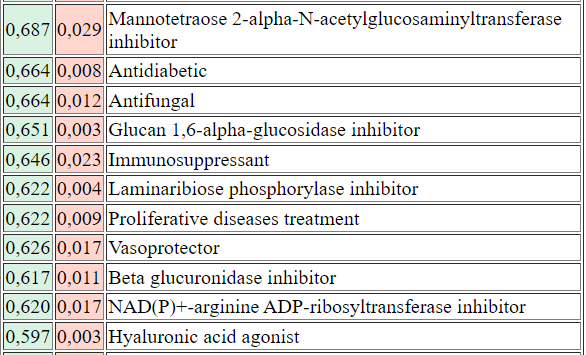


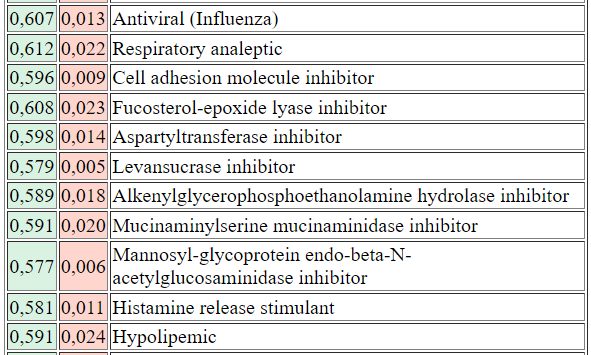


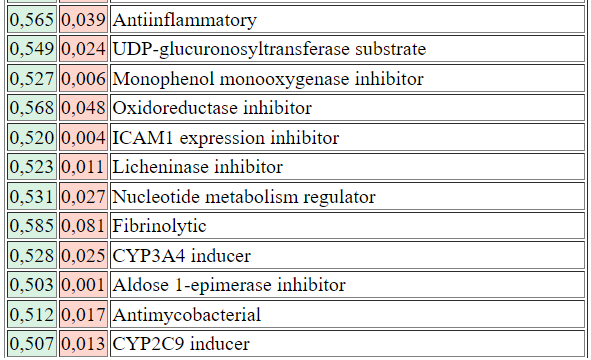


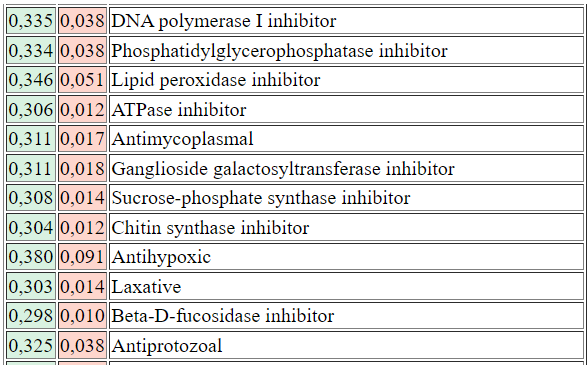


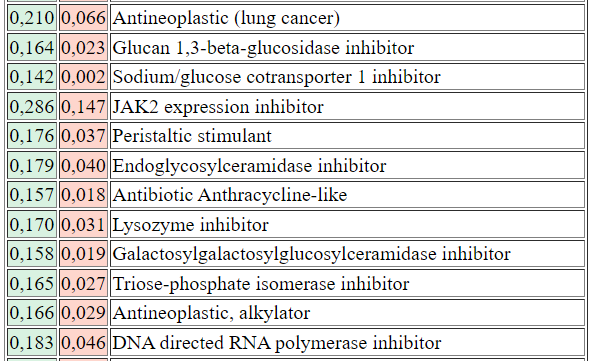


**Figure S58.** 2-Chlorodihydrochalcone 2’-*O*-*β*-D-(4’’-*O*-methyl)-glucopyranoside (**3a**) physicochemical biological activity prediction using the Way2Drug Pass online modelling


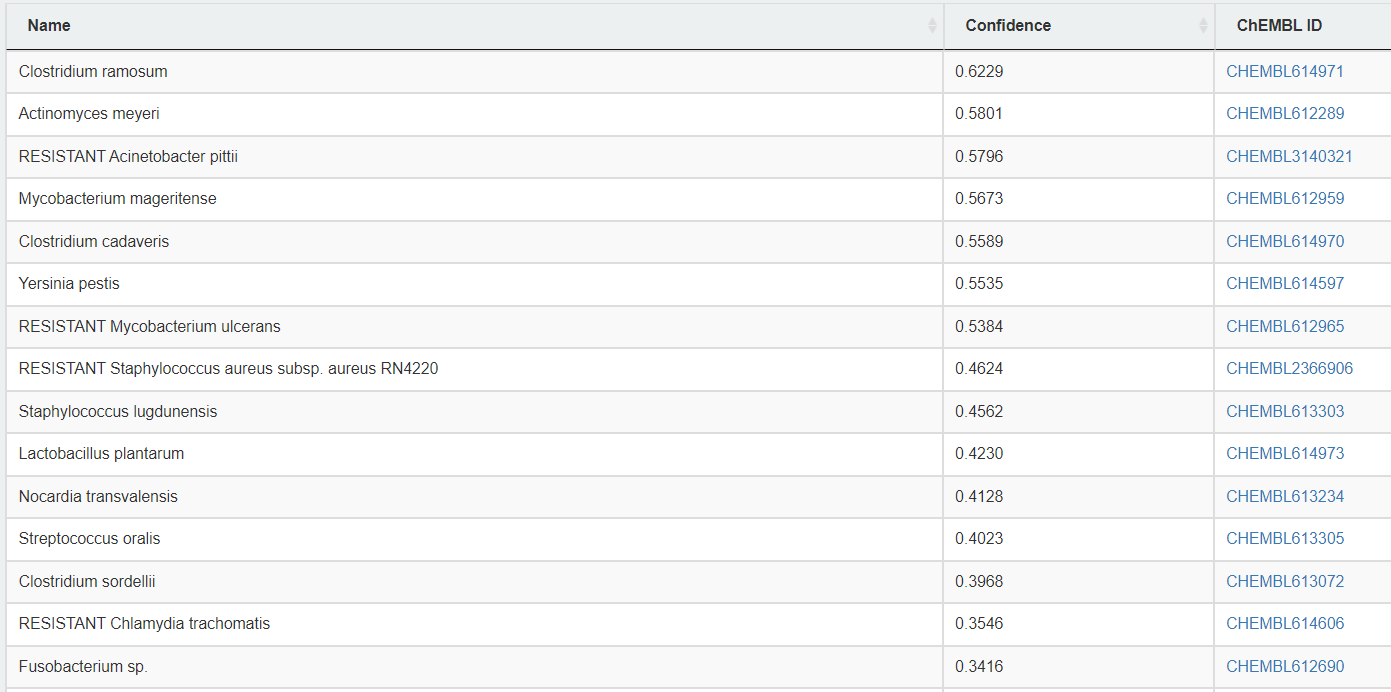


**Figure S59.** 2-Chlorodihydrochalcone 2’-*O*-*β*-D-(4’’-*O*-methyl)-glucopyranoside (**3a**) antibacterial activity prediction using the Way2Drug AntiBac-Pred modelling


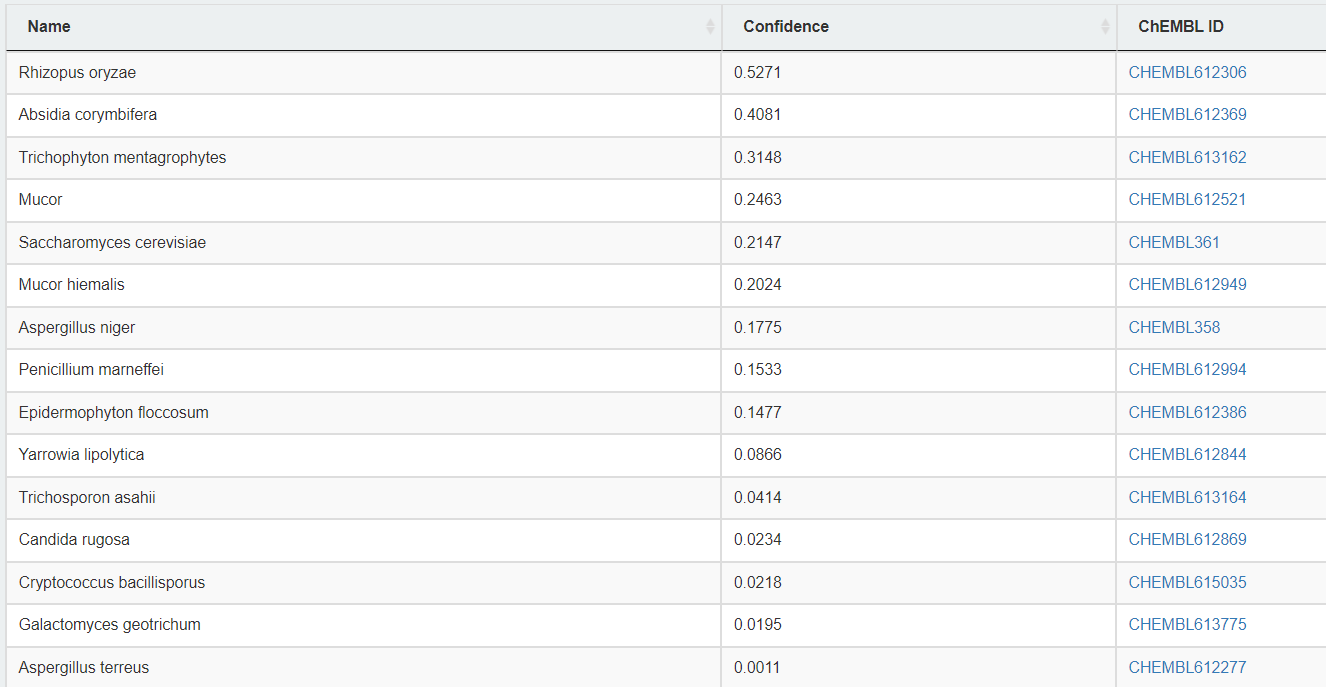


**Figure S60.** 2-Chlorodihydrochalcone 2’-*O*-*β*-D-(4’’-*O*-methyl)-glucopyranoside (**3a**) antifungal activity prediction using the Way2Drug AntiFun-Pred modelling


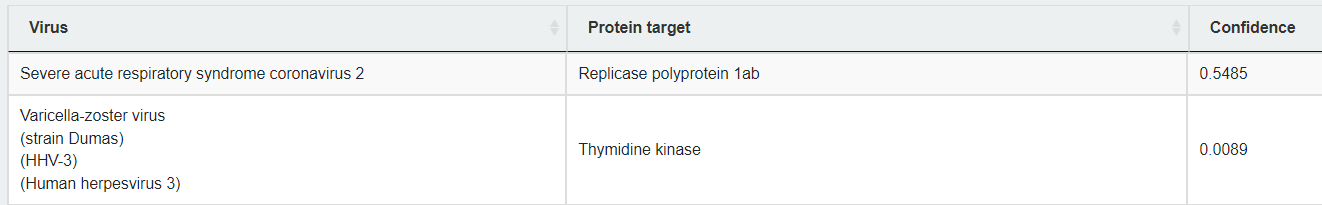


**Figure S61.** 2-Chlorodihydrochalcone 2’-*O*-*β*-D-(4’’-*O*-methyl)-glucopyranoside (**3a**) antiviral activity prediction using the Way2Drug AntiVir-Pred modelling


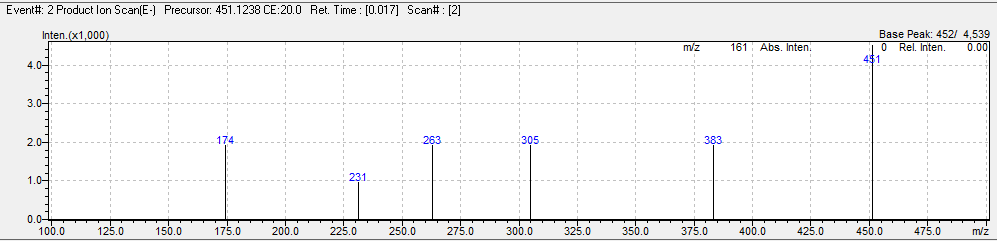


**Figure S62.** MS analysis of 2-chloro-2’-hydroxydihydrochalcone 5’-*O*-*β*-D-(4’’-*O*-methyl)-glucopyranoside (**3b**)


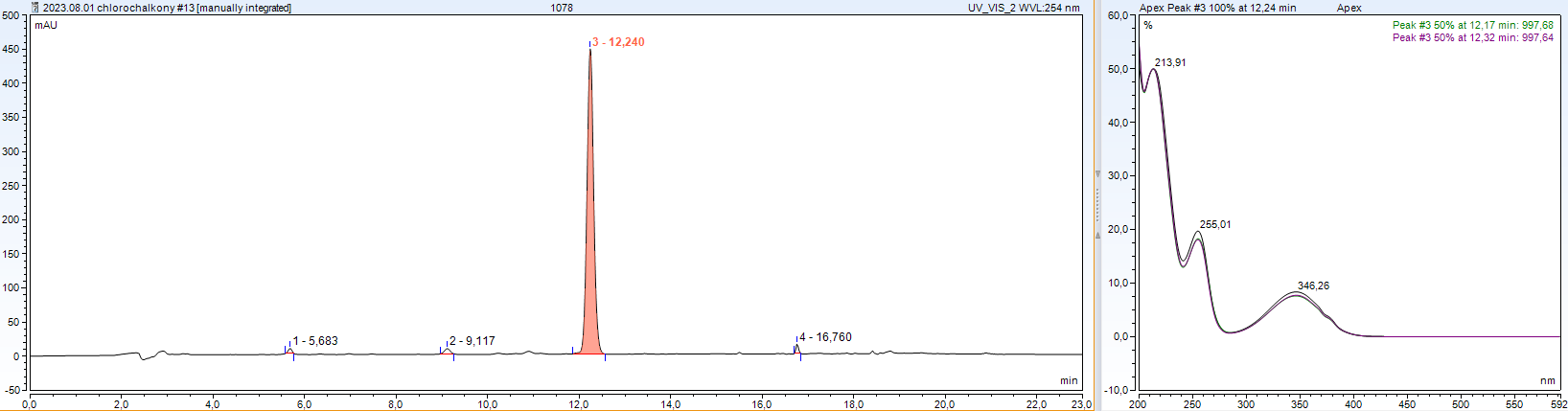


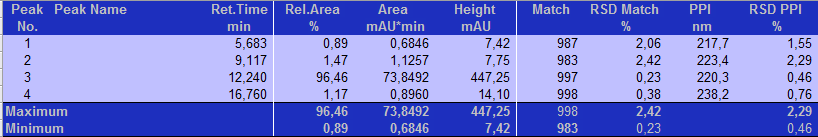


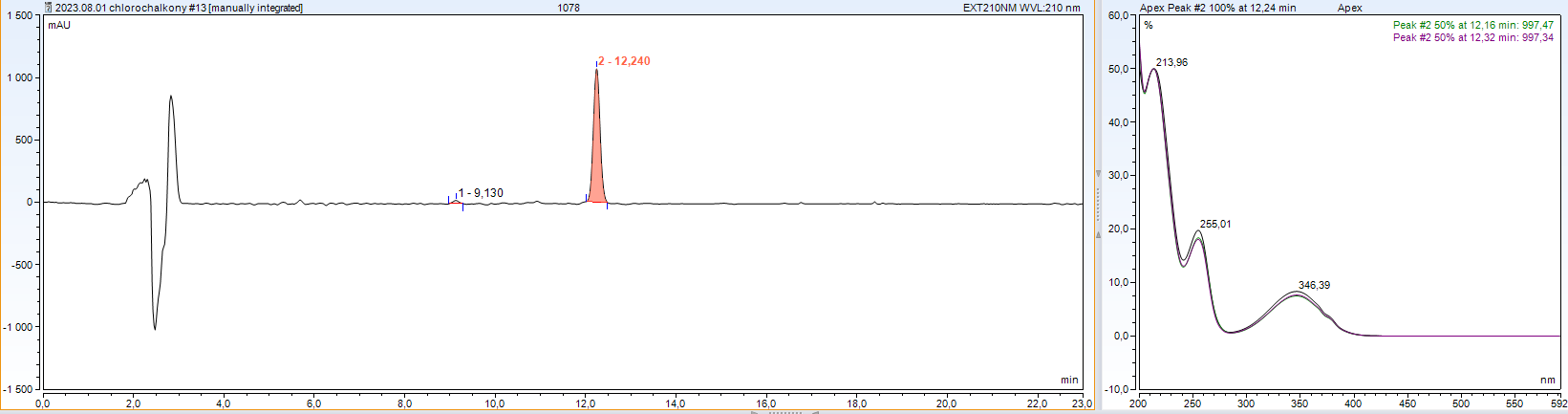


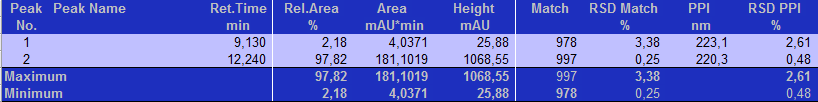


**Figure S63.** HPLC analysis of 2-chloro-2’-hydroxydihydrochalcone 5’-*O*-*β*-D-(4’’-*O*-methyl)-glucopyranoside (**3b**)


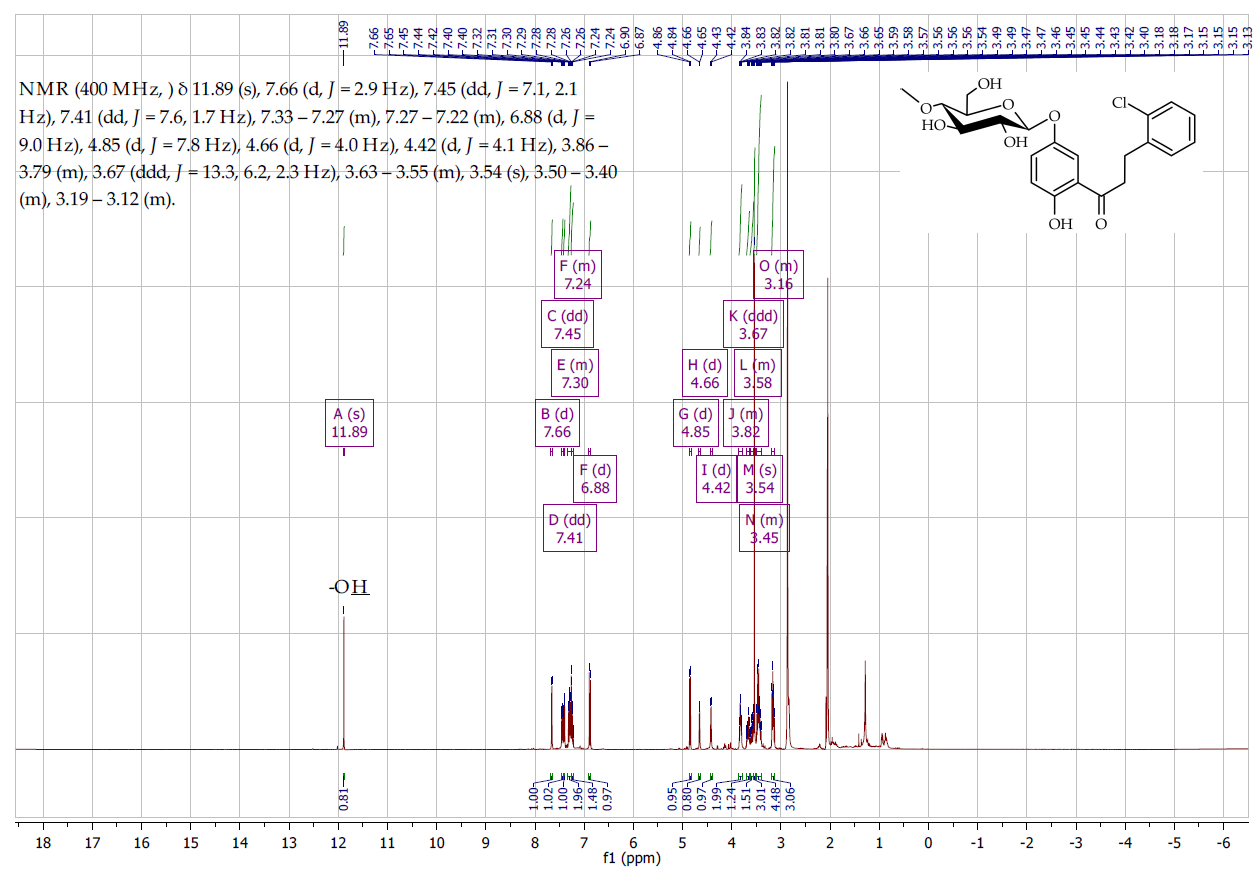


**Figure S64.** ^1^H NMR spectrum (*δ,* acetone-d6, 600 MHz) of 2-chloro-2’-hydroxydihydrochalcone 5’-*O*-*β*-D-(4’’-*O*-methyl)-glucopyranoside (**3b**)


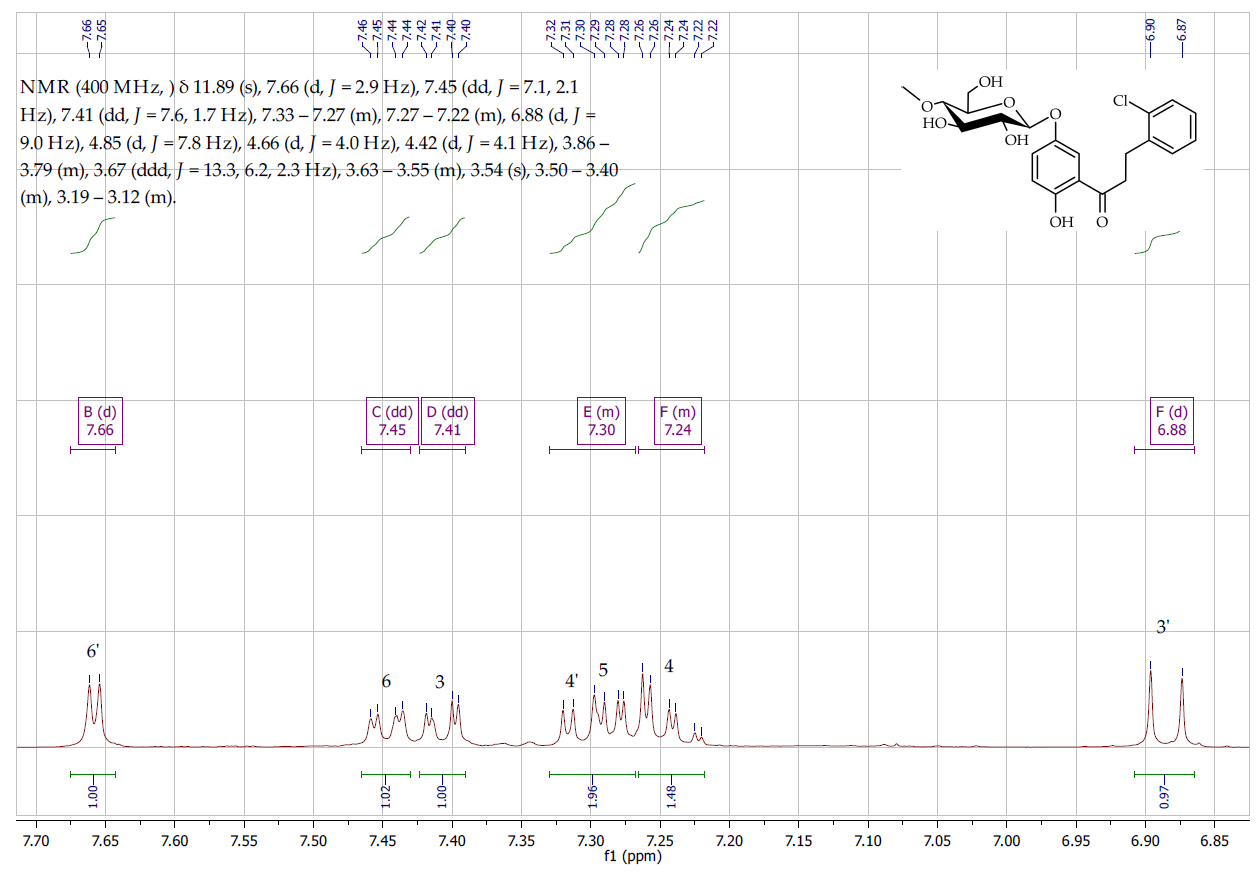


**Figure S65** ^1^H NMR spectrum expansion (*δ,* acetone-d6, 600 MHz) of 2-chloro-2’-hydroxydihydrochalcone 5’-*O*-*β*-D-(4’’-*O*-methyl)-glucopyranoside (**3b**)

**Figure S66** ^1^H NMR spectrum expansion (*δ,* acetone-d6, 600 MHz) of 2-chloro-2’-hydroxydihydrochalcone 5’-*O*-*β*-D-(4’’-*O*-methyl)-glucopyranoside (**3b**)

**Figure S67.** ^13^C NMR spectrum (*δ,* acetone-d6, 151 MHz) of 2-chloro-2’-hydroxydihydrochalcone 5’-*O*-*β*-D-(4’’-*O*-methyl)-glucopyranoside (**3b**)

**Figure S68.** ^13^C NMR spectrum expansion (*δ,* acetone-d6, 151 MHz) of 2-chloro-2’-hydroxydihydrochalcone 5’-*O*-*β*-D-(4’’-*O*-methyl)-glucopyranoside (**3b**)

**Figure S69.** ^13^C NMR spectrum expansion (*δ,* acetone-d6, 151 MHz) of 2-chloro-2’-hydroxydihydrochalcone 5’-*O*-*β*-D-(4’’-*O*-methyl)-glucopyranoside (**3b**)

**Figure S70.** ^13^C NMR spectrum expansion (*δ,* acetone-d6, 151 MHz) of 2-chloro-2’-hydroxydihydrochalcone 5’-*O*-*β*-D-(4’’-*O*-methyl)-glucopyranoside (**3b**)

**Figure S71.** COSY contour map – ^1^H x ^1^H of 2-chloro-2’-hydroxydihydrochalcone 5’-*O*-*β*-D-(4’’-*O*-methyl)-glucopyranoside (**3b**)

**Figure S72.** COSY contour map – ^1^H x ^1^H expansion of 2-chloro-2’-hydroxydihydrochalcone 5’-*O*-*β*-D-(4’’-*O*-methyl)-glucopyranoside (**3b**)

**Figure S73.** COSY contour map – ^1^H x ^1^H expansion of 2-chloro-2’-hydroxydihydrochalcone 5’-*O*-*β*-D-(4’’-*O*-methyl)-glucopyranoside (**3b**)

**Figure S74.** HMQC contour map – ^1^H x ^13^C of 2-chloro-2’-hydroxydihydrochalcone 5’-*O*-*β*-D-(4’’-*O*-methyl)-glucopyranoside (**3b**)

**Figure S75.** HMQC contour map – ^1^H x ^13^C expansion of 2-chloro-2’-hydroxydihydrochalcone 5’-*O*-*β*-D-(4’’-*O*-methyl)-glucopyranoside (**3b**)

**Figure S76.** HMQC contour map – ^1^H x ^13^C expansion of 2-chloro-2’-hydroxydihydrochalcone 5’-*O*-*β*-D-(4’’-*O*-methyl)-glucopyranoside (**3b**)

**Figure S77.** HMBC contour map – ^1^H x ^13^C of 2-chloro-2’-hydroxydihydrochalcone 5’-*O*-*β*-D-(4’’-*O*-methyl)-glucopyranoside (**3b**)

**Figure S78.** HMBC contour map – ^1^H x ^13^C expansion of 2-chloro-2’-hydroxydihydrochalcone 5’-*O*-*β*-D-(4’’-*O*-methyl)-glucopyranoside (**3b**)

**Figure S79.** HMBC contour map – ^1^H x ^13^C expansion of 2-chloro-2’-hydroxydihydrochalcone 5’-*O*-*β*-D-(4’’-*O*-methyl)-glucopyranoside (**3b**)

**Figure S80.** HMBC contour map – ^1^H x ^13^C expansion of 2-chloro-2’-hydroxydihydrochalcone 5’-*O*-*β*-D-(4’’-*O*-methyl)-glucopyranoside (**3b**)

**Figure S81.** HMBC contour map – ^1^H x ^13^C expansion of 2-chloro-2’-hydroxydihydrochalcone 5’-*O*-*β*-D-(4’’-*O*-methyl)-glucopyranoside (**3b**)

**Figure S82.** HMBC contour map – ^1^H x ^13^C expansion of 2-chloro-2’-hydroxydihydrochalcone 5’-*O*-*β*-D-(4’’-*O*-methyl)-glucopyranoside (**3b**)

**Figure S83.** 2-Chloro-2’-hydroxydihydrochalcone 5’-*O*-*β*-D-(4’’-*O*-methyl)-glucopyranoside (**3b**) physicochemical and ADME parameters prediction using the SwissADME modelling

**Figure S84.** 2-Chloro-2’-hydroxydihydrochalcone 5’-*O*-*β*-D-(4’’-*O*-methyl)-glucopyranoside (**3b**) physicochemical biological activity prediction using the Way2Drug Pass online modelling

**Figure S85.** 2-Chloro-2’-hydroxydihydrochalcone 5’-*O*-*β*-D-(4’’-*O*-methyl)-glucopyranoside (**3b**) antibacterial activity prediction using the Way2Drug AntiBac-Pred modelling

**Figure S86.** 2-Chloro-2’-hydroxydihydrochalcone 5’-*O*-*β*-D-(4’’-*O*-methyl)-glucopyranoside (**3b**) antifungal activity prediction using the Way2Drug AntiFun-Pred modelling

**Figure S87.** 2-Chloro-2’-hydroxydihydrochalcone 5’-*O*-*β*-D-(4’’-*O*-methyl)-glucopyranoside (**3b**) antiviral activity prediction using the Way2Drug AntiVir-Pred modelling

**Figure S88.** MS analysis of 2-chloro-2’,3-dihydroxydihydrochalcone 3’-*O*-*β*-D-(4’’-*O*-methyl)-glucopyranoside (**3c**)

**Figure S89.** HPLC analysis of 2-chloro-2’,3-dihydroxydihydrochalcone 3’-*O*-*β*-D-(4’’-*O*-methyl)-glucopyranoside (**3c**)

**Figure S90.** ^1^H NMR spectrum (*δ,* acetone-d6, 600 MHz) of 2-chloro-2’,3-dihydroxydihydrochalcone 3’-*O*-*β*-D-(4’’-*O*-methyl)-glucopyranoside (**3c**)

**Figure S91.** ^1^H NMR spectrum expansion (*δ,* acetone-d6, 600 MHz) of 2-chloro-2’,3-dihydroxydihydrochalcone 3’-*O*-*β*-D-(4’’-*O*-methyl)-glucopyranoside (**3c**)

**Figure S92.** ^1^H NMR spectrum expansion (*δ,* acetone-d6, 600 MHz) of 2-chloro-2’,3-dihydroxydihydrochalcone 3’-*O*-*β*-D-(4’’-*O*-methyl)-glucopyranoside (**3c**)

**Figure S93.** ^13^C NMR spectrum (*δ,* acetone-d6, 151 MHz) of 2-chloro-2’,3-dihydroxydihydrochalcone 3’-*O*-*β*-D-(4’’-*O*-methyl)-glucopyranoside (**3c**)

**Figure S94.** ^13^C NMR spectrum expansion (*δ,* acetone-d6, 151 MHz) of 2-chloro-2’,3-dihydroxydihydrochalcone 3’-*O*-*β*-D-(4’’-*O*-methyl)-glucopyranoside (**3c**)

**Figure S95.** ^13^C NMR spectrum expansion (*δ,* acetone-d6, 151 MHz) of 2-chloro-2’,3-dihydroxydihydrochalcone 3’-*O*-*β*-D-(4’’-*O*-methyl)-glucopyranoside (**3c**)

**Figure S96.** COSY contour map – ^1^H x ^1^H of 2-chloro-2’,3-dihydroxydihydrochalcone 3’-*O*-*β*-D-(4’’-*O*-methyl)-glucopyranoside (**3c**)

**Figure S97.** COSY contour map – ^1^H x ^1^H expansion of 2-chloro-2’,3-dihydroxydihydrochalcone 3’-*O*-*β*-D-(4’’-*O*-methyl)-glucopyranoside (**3c**)

**Figure S98.** COSY contour map – ^1^H x ^1^H expansion of 2-chloro-2’,3-dihydroxydihydrochalcone 3’-*O*-*β*-D-(4’’-*O*-methyl)-glucopyranoside (**3c**)

**Figure S99.** HMQC contour map – ^1^H x ^13^C of 2-chloro-2’,3-dihydroxydihydrochalcone 3’-*O*-*β*-D-(4’’-*O*-methyl)-glucopyranoside (**3c**)

**Figure S100.** HMQC contour map – ^1^H x ^13^C expansion of 2-chloro-2’,3-dihydroxydihydrochalcone 3’-*O*-*β*-D-(4’’-*O*-methyl)-glucopyranoside (**3c**)

**Figure S101.** HMQC contour map – ^1^H x ^13^C expansion of 2-chloro-2’,3-dihydroxydihydrochalcone 3’-*O*-*β*-D-(4’’-*O*-methyl)-glucopyranoside (**3c**)

**Figure S102.** HMBC contour map – ^1^H x ^13^C of 2-chloro-2’,3-dihydroxydihydrochalcone 3’-*O*-*β*-D-(4’’-*O*-methyl)-glucopyranoside (**3c**)

**Figure S103.** HMBC contour map – ^1^H x ^13^C expansion of 2-chloro-2’,3-dihydroxydihydrochalcone 3’-*O*-*β*-D-(4’’-*O*-methyl)-glucopyranoside (**3c**)

**Figure S104.** HMBC contour map – ^1^H x ^13^C expansion of 2-chloro-2’,3-dihydroxydihydrochalcone 3’-*O*-*β*-D-(4’’-*O*-methyl)-glucopyranoside (**3c**)

**Figure S105.** HMBC contour map – ^1^H x ^13^C expansion of 2-chloro-2’,3-dihydroxydihydrochalcone 3’-*O*-*β*-D-(4’’-*O*-methyl)-glucopyranoside (**3c**)

**Figure S106.** HMBC contour map – ^1^H x ^13^C expansion of 2-chloro-2’,3-dihydroxydihydrochalcone 3’-*O*-*β*-D-(4’’-*O*-methyl)-glucopyranoside (**3c**)

**Figure S107.** HMBC contour map – ^1^H x ^13^C expansion of 2-chloro-2’,3-dihydroxydihydrochalcone 3’-*O*-*β*-D-(4’’-*O*-methyl)-glucopyranoside (**3c**)

**Figure S108.** 2-Chloro-2’,3-dihydroxydihydrochalcone 3’-*O*-*β*-D-(4’’-*O*-methyl)-glucopyranoside (**3c**) physicochemical and ADME parameters prediction using the SwissADME modelling

**Figure S109.** 2-Chloro-2’,3-dihydroxydihydrochalcone 3’-*O*-*β*-D-(4’’-*O*-methyl)-glucopyranoside (**3c**) physicochemical biological activity prediction using the Way2Drug Pass online modelling

**Figure S110.** 2-Chloro-2’,3-dihydroxydihydrochalcone 3’-*O*-*β*-D-(4’’-*O*-methyl)-glucopyranoside (**3c**) antibacterial activity prediction using the Way2Drug AntiBac-Pred modelling

**Figure S111.** 2-Chloro-2’,3-dihydroxydihydrochalcone 3’-*O*-*β*-D-(4’’-*O*-methyl)-glucopyranoside (**3c**) antifungal activity prediction using the Way2Drug AntiFun-Pred modelling

**Figure S112.** 2-Chloro-2’,3-dihydroxydihydrochalcone 3’-*O*-*β*-D-(4’’-*O*-methyl)-glucopyranoside (**3c**) antiviral activity prediction using the Way2Drug AntiVir-Pred modelling

**Figure S113.** MS analysis of 3-chlorodihydrochalcone 2’-*O*-*β*-D-(4’’-*O*-methyl)-glucopyranoside (**5a**)

**Figure S114.** HPLC analysis of 3-chlorodihydrochalcone 2’-*O*-*β*-D-(4’’-*O*-methyl)-glucopyranoside (**5a**)

**Figure S115.** ^1^H NMR spectrum (*δ,* acetone-d6, 600 MHz) of 3-chlorodihydrochalcone 2’-*O*-*β*-D-(4’’-*O*-methyl)-glucopyranoside (**5a**)

**Figure S116** ^1^H NMR spectrum expansion (*δ,* acetone-d6, 600 MHz) of 3-chlorodihydrochalcone 2’-*O*-*β*-D-(4’’-*O*-methyl)-glucopyranoside (**5a**)

**Figure S117** ^1^H NMR spectrum expansion (*δ,* acetone-d6, 600 MHz) of 3-chlorodihydrochalcone 2’-*O*-*β*-D-(4’’-*O*-methyl)-glucopyranoside (**5a**)

**Figure S118.** ^13^C NMR spectrum (*δ,* acetone-d6, 151 MHz) of 3-chlorodihydrochalcone 2’-*O*-*β*-D-(4’’-*O*-methyl)-glucopyranoside (**5a**)

**Figure S119.** ^13^C NMR spectrum expansion (*δ,* acetone-d6, 151 MHz) of 3-chlorodihydrochalcone 2’-*O*-*β*-D-(4’’-*O*-methyl)-glucopyranoside (**5a**)

**Figure S120.** ^13^C NMR spectrum expansion (*δ,* acetone-d6, 151 MHz) of 3-chlorodihydrochalcone 2’-*O*-*β*-D-(4’’-*O*-methyl)-glucopyranoside (**5a**)

**Figure S121.** COSY contour map – ^1^H x ^1^H of 3-chlorodihydrochalcone 2’-*O*-*β*-D-(4’’-*O*-methyl)-glucopyranoside (**5a**)

**Figure S122.** COSY contour map – ^1^H x ^1^H expansion of 3-chlorodihydrochalcone 2’-*O*-*β*-D-(4’’-*O*-methyl)-glucopyranoside (**5a**)

**Figure S123.** COSY contour map – ^1^H x ^1^H expansion of 3-chlorodihydrochalcone 2’-*O*-*β*-D-(4’’-*O*-methyl)-glucopyranoside (**5a**)

**Figure S124.** HMQC contour map – ^1^H x ^13^C of 3-chlorodihydrochalcone 2’-*O*-*β*-D-(4’’-*O*-methyl)-glucopyranoside (**5a**)

**Figure S125.** HMQC contour map – ^1^H x ^13^C expansion of 3-chlorodihydrochalcone 2’-*O*-*β*-D-(4’’-*O*-methyl)-glucopyranoside (**5a**)

**Figure S126.** HMQC contour map – ^1^H x ^13^C expansion of 3-chlorodihydrochalcone 2’-*O*-*β*-D-(4’’-*O*-methyl)-glucopyranoside (**5a**)

**Figure S127.** HMBC contour map – ^1^H x ^13^C of 3-chlorodihydrochalcone 2’-*O*-*β*-D-(4’’-*O*-methyl)-glucopyranoside (**5a**)

**Figure S128.** HMBC contour map – ^1^H x ^13^C expansion of 3-chlorodihydrochalcone 2’-*O*-*β*-D-(4’’-*O*-methyl)-glucopyranoside (**5a**)

**Figure S129.** HMBC contour map – ^1^H x ^13^C expansion of 3-chlorodihydrochalcone 2’-*O*-*β*-D-(4’’-*O*-methyl)-glucopyranoside (**5a**)

**Figure S130.** HMBC contour map – ^1^H x ^13^C expansion of 3-chlorodihydrochalcone 2’-*O*-*β*-D-(4’’-*O*-methyl)-glucopyranoside (**5a**)

**Figure S131.** HMBC contour map – ^1^H x ^13^C expansion of 3-chlorodihydrochalcone 2’-*O*-*β*-D-(4’’-*O*-methyl)-glucopyranoside (**5a**)

**Figure S132.** HMBC contour map – ^1^H x ^13^C expansion of 3-chlorodihydrochalcone 2’-*O*-*β*-D-(4’’-*O*-methyl)-glucopyranoside (**5a**)

**Figure S133.** 3-Chlorodihydrochalcone 2’-*O*-*β*-D-(4’’-*O*-methyl)-glucopyranoside (**5a**) physicochemical and ADME parameters prediction using the SwissADME modelling

**Figure S134.** 3-Chlorodihydrochalcone 2’-*O*-*β*-D-(4’’-*O*-methyl)-glucopyranoside (**5a**) physicochemical biological activity prediction using the Way2Drug Pass online modelling

**Figure S135.** 3-Chlorodihydrochalcone 2’-*O*-*β*-D-(4’’-*O*-methyl)-glucopyranoside (**5a**) antibacterial activity prediction using the Way2Drug AntiBac-Pred modelling

**Figure S136.** 3-Chlorodihydrochalcone 2’-*O*-*β*-D-(4’’-*O*-methyl)-glucopyranoside (**5a**) antifungal activity prediction using the Way2Drug AntiFun-Pred modelling

**Figure S137.** 3-Chlorodihydrochalcone 2’-*O*-*β*-D-(4’’-*O*-methyl)-glucopyranoside (**5a**) antiviral activity prediction using the Way2Drug AntiVir-Pred modelling

**Figure S138.** MS analysis of 3-chloro-2’-hydroxydihydrochalcone 5’-*O*-*β*-D-(4’’-*O*-methyl)-glucopyranoside (**5b**)

**Figure S139.** HPLC analysis of 3-chloro-2’-hydroxydihydrochalcone 5’-*O*-*β*-D-(4’’-*O*-methyl)-glucopyranoside (**5b**)

**Figure S140.** ^1^H NMR spectrum (*δ,* acetone-d6, 600 MHz) of 3-chloro-2’-hydroxydihydrochalcone 5’-*O*-*β*-D-(4’’-*O*-methyl)-glucopyranoside (**5b**)

**Figure S141.** ^1^H NMR spectrum expansion (*δ,* acetone-d6, 600 MHz) of 3-chloro-2’-hydroxydihydrochalcone 5’-*O*-*β*-D-(4’’-*O*-methyl)-glucopyranoside (**5b**)

**Figure S142.** ^1^H NMR spectrum expansion (*δ,* acetone-d6, 600 MHz) of 3-chloro-2’-hydroxydihydrochalcone 5’-*O*-*β*-D-(4’’-*O*-methyl)-glucopyranoside (**5b**)

**Figure S143.** ^13^C NMR spectrum (*δ,* acetone-d6, 151 MHz) of 3-chloro-2’-hydroxydihydrochalcone 5’-*O*-*β*-D-(4’’-*O*-methyl)-glucopyranoside (**5b**)

**Figure S144.** ^13^C NMR spectrum expansion (*δ,* acetone-d6, 151 MHz) of 3-chloro-2’-hydroxydihydrochalcone 5’-*O*-*β*-D-(4’’-*O*-methyl)-glucopyranoside (**5b**)

**Figure S145.** ^13^C NMR spectrum expansion (*δ,* acetone-d6, 151 MHz) of 3-chloro-2’-hydroxydihydrochalcone 5’-*O*-*β*-D-(4’’-*O*-methyl)-glucopyranoside (**5b**)

**Figure S146.** COSY contour map – ^1^H x ^1^H of 3-chloro-2’-hydroxydihydrochalcone 5’-*O*-*β*-D-(4’’-*O*-methyl)-glucopyranoside (**5b**)

**Figure S147.** COSY contour map – ^1^H x ^1^H expansion of 3-chloro-2’-hydroxydihydrochalcone 5’-*O*-*β*-D-(4’’-*O*-methyl)-glucopyranoside (**5b**)

**Figure S148.** COSY contour map – ^1^H x ^1^H expansion of 3-chloro-2’-hydroxydihydrochalcone 5’-*O*-*β*-D-(4’’-*O*-methyl)-glucopyranoside (**5b**)

**Figure S149.** HMQC contour map – ^1^H x ^13^C of 3-chloro-2’-hydroxydihydrochalcone 5’-*O*-*β*-D-(4’’-*O*-methyl)-glucopyranoside (**5b**)

**Figure S150.** HMQC contour map – ^1^H x ^13^C expansion of 3-chloro-2’-hydroxydihydrochalcone 5’-*O*-*β*-D-(4’’-*O*-methyl)-glucopyranoside (**5b**)

**Figure S151.** HMQC contour map – ^1^H x ^13^C expansion of 3-chloro-2’-hydroxydihydrochalcone 5’-*O*-*β*-D-(4’’-*O*-methyl)-glucopyranoside (**5b**)

**Figure S152.** HMBC contour map – ^1^H x ^13^C of 3-chloro-2’-hydroxydihydrochalcone 5’-*O*-*β*-D-(4’’-*O*-methyl)-glucopyranoside (**5b**)

**Figure S153.** HMBC contour map – ^1^H x ^13^C expansion of 3-chloro-2’-hydroxydihydrochalcone 5’-*O*-*β*-D-(4’’-*O*-methyl)-glucopyranoside (**5b**)

**Figure S154.** HMBC contour map – ^1^H x ^13^C expansion of 3-chloro-2’-hydroxydihydrochalcone 5’-*O*-*β*-D-(4’’-*O*-methyl)-glucopyranoside (**5b**)

**Figure S155.** HMBC contour map – ^1^H x ^13^C expansion of 3-chloro-2’-hydroxydihydrochalcone 5’-*O*-*β*-D-(4’’-*O*-methyl)-glucopyranoside (**5b**)

**Figure S156.** HMBC contour map – ^1^H x ^13^C expansion of 3-chloro-2’-hydroxydihydrochalcone 5’-*O*-*β*-D-(4’’-*O*-methyl)-glucopyranoside (**5b**)

**Figure S157.** HMBC contour map – ^1^H x ^13^C expansion of 3-chloro-2’-hydroxydihydrochalcone 5’-*O*-*β*-D-(4’’-*O*-methyl)-glucopyranoside (**5b**)

**Figure S158.** 3-Chloro-2’-hydroxydihydrochalcone 5’-*O*-*β*-D-(4’’-*O*-methyl)-glucopyranoside (**5b**) physicochemical and ADME parameters prediction using the SwissADME modelling

**Figure S159.** 3-Chloro-2’-hydroxydihydrochalcone 5’-*O*-*β*-D-(4’’-*O*-methyl)-glucopyranoside (**5b**) physicochemical biological activity prediction using the Way2Drug Pass online modelling

**Figure S160.** 3-Chloro-2’-hydroxydihydrochalcone 5’-*O*-*β*-D-(4’’-*O*-methyl)-glucopyranoside (**5b**) antibacterial activity prediction using the Way2Drug AntiBac-Pred modelling

**Figure S161.** 3-Chloro-2’-hydroxydihydrochalcone 5’-*O*-*β*-D-(4’’-*O*-methyl)-glucopyranoside (**5b**) antifungal activity prediction using the Way2Drug AntiFun-Pred modelling

**Figure S162.** 3-Chloro-2’-hydroxydihydrochalcone 5’-*O*-*β*-D-(4’’-*O*-methyl)-glucopyranoside (**5b**) antiviral activity prediction using the Way2Drug AntiVir-Pred modelling

**Figure S163.** MS analysis of 3-chloro-2’-hydroxydihydrochalcone 4-*O*-*β*-D-(4’’-*O*-methyl)-glucopyranoside (**5c**)

**Figure S164.** HPLC analysis of 3-chloro-2’-hydroxydihydrochalcone 4-*O*-*β*-D-(4’’-*O*-methyl)-glucopyranoside (**5c**)

**Figure S165.** ^1^H NMR spectrum (*δ,* acetone-d6, 600 MHz) of 3-chloro-2’-hydroxydihydrochalcone 4-*O*-*β*-D-(4’’-*O*-methyl)-glucopyranoside (**5c**)

**Figure S166.** ^1^H NMR spectrum expansion (*δ,* acetone-d6, 600 MHz) of 3-chloro-2’-hydroxydihydrochalcone 4-*O*-*β*-D-(4’’-*O*-methyl)-glucopyranoside (**5c**)

**Figure S167.** ^1^H NMR spectrum expansion (*δ,* acetone-d6, 600 MHz) of 3-chloro-2’-hydroxydihydrochalcone 4-*O*-*β*-D-(4’’-*O*-methyl)-glucopyranoside (**5c**)

**Figure S168.** ^13^C NMR spectrum (*δ,* acetone-d6, 151 MHz) of 3-chloro-2’-hydroxydihydrochalcone 4-*O*-*β*-D-(4’’-*O*-methyl)-glucopyranoside (**5c**)

**Figure S169.** ^13^C NMR spectrum expansion (*δ,* acetone-d6, 151 MHz) of 3-chloro-2’-hydroxydihydrochalcone 4-*O*-*β*-D-(4’’-*O*-methyl)-glucopyranoside (**5c**)

**Figure S170.** ^13^C NMR spectrum expansion (*δ,* acetone-d6, 151 MHz) of 3-chloro-2’-hydroxydihydrochalcone 4-*O*-*β*-D-(4’’-*O*-methyl)-glucopyranoside (**5c**)

**Figure S171.** COSY contour map – ^1^H x ^1^H of 3-chloro-2’-hydroxydihydrochalcone 4-*O*-*β*-D-(4’’-*O*-methyl)-glucopyranoside (**5c**)

**Figure S172.** COSY contour map – ^1^H x ^1^H expansion of 3-chloro-2’-hydroxydihydrochalcone 4-*O*-*β*-D-(4’’-*O*-methyl)-glucopyranoside (**5c**)

**Figure S173.** COSY contour map – ^1^H x ^1^H expansion of 3-chloro-2’-hydroxydihydrochalcone 4-*O*-*β*-D-(4’’-*O*-methyl)-glucopyranoside (**5c**)

**Figure S174.** HMQC contour map – ^1^H x ^13^C of 3-chloro-2’-hydroxydihydrochalcone 4-*O*-*β*-D-(4’’-*O*-methyl)-glucopyranoside (**5c**)

**Figure S175.** HMQC contour map – ^1^H x ^13^C expansion of 3-chloro-2’-hydroxydihydrochalcone 4-*O*-*β*-D-(4’’-*O*-methyl)-glucopyranoside (**5c**)

**Figure S176.** HMQC contour map – ^1^H x ^13^C expansion of 3-chloro-2’-hydroxydihydrochalcone 4-*O*-*β*-D-(4’’-*O*-methyl)-glucopyranoside (**5c**)

**Figure S177.** HMBC contour map – ^1^H x ^13^C of 3-chloro-2’-hydroxydihydrochalcone 4-*O*-*β*-D-(4’’-*O*-methyl)-glucopyranoside (**5c**)

**Figure S178.** HMBC contour map – ^1^H x ^13^C expansion of 3-chloro-2’-hydroxydihydrochalcone 4-*O*-*β*-D-(4’’-*O*-methyl)-glucopyranoside (**5c**)

**Figure S179.** HMBC contour map – ^1^H x ^13^C expansion of 3-chloro-2’-hydroxydihydrochalcone 4-*O*-*β*-D-(4’’-*O*-methyl)-glucopyranoside (**5c**)

**Figure S180.** HMBC contour map – ^1^H x ^13^C expansion of 3-chloro-2’-hydroxydihydrochalcone 4-*O*-*β*-D-(4’’-*O*-methyl)-glucopyranoside (**5c**)

**Figure S181.** HMBC contour map – ^1^H x ^13^C expansion of 3-chloro-2’-hydroxydihydrochalcone 4-*O*-*β*-D-(4’’-*O*-methyl)-glucopyranoside (**5c**)

**Figure S182.** HMBC contour map – ^1^H x ^13^C expansion of 3-chloro-2’-hydroxydihydrochalcone 4-*O*-*β*-D-(4’’-*O*-methyl)-glucopyranoside (**5c**)

**Figure S183.** 3-Chloro-2’-hydroxydihydrochalcone 4-*O*-*β*-D-(4’’-*O*-methyl)-glucopyranoside (**5c**) physicochemical and ADME parameters prediction using the SwissADME modelling

**Figure S184.** 3-Chloro-2’-hydroxydihydrochalcone 4-*O*-*β*-D-(4’’-*O*-methyl)-glucopyranoside (**5c**) physicochemical biological activity prediction using the Way2Drug Pass online modelling

**Figure S185.** 3-Chloro-2’-hydroxydihydrochalcone 4-*O*-*β*-D-(4’’-*O*-methyl)-glucopyranoside (**5c**) antibacterial activity prediction using the Way2Drug AntiBac-Pred modelling

**Figure S186.** 3-Chloro-2’-hydroxydihydrochalcone 4-*O*-*β*-D-(4’’-*O*-methyl)-glucopyranoside (**5c**) antifungal activity prediction using the Way2Drug AntiFun-Pred modelling

**Figure S187.** 3-Chloro-2’-hydroxydihydrochalcone 4-*O*-*β*-D-(4’’-*O*-methyl)-glucopyranoside (**5c**) antiviral activity prediction using the Way2Drug AntiVir-Pred modelling

**Figure S188.** 2’-Hydroxychalcone (**6**) physicochemical and ADME parameters prediction using the SwissADME modelling

**Figure S189.** 2’-Hydroxychalcone (**6**) physicochemical biological activity prediction using the Way2Drug Pass online modelling

**Figure S190.** 2’-Hydroxychalcone (**6**) antibacterial activity prediction using the Way2Drug AntiBac-Pred modelling

**Figure S191.** 2’-Hydroxychalcone (**6**) antifungal activity prediction using the Way2Drug AntiFun-Pred modelling

**Figure S192.** 2’-Hydroxychalcone (**6**) antiviral activity prediction using the Way2Drug AntiVir-Pred modelling
